# Candidate Metabolic Biomarkers for Physical Fatigue Assessment: An Integrative Meta-Analysis and Exploratory Liquid Chromatography–Mass Spectrometry Pilot Study of Acute Exercise Responses

**DOI:** 10.3390/metabo16070509

**Published:** 2026-07-21

**Authors:** Lintao Huang, Wenhui Yang, Pengyu Fu, Xingrong Li, Menglu Pi, Ruilin Shi, Xuehao Wang, Hangfei Qi, Chunyu Zhu, Zhonghuan Jiang, Zifeng Yue, Hange Guo, Jiashuai Tong, Zhihao Chen, Ye Tian, Airong Qian

**Affiliations:** 1 School of Life Science and Technology, Northwestern Polytechnical University, Xi’an 710072, China; huanglintao@mail.nwpu.edu.cn (L.H.); yangwenhui@mail.nwpu.edu.cn (W.Y.); lixingrong@mail.nwpu.edu.cn (X.L.); pimenglu@mail.nwpu.edu.cn (M.P.); flora2021@mail.nwpu.edu.cn (R.S.); wxuehao@mail.nwpu.edu.cn (X.W.); qihangfei@mail.nwpu.edu.cn (H.Q.); zcy@nwpu.edu.cn (C.Z.); jiangyao4422@mail.nwpu.edu.cn (Z.J.); yuezifeng6@mail.nwpu.edu.cn (Z.Y.); guohange@mail.nwpu.edu.cn (H.G.); tjs20040727@mail.nwpu.edu.cn (J.T.); 2 Terahertz Biomedical Interdisciplinary Joint Research Center, Xi’an 710072, China; 3 Xi’an Key Laboratory of Special Medicine and Health Engineering, Xi’an 710072, China; 4 Key Lab for Space Biosciences and Biotechnology, Xi’an 710072, China; 5 Department of Physical Education, Northwestern Polytechnical University, Xi’an 710072, China; fupy@nwpu.edu.cn

**Keywords:** body fluid, LC-MS, meta-analysis, metabolomics, physical fatigue biomarkers

## Abstract

Background: Objective body-fluid biomarkers may help characterize exercise-induced physical fatigue; however, heterogeneity in exercise protocols, biological matrices, and analytical platforms complicates biomarker interpretation. Purpose: This study aimed to identify candidate acute exercise-responsive metabolic biomarkers associated with physical fatigue assessment by integrating conventional biomarker evidence, exercise-related metabolomics, and exploratory targeted Liquid chromatography–mass spectrometry (LC-MS) analysis. Methods: We conducted three complementary analyses: (1) a systematic review and random-effects meta-analysis of quantitative biomarkers measured in body-fluid matrices, including blood, plasma, serum, saliva, sweat, urine, and interstitial fluid, at pre-exercise and post-exercise time points; (2) a descriptive fold-change-based synthesis of exercise-related metabolomics studies; and (3) an exploratory paired plasma–saliva liquid chromatography–mass spectrometry pilot analysis in seven apparently healthy young male volunteers. Eligible studies included apparently healthy human participants without reported acute or chronic diseases, major injuries, pregnancy, or clinically diagnosed pathological fatigue, undergoing endurance, resistance, high-intensity interval, or mixed exercise protocols. Biomarkers reported in ≥10 independent comparisons were pooled using log-transformed post-exercise/pre-exercise response ratios. Results: The systematic review and meta-analysis included 110 articles, 129 experiments, and 1826 participants. Lactate showed the strongest pooled response after exercise (response ratio = 4.43, 95% CI: 3.74–5.25, *p* < 0.00001; I^2^ = 98%), followed by IL-6 (2.15, 95% CI: 1.50–3.07, *p* < 0.0001; I^2^ = 87%), CK (1.70, 95% CI: 1.56–1.86, *p* < 0.00001; I^2^ = 90%), and LDH (1.23, 95% CI: 1.11–1.35, *p* < 0.0001; I^2^ = 93%). Metabolomics synthesis identified lactate, pyruvate, hypoxanthine and xanthine as frequently reported exercise-responsive metabolites related to glycolysis and purine degradation. Exploratory LC-MS analysis showed consistent directional changes in these metabolites in paired plasma and saliva samples. Conclusions: Lactate, pyruvate, hypoxanthine, and xanthine represent candidate metabolism-based acute exercise-responsive biomarkers associated with physical fatigue. However, their fatigue specificity remains to be established and requires validation using independent fatigue criteria, non-fatiguing exercise controls, matrix-specific analyses, and adequately powered diverse cohorts.

## 1. Introduction

Physical fatigue is a common physiological state that develops after prolonged or high-intensity physical activity and is characterized by reduced force-generating capacity, impaired motor performance, delayed reaction, decreased attention, and subjective exhaustion [1]. It arises from coordinated changes in energy metabolism, neuromuscular function, inflammatory signaling, endocrine activity, and central nervous system regulation [2,3]. Beyond athletic performance, physical fatigue is closely related to public health and occupational safety. In sports, military, firefighting, transportation, and emergency medical settings, fatigue increases the risk of overtraining, delayed recovery, musculoskeletal injury, operational errors, and safety-related incidents [4,5,6,7,8,9,10,11]. Reliable assessment of physical fatigue is therefore important for training-load management, recovery evaluation, injury prevention, and risk control in physically demanding populations.

Current fatigue assessment approaches include subjective questionnaires, rating scales, physical performance tests, and physiological signal monitoring [12]. Although widely used, these methods have practical and biological limitations. Self-reported scales are simple to administer but are affected by reporting bias, motivation, psychological state, and individual perception. Performance-based tests can capture functional decline but are influenced by skill level, learning effects, baseline fitness, and testing conditions. Physiological signals, including electroencephalography, electrocardiography, electromyography, and eye movement features, provide more objective information [13,14,15,16], yet they often require electrodes, wearable sensors, stable skin-device contact, and signal-processing procedures. During high-intensity exercise or outdoor activity, sweating, large body movements, and unstable sensor contact can reduce data quality and limit real-time applicability. These constraints underscore the need for objective, scalable, and biologically interpretable markers of physical fatigue.

Body-fluid biomarkers offer a biochemical route for fatigue assessment because they can reflect exercise-induced changes in energy supply, muscle damage, inflammation, endocrine regulation, and metabolic stress. Previous studies have reported post-exercise changes in blood, saliva, sweat, and urine biomarkers, including lactate, creatine kinase, lactate dehydrogenase, urea, glucose, cortisol, testosterone, C-reactive protein, and interleukin-6 [17,18,19,20]. Metabolomics has further expanded the candidate biomarker space by revealing exercise-related alterations in carbohydrates, tricarboxylic acid cycle intermediates, amino acids, fatty acids, acylcarnitines, and purine metabolites [21,22,23,24,25,26]. These findings suggest that physical fatigue is better represented by coordinated biochemical remodeling across multiple pathways than by a single marker.

However, the available evidence remains fragmented. Many studies involve small sample sizes, single exercise paradigms, specific biofluids, or distinct analytical platforms. Reported biomarkers vary across endurance exercise, resistance exercise, high-intensity interval training, and mixed exercise protocols. Sampling time, training status, sex distribution, detection methods, and data-reporting formats also differ substantially between studies. Consequently, the reproducibility, cross-biofluid consistency, and translational value of many proposed fatigue biomarkers remain uncertain. Traditional markers such as lactate and creatine kinase have been extensively studied [17,27], but endocrine and metabolic indicators such as cortisol, testosterone, glucose, and urea can be affected by circadian rhythm, training adaptation, nutrition, and exercise modality [28,29,30]. Meanwhile, metabolomics-derived candidates, especially metabolites related to glycolysis and purine degradation, have not been systematically integrated with conventional biomarker evidence or validated in paired invasive and non-invasive biofluids.

In this study, we integrated systematic review, random-effects meta-analysis, fold-change-based metabolomics synthesis, and exploratory targeted liquid chromatography–mass spectrometry (LC-MS) pilot analysis to identify candidate acute exercise-responsive body-fluid biomarkers associated with physical fatigue assessment. Here, physical fatigue was defined as an acute, reversible exercise-induced physiological state characterized by reduced capacity to sustain workload or maintain performance, and was distinguished from pathological fatigue associated with disease or injury. Because post-exercise biochemical alterations may reflect exercise exposure, metabolic demand, and recovery processes in addition to fatigue-related responses, the identified metabolites were interpreted as candidate acute exercise-responsive biomarkers rather than definitive fatigue-specific biomarkers.

## 2. Materials and Methods

### 2.1. Study Aim, Design and Setting

This study was designed as an integrative evidence synthesis combined with an exploratory targeted LC-MS pilot analysis. It consisted of three complementary components: (1) a systematic review and random-effects meta-analysis of conventional body-fluid biomarkers measured at pre-exercise and post-exercise time points; (2) a descriptive fold-change-based synthesis of exercise-related metabolomics studies based on reported directional metabolite changes; and (3) an exploratory targeted LC-MS pilot analysis using paired plasma and saliva samples collected at pre-exercise and post-exercise time points.

The systematic review and meta-analysis were conducted in accordance with the Preferred Reporting Items for Systematic Reviews and Meta-Analyses (PRISMA) 2020 guidelines [31]. The exploratory LC-MS pilot study was conducted at Northwestern Polytechnical University, Xi’an, China. The study protocol involving human volunteers was approved by the Medical and Laboratory Animal Ethics Committee of Northwestern Polytechnical University, China (approval number: 202402039), and was performed in accordance with the Declaration of Helsinki. Written informed consent was obtained from all participants before enrollment. This systematic review was not registered, and no registration number is available.

### 2.2. Literature Search Strategy

A systematic literature search was conducted to identify studies reporting changes in body-fluid biomarkers between pre-exercise and post-exercise states or following exercise protocols intended to induce physical fatigue in accordance with the PRISMA 2020 guidelines [31]. Four electronic databases, PubMed, Web of Science, Scopus, and Google Scholar, were searched from inception to the final search date (12 December 2025).

The search strategy was developed using a modified population, intervention, comparison, and outcome framework. The population included apparently healthy human participants without reported acute or chronic diseases, major injuries, or clinically diagnosed pathological fatigue. The intervention/exposure was acute exercise or exercise protocols intended to induce physical fatigue. The comparison was the pre-exercise, baseline, or non-fatigued state. The outcomes were changes in body-fluid biomarkers measured in biological matrices including blood, plasma, serum, saliva, sweat, urine, and interstitial fluid.

Search terms related to exercise, fatigue, and biomarkers were combined using Boolean operators as follows:

(Exercise **OR** Endurance Exercise **OR** Resistance Exercise **OR** High-Intensity Interval Training) **AND** (Fatigue **OR** Fatigability) **AND** (Biomarker **OR** Marker **OR** Indicator **OR** Body Fluid **OR** Blood **OR** Plasma **OR** Serum **OR** Saliva **OR** Sweat **OR** Urine **OR** Metabolism **OR** Metabolome **OR** Metabolic **OR** Metabolomics **OR** Metabolite)

To reduce confounding from pathological fatigue and disease-related conditions, studies focusing on clinical disorders were excluded using the following terms with the **NOT** operator:

(Disease **OR** Cancer **OR** Tumor **OR** Parkinson’s Disease **OR** Multiple Sclerosis **OR** Rheumatoid Arthritis **OR** Multiple Myeloma **OR** Spinal and Bulbar Muscular Atrophy **OR** Metabolic Syndrome **OR** Chronic Obstructive Pulmonary Disease)

The search was restricted to peer-reviewed articles published in English. Reference lists of relevant articles were also screened to identify additional eligible studies.

### 2.3. Eligibility Criteria

Studies were included if they met the following criteria:

(1) The study population consisted of apparently healthy human participants without reported acute or chronic diseases, major injuries, pregnancy, or clinically diagnosed pathological fatigue;
(2) The study investigated acute exercise protocols associated with physical fatigue or substantial exercise stress, with biomarker measurements obtained at pre-exercise and post-exercise time points. When reported, fatigue-related endpoints included increased perceived exertion, volitional exhaustion, task failure, performance decrement, neuromuscular impairment, or other protocol-defined fatigue criteria;
(3) At least one biomarker was measured in a body-fluid sample, including blood, plasma, serum, saliva, sweat, urine, or interstitial fluid;
(4) Biomarker values were reported before and after exercise or before and after exercise protocols intended to induce fatigue;
(5) The article was published in a peer-reviewed journal.

Studies were excluded if they met any of the following criteria:

(1) Participants were pregnant or had major diseases, injuries, or clinically diagnosed pathological fatigue;
(2) The study was conducted in animals or in vitro models only;
(3) Essential quantitative data were unavailable or could not be extracted;
(4) The article was not written in English;
(5) The study was a review, editorial, commentary, conference abstract, case report, or duplicate publication.

For the conventional biomarker meta-analysis, biomarkers were selected for quantitative pooling when they were reported in at least ten independent comparisons with sufficient extractable quantitative data.

For the metabolomics synthesis, studies were included when they reported exercise-related metabolite alterations using metabolomics or targeted metabolite profiling and provided fold-change values, relative abundance changes, or sufficient information to derive post-exercise versus pre-exercise changes. Metabolites were evaluated based on the direction and consistency of reported changes across studies and were interpreted as candidate exercise-responsive metabolites.

### 2.4. Study Selection and Data Extraction

After duplicate records were removed, titles and abstracts were screened for relevance. Potentially eligible articles were then assessed by full-text review. Studies that did not meet the inclusion criteria or lacked extractable quantitative data were excluded. For each eligible study, the following information was extracted: first author, publication year, participant characteristics, sample size, age, sex, body mass index or body fat percentage, training status, fatigue-related criteria or endpoints when reported, exercise modality, exercise protocol, exercise intensity, exercise duration, biofluid type, biomarker name, sampling time point, pre-exercise or baseline value, post-exercise value, measure of variability, and reported statistical results.

Exercise modalities were categorized as endurance exercise, resistance exercise, high-intensity interval training, or mixed exercise. Mixed exercise was defined as a protocol that combined two or more exercise modalities. Biofluid types were categorized as whole blood, plasma, serum, saliva, sweat, urine, or interstitial fluid. Biofluid type was extracted for each included comparison and summarized as a potential source of biological and methodological heterogeneity.

The distribution of biological matrices was summarized using a bar chart. However, because matrix distribution was uneven across biomarkers and many biomarker–matrix combinations contained insufficient comparisons, robust matrix-specific meta-analysis was not feasible for all outcomes. Therefore, pooled estimates were interpreted as overall exercise-responsive effects across available biological matrices, with matrix-related variability considered as a limitation.

### 2.5. Methodological Quality Assessment

Because the included studies predominantly used single-group pre–post exercise designs or other non-randomized exercise intervention designs, the Cochrane risk-of-bias framework was not considered appropriate for the majority of studies. Therefore, methodological quality was assessed using the JBI Critical Appraisal Checklist for Quasi-Experimental Studies, which is suitable for evaluating non-randomized pre–post study designs.

The assessment included domains related to study design appropriateness, participant characteristics, description of exercise protocols, outcome measurement reliability, completeness of follow-up, identification of confounding factors, and appropriateness of statistical analysis. Each item was evaluated as “Yes,” “No,” or “Unclear.” Two reviewers independently performed the methodological quality assessment, and disagreements were resolved through discussion.

### 2.6. Participants for Exploratory LC-MS Pilot Study

Seven apparently healthy young male volunteers were recruited for the exploratory targeted LC-MS pilot study. The age range was 18 to 27 years, and the body mass index range was 19.6 to 28.6 kg·m^−2^. All participants were free from known cardiovascular, metabolic, or musculoskeletal disorders and had not engaged in strenuous exercise within 48 h before testing. Participants were instructed to avoid caffeine intake for at least 24 h before the experiment. They were also asked to rinse their mouths with water 1 h before saliva collection to reduce contamination from food residues and oral metabolites. Participant characteristics are summarized in Table S1.

A formal power calculation was not performed because this experiment was designed as an exploratory within-subject pilot assessment of candidate biomarkers identified from the systematic synthesis and metabolomics analysis, rather than a confirmatory validation study. The sample size was determined based on feasibility, volunteer availability, and the paired pre-exercise versus post-exercise study design. Therefore, LC-MS findings were interpreted as preliminary pilot support and hypothesis-generating evidence requiring further validation in larger and more diverse cohorts.

### 2.7. Exercise-Induced Physical Fatigue Protocol and Sample Collection

Exercise-induced physical fatigue was induced using a motorized treadmill (Lode Valiant 2 CPET Treadmill, Lode BV, Groningen, The Netherlands). A within-subject pre–post design was applied, in which each participant served as his own control. Plasma and saliva samples collected at pre-exercise baseline were compared with paired samples collected post-exercise after reaching volitional exhaustion.

The exercise protocol consisted of four consecutive phases (Figure S1): rest, warm-up, incremental exercise, and recovery. Participants first stood quietly on the treadmill for 1 min to establish baseline conditions. This was followed by a 3 min warm-up at a speed of 3.5 km·h^−1^ with a 1.0% incline. During the incremental exercise phase, treadmill speed and incline were increased simultaneously by 0.5 km·h^−1^ and 0.5% per minute, respectively, to progressively increase exercise intensity. The incremental phase continued until volitional exhaustion, defined as the inability to maintain the required workload despite verbal encouragement. This endpoint was used as the operational criterion for exercise-induced physical fatigue in the pilot experiment.

Immediately after exhaustion, participants completed a 3 min recovery phase at a speed of 3.5 km·h^−1^ and a 1.0% incline. The rating of perceived exertion (RPE) was recorded at pre-exercise and post-exercise time points using a 0–10 scale as a subjective indicator of perceived exertion during the exercise protocol. The average post-exercise RPE score was 8.0 ± 0.8 (Table S1), indicating a high perceived exertion level following the treadmill protocol.

Saliva samples were collected using Salivette Cortisol collection devices (SARSTEDT, Nümbrecht, Germany; LOT NO: 4092221). Blood samples were collected using ethylenediaminetetraacetic acid dipotassium vacuum blood collection tubes (KWS Medical, Shijiazhuang, China; LOT NO: 240923). Both saliva and blood samples were collected at pre-exercise baseline and post-exercise after completion of the treadmill protocol. After collection, samples were centrifuged at 2000× *g* for 5 min at 4 °C to separate saliva supernatant and plasma. The processed samples were stored at −80 °C until LC-MS analysis.

### 2.8. Targeted LC-MS Analysis

A targeted LC-MS method was developed for simultaneous quantification of candidate acute exercise-responsive biomarkers in saliva and plasma. The selected biomarkers included hypoxanthine, xanthine, lactate, pyruvate, isoleucine, phenylalanine, tyrosine, tryptophan, cortisol, and testosterone. These targets were selected based on evidence from the meta-analysis, metabolomics synthesis, and their biological relevance to glycolysis, purine metabolism, amino acid metabolism, and endocrine responses.

Sample preparation involved the addition of caffeine as an internal standard, followed by protein precipitation, centrifugation, drying, and reconstitution. Caffeine was used as a practical internal standard to monitor sample preparation consistency and instrumental response stability. Matrix-matched calibration curves and multi-level quality-control samples were applied to assess analytical performance and reduce matrix-related variability.

To improve analyte separation according to physicochemical properties, both reversed-phase C18 and hydrophilic interaction liquid chromatography columns were used. The method was applied to paired plasma and saliva samples collected at pre-exercise baseline and post-exercise after volitional exhaustion. Detailed LC-MS conditions, calibration procedures, and quality-control information are provided in the Supplementary Materials.

### 2.9. Statistical Analysis

For the conventional biomarker meta-analysis, the effect measure was defined as the post-exercise/pre-exercise response ratio for continuous biomarker concentrations. For each post-exercise/pre-exercise comparison, the effect estimate was calculated on the logarithmic scale as:

$$\log\mathrm{response}\mathrm{ratio}=\ln\;\left(\frac{{\mathrm{Mean}}_{\mathrm{post}}}{{\mathrm{Mean}}_{\mathrm{pre}}}\right)$$

A response ratio greater than 1 indicated a higher post-exercise biomarker concentration, whereas a response ratio less than 1 indicated a lower post-exercise biomarker concentration. Because most included comparisons were paired pre–post observations, the standard error of the log response ratio was calculated by incorporating the within-participant correlation between pre-exercise and post-exercise measurements. When the original studies did not report this correlation coefficient, *r* = 0.5 was assumed for the primary analysis. Pooled log response ratios were calculated using the generic inverse-variance random-effects model and then back-transformed for presentation as response ratios with corresponding 95% confidence intervals. The variance of the log response ratio was calculated as:

$${\mathrm{SE}}_{\log\;\mathrm{response}\;\mathrm{ratio}}=\sqrt{\frac{{\mathrm{SD}}_{\mathrm{post}}^{2}}{n\times {\mathrm{Mean}}_{\mathrm{post}}^{2}}+\frac{{\mathrm{SD}}_{\mathrm{pre}}^{2}}{n\times {\mathrm{Mean}}_{\mathrm{pre}}^{2}}-\frac{2r\times {\mathrm{SD}}_{\mathrm{post}}\times {\mathrm{SD}}_{\mathrm{pre}}}{n\times {\mathrm{Mean}}_{\mathrm{post}}\times {\mathrm{Mean}}_{\mathrm{pre}}}}$$

The random-effects model was selected because of expected heterogeneity across exercise protocols, participant characteristics, sampling matrices, and analytical methods. Subgroup analyses were performed according to exercise modality, including endurance exercise, resistance exercise, high-intensity interval training, and mixed exercise. Between-study heterogeneity was assessed using the I^2^ statistic and Tau^2^. Subgroup differences were evaluated using the Chi^2^ test. Statistical significance was defined as *p* < 0.05. Small-study effects and potential funnel plot asymmetry were assessed using funnel plot inspection, Egger’s regression test, and Begg’s rank correlation test when ≥10 studies were available.

For metabolomics studies, because individual-level quantitative data and variance estimates were not consistently available, a descriptive fold-change-based directional synthesis was performed. Studies were eligible when they reported exercise-related metabolite changes using untargeted metabolomics, targeted metabolomics, or targeted metabolite profiling and provided post-exercise/pre-exercise fold changes, relative abundance changes, or sufficient information to determine the direction of change. Extracted metabolites were categorized into predefined biochemical groups, including carbohydrate metabolites and tricarboxylic acid cycle intermediates, fatty acids and acylcarnitines, amino acids and related metabolites, nucleotides and related metabolites, and vitamins, proteins, cytokines, steroids, hormones, and other molecules. When available, fold changes were transformed into log_2_ fold-change values. Both significant and non-significant changes reported in the original studies were considered; however, metabolites measured but not reported could not be systematically recovered. Therefore, this analysis was interpreted as exploratory and hypothesis-generating rather than a formal quantitative meta-analysis.

For the exploratory targeted LC-MS pilot study, biomarker responses were evaluated using individual concentration changes and log_2_(post-exercise/pre-exercise) fold changes in paired plasma and saliva samples. Given the limited sample size (*n* = 7), this analysis was considered descriptive and hypothesis-generating rather than a confirmatory validation analysis. No formal hypothesis testing or multiple-testing correction was applied, and biomarker responses were interpreted based on the magnitude and direction of change. Directional agreement between plasma and saliva responses was further explored using within-participant correlation analyses.

All meta-analyses and methodological quality assessments were performed using Review Manager (RevMan, version 5.4). Data visualization was conducted using OriginPro 2021 (version 9.8.0.200).

## 3. Results

### 3.1. Meta-Analysis of Acute Exercise-Responsive Biomarkers

**Figure 1:**
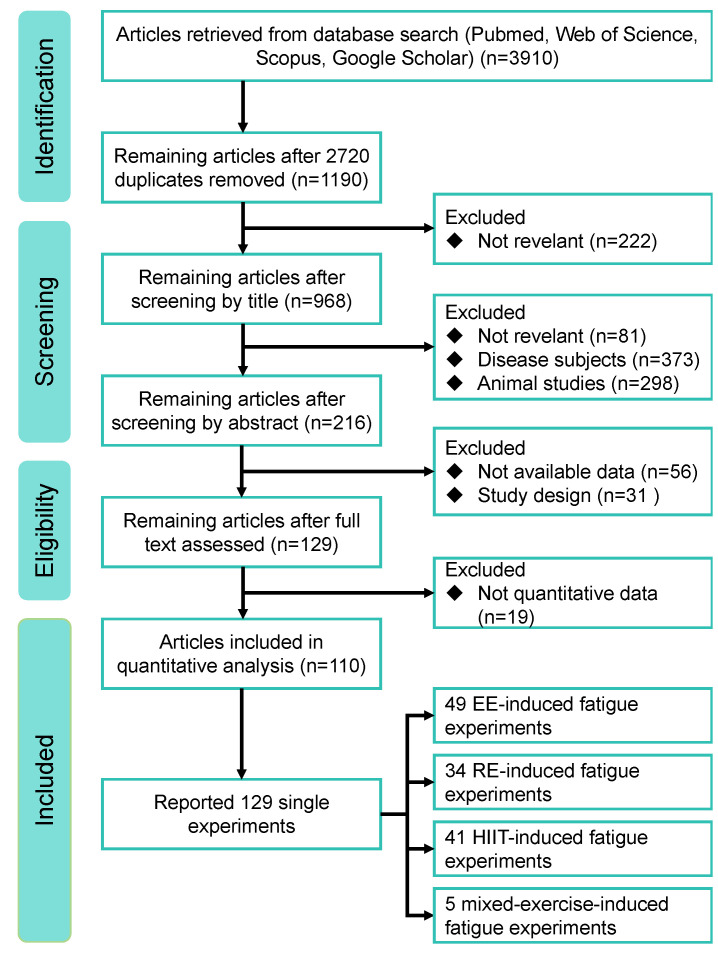
PRISMA flowchart from the systematic literature search.

A total of 3910 records were identified through the literature search. After duplicate removal and screening of titles, abstracts, and full texts, records were excluded because they were irrelevant to the research question, disease-related, animal studies, lacked accessible data, or did not provide extractable quantitative data. Ultimately, 110 articles were included in the conventional biomarker synthesis and meta-analysis. Because some articles reported multiple exercise protocols or exercise modalities, these 110 articles contributed 129 single experiments/comparisons for quantitative synthesis (Figure 1 and Table S2).

The included studies covered diverse exercise modalities, protocols, and biological matrices. Exercise protocols were designed to induce physical fatigue or substantial exercise stress, while fatigue-related endpoints were not consistently reported across studies. Some studies included perceived exertion, volitional exhaustion, task failure, performance decrement, or neuromuscular assessments, whereas others reported only pre-exercise and post-exercise biomarker changes following exercise protocols. Therefore, pooled biochemical responses were interpreted as acute exercise-responsive patterns associated with physical fatigue rather than definitive fatigue-specific effects.

Biomarker changes were synthesized using pre-exercise and post-exercise measurements, and quantitative meta-analyses were performed for biomarkers with sufficient extractable data.

#### 3.1.1. Participants and Intervention Characteristics

A total of 1826 apparently healthy adults were included in the eligible studies. The median age was 23.2 years (IQR 20.9–27.7), and the median BMI was 23.2 kg·m^−2^ (IQR 21.8–25.4). Overall, 1560 participants were male and 266 were female. Across the 129 individual experiments, 1434, 331, and 61 participants were classified as trained, untrained, and unclear, respectively (Table S2). Regarding exercise modalities, endurance exercise (EE), resistance exercise (RE), high-intensity interval training (HIIT), and mixed exercise (referred to any combination of two or three modalities among EE, RE, and HIIT) were used in 49, 34, 41, and 5 interventions, respectively (Figure S2). In terms of biospecimens, biomarkers were quantified in blood, saliva, urine, and interstitial fluid (ISF) samples in 117, 8, 3, and 1 experiments, respectively (Figure S2).

Regarding biological matrices, biomarkers were quantified in whole blood, plasma, serum, saliva, sweat, urine, and interstitial fluid samples across the included experiments. Blood-related samples represented the majority of measurements, whereas saliva, urine, sweat, and interstitial fluid were less frequently reported (Figure S2). The diversity of exercise protocols, participant characteristics, and biological matrices represented important sources of methodological heterogeneity across studies.

For EE, the included studies covered soccer matches, cycling, running, judo matches, marathon events, 1500 m freestyle swimming, and speed skating competitions. The median duration of EE protocols was 60 min (IQR 30–210 min). For RE, interventions included timed push-ups, leg press, bench press, muscle strength training, and resisted cycling, with a median duration of 24 min (IQR 6–30 min). HIIT protocols comprised multi-event sport, kickboxing, battle rope exercise, rugby training, shuttle running with speed changes, and the Loughborough Intermittent Shuttle Test. MIX protocols included activities such as running, cycling, and reverse sit-ups.

After full-text review of 110 articles, a total of 121 distinct biomarkers were identified and quantitatively reported. Across all included articles and extractable comparisons, these biomarkers generated 522 biomarker-reporting occurrences. Therefore, the percentages below represent the proportion of each biomarker among all biomarker-reporting occurrences, rather than the proportion of articles. To ensure comparability and robustness, we selected nine biomarkers that were reported at least 10 times across the literature: lactate (70 occurrences, 13.4%), creatine kinase (CK) (59 occurrences, 11.3%), cortisol (40 occurrences, 7.6%), testosterone (25 occurrences, 4.8%), urea (21 occurrences, 4.0%), glucose (18 occurrences, 3.4%), C-reactive protein (CRP) (15 occurrences, 2.9%), lactate dehydrogenase (LDH) (11 occurrences, 2.1%), and interleukin 6 (IL-6) (10 occurrences, 1.9%) (Table S3).

In the following subsections, we describe changes in these biomarkers at pre-exercise and post-exercise time points following EE, RE, HIIT, or MIX protocols. The number of studies and effect sizes within each exercise modality may differ from the overall pooled estimates because modality-specific meta-analyses were conducted only when sufficient extractable data were available.

#### 3.1.2. Conventional Biomarker Responses to Acute Exercise-Induced Physical Fatigue

Among the evaluated conventional biomarkers, lactate, CK, LDH, and IL-6 showed significant post-exercise increases and were selected for detailed meta-analysis because they were reported with sufficient frequency and demonstrated statistically significant pooled responses across the included comparisons. Pooled effects were calculated using an inverse-variance random-effects model with log response ratios as effect estimates and were back-transformed to response ratios for presentation.

##### Lactate

**Figure 2:**
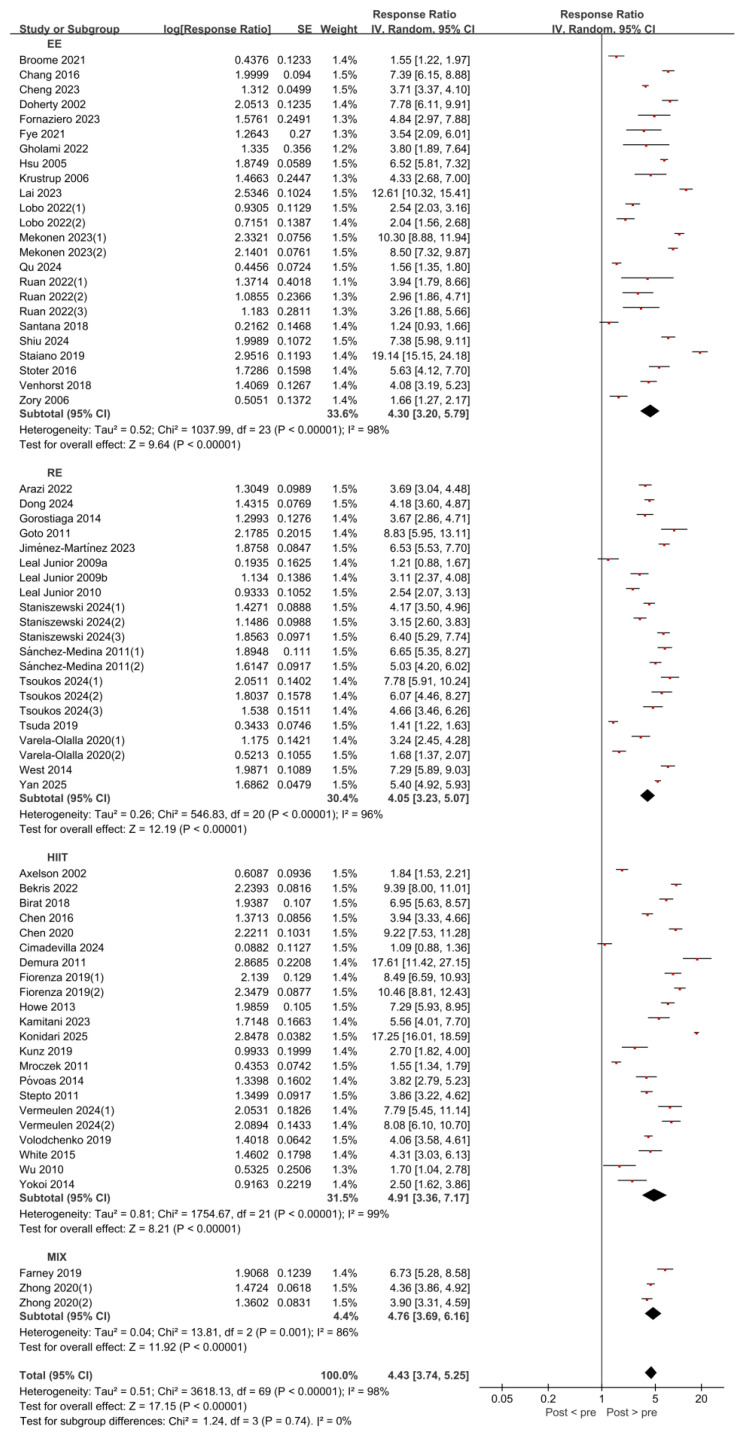
Forest plot of post-exercise/pre-exercise response ratios for lactate concentration. 95% CI, 95% confidence interval; Chi^2^, Chi-squared test; df, Degrees of freedom; I^2^, heterogeneity test; EE, endurance exercise; RE, resistance exercise; HIIT, high intensity interval training; MIX, exercise mixing two or three of EE, RE, and HIIT [32,33,34,35,36,37,38,39,40,41,42,43,44,45,46,47,48,49,50,51,52,53,54,55,56,57,58,59,60,61,62,63,64,65,66,67,68,69,70,71,72,73,74,75,76,77,78,79,80,81,82,83,84,85].

For lactate, 70 within-subject comparisons (865 participants in total) were included. Lactate levels were significantly higher at post-exercise compared with pre-exercise (response ratio = 4.43, 95% CI [3.74, 5.25], Z = 17.15, *p* < 0.00001) (Figure 2). However, the 95% prediction interval ranged from 1.09 to 18.02 (Table S4), indicating substantial uncertainty in the magnitude of response across future studies. Although substantial heterogeneity was present (Tau^2^ = 0.51; I^2^ = 98%), all exercise-modality subgroups (EE, RE, HIIT, and MIX) showed a consistent direction of increase, and no significant between-subgroup difference was detected (Chi^2^ = 1.24, *p* = 0.74) (Figure 2), suggesting consistent lactate responsiveness across different exercise modalities while indicating that the magnitude of change may vary substantially among populations and protocols. Visual inspection of the funnel plot showed that most studies were distributed within the 95% confidence limits and clustered around the center line, with no clear pattern of one-sided “missing” studies; however, some dispersion and mild asymmetry were observed in the lower part of the funnel, suggesting that small-study effects and the high heterogeneity may have jointly influenced the plot shape (Figure S3A). Egger’s regression test indicated potential funnel plot asymmetry (*p* = 0.043), whereas Begg’s rank correlation test was not significant (*p* = 0.236) (Table S5). These findings should be interpreted cautiously given the substantial heterogeneity (I^2^ = 98%).

##### CK

**Figure 3:**
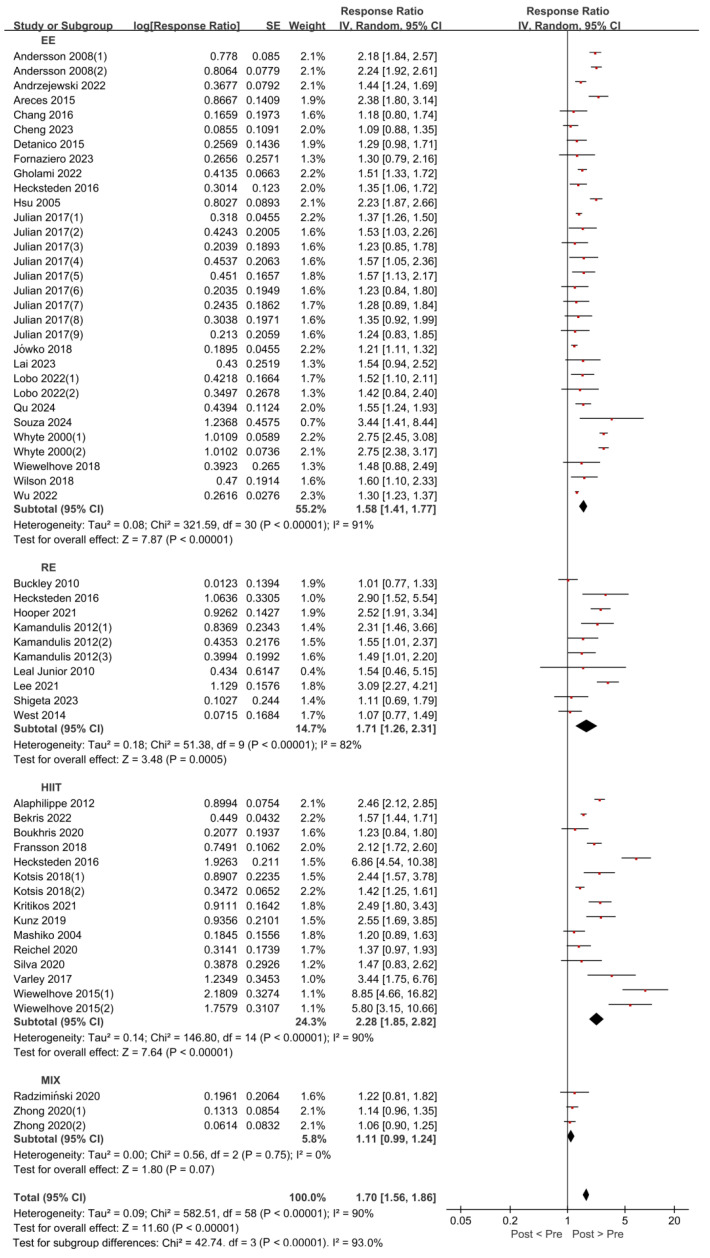
Forest plot of post-exercise/pre-exercise response ratios for CK concentration. 95% CI, 95% confidence interval; Chi2, Chi-squared test; df, Degrees of freedom; I2, heterogeneity test; EE, endurance exercise; RE, resistance exercise; HIIT, high intensity interval training; MIX, exercise mixing two or three of EE, RE, and HIIT [20,33,34,36,38,39,41,42,44,58,63,66,75,85,86,87,88,89,90,91,92,93,94,95,96,97,98,99,100,101,102,103,104,105,106,107,108,109,110,111].

For CK, 59 within-subject comparisons (837 participants in total) were included. The pooled analysis showed that CK levels were significantly higher at post-exercise compared with pre-exercise (response ratio = 1.70, 95% CI [1.56, 1.86], Z = 11.60, *p* < 0.00001; Tau^2^ = 0.09; I^2^ = 90%) (Figure 3), with a wide 95% prediction interval (0.93–3.12) (Table S4), indicating considerable uncertainty in the magnitude of CK response across future studies. In subgroup analyses, EE, RE, and HIIT all showed significant increases, with a larger pooled effect observed in HIIT, whereas the MIX subgroup did not reach statistical significance. The test for subgroup differences suggested that exercise modality may influence the magnitude of CK response (Chi^2^ = 42.74, *p* < 0.00001) (Figure 3), indicating that exercise characteristics may contribute to variability in CK responses across studies. The CK funnel plot showed that most studies were distributed within the 95% confidence limits and clustered around the center line, without an obvious one-sided missing pattern (Figure S3B). However, some dispersion and subgroup clustering were observed. Egger’s regression test indicated potential funnel plot asymmetry (*p* = 0.0499), whereas Begg’s rank correlation test was not significant (*p* = 0.1540) (Table S5). Therefore, potential small-study effects and funnel plot asymmetry should be interpreted cautiously considering the substantial heterogeneity among studies.

##### LDH

**Figure 4:**
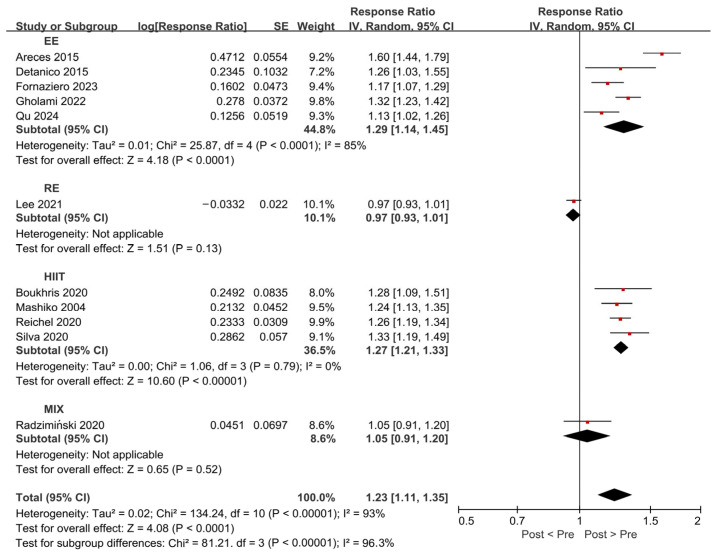
Forest plot of post-exercise/pre-exercise response ratios for LDH concentration. 95% CI, 95% confidence interval; Chi^2^, Chi-squared test; df, Degrees of freedom; I^2^, heterogeneity test; EE, endurance exercise; RE, resistance exercise; HIIT, high intensity interval training; MIX, exercise mixing two or three of EE, RE, and HIIT [36,38,44,88,89,100,103,106,107,108,110].

For LDH, 11 within-subject comparisons (239 participants in total) were included. The pooled analysis showed that LDH levels were significantly higher at post-exercise compared with pre-exercise (Response ratio = 1.23 95% CI [1.11, 1.35], Z = 4.08, *p* < 0.0001; Tau^2^ = 0.02; I^2^ = 93%) (Figure 4), with a 95% prediction interval of 0.89–1.69 (Table S4). In subgroup analyses, both EE and HIIT showed significant increases, and the HIIT subgroup demonstrated good internal consistency (I^2^ = 0%), suggesting that LDH may complement lactate by reflecting changes related to glycolytic metabolic stress and/or alterations in muscle cell membrane permeability. In the LDH funnel plot, study points were largely distributed within the 95% confidence limits and were broadly scattered around the center line; however, given the small number of included studies and the substantial overall heterogeneity, the funnel plot shape may have been influenced by both small-study effects and between-study differences (Figure S3C). Neither Egger’s regression test (*p* = 0.0800) nor Begg’s rank correlation test (*p* = 0.6481) indicated significant funnel plot asymmetry (Table S5).

##### IL-6

**Figure 5:**
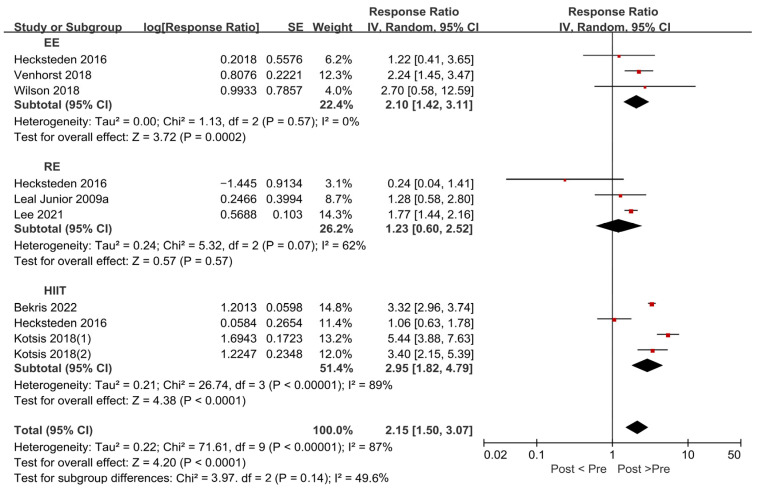
Forest plot of post-exercise/pre-exercise response ratios for IL-6 concentration. 95% CI, 95% confidence interval; Chi^2^, Chi-squared test; df, Degrees of freedom; I^2^, heterogeneity test; EE, endurance exercise; RE, resistance exercise; HIIT, high intensity interval training; MIX, exercise mixing two or three of EE, RE, and HIIT [56,66,90,95,100,105,112].

For the inflammatory marker IL-6, 10 within-subject comparisons (183 participants in total) were included. The pooled analysis showed that IL-6 levels were significantly higher at post-exercise compared with pre-exercise (Response ratio = 2.15, 95% CI [1.50, 3.07], Z = 4.20, *p* < 0.0001; Tau^2^ = 0.22; I^2^ = 87%) (Figure 5), with a wide 95% prediction interval (0.80–5.76) (Table S4). Subgroup analyses indicated significant increases in both EE and HIIT, suggesting that acute exercise-induced fatigue is accompanied not only by metabolic stress and muscle-damage burden but also by activation of inflammatory/immune responses Overall, lactate, CK, LDH, and IL-6 exhibited clearer statistical differences and relatively consistent directional changes before versus after fatigue. Together, they reflect exercise-induced fatigue from three complementary dimensions—metabolic load, muscle damage/membrane stability, and inflammatory responses—supporting their prioritization as a candidate biomarker panel for physical fatigue, with potential utility for future multi-marker classification and threshold validation studies. In the IL-6 funnel plot, study points were generally distributed within the 95% confidence limits and showed some scatter around the center line. Given the limited number of included studies and the presence of heterogeneity, the funnel plot shape may have been influenced by both small-study effects and between-study differences (Figure S3D). Neither Egger’s regression test (*p* = 0.1944) nor Begg’s rank correlation test (*p* = 0.1083) indicated significant funnel plot asymmetry (Table S5).

##### Other Exercise-Responsive Biomarker Changes

Additional meta-analyses were conducted for cortisol, testosterone, urea, glucose, and CRP. Compared with lactate, CK, LDH, and IL-6, these biomarkers showed more heterogeneous and less consistent post-exercise responses across studies. Cortisol (Figure S4, [20,33,36,45,51,54,61,63,66,82,85,87,90,91,101,107,113,114,115,116,117,118,119,120,121,122]) and urea (Figure S6, [20,36,43,55,67,86,90,111]) demonstrated modest overall increases, whereas testosterone (Figure S5, [20,51,54,56,63,66,82,90,91,115,117,118,119,123]) showed a larger but highly heterogeneous response. Glucose (Figure S7, [33,35,36,40,43,61,67,71,77,78,100,106,116,122,124,125]) and CRP (Figure S8, [45,58,90,95,103,104,105,110,111]) did not show statistically significant overall changes. Given the substantial heterogeneity and variability across exercise protocols, participant characteristics, and biological matrices, these biomarkers were interpreted as context-dependent exercise-responsive changes rather than universal indicators of physical fatigue. Funnel plots were visually inspected for these outcomes, and formal Egger’s regression tests and Begg’s rank correlation tests were performed when the number of included comparisons was sufficient. No significant evidence of funnel plot asymmetry was detected for cortisol, testosterone, urea, glucose, or CRP (Table S5), although the interpretation of publication bias remained limited by substantial heterogeneity and the relatively small number of studies for some outcomes.

#### 3.1.3. Methodological Quality of the Included Studies

**Figure 6:**
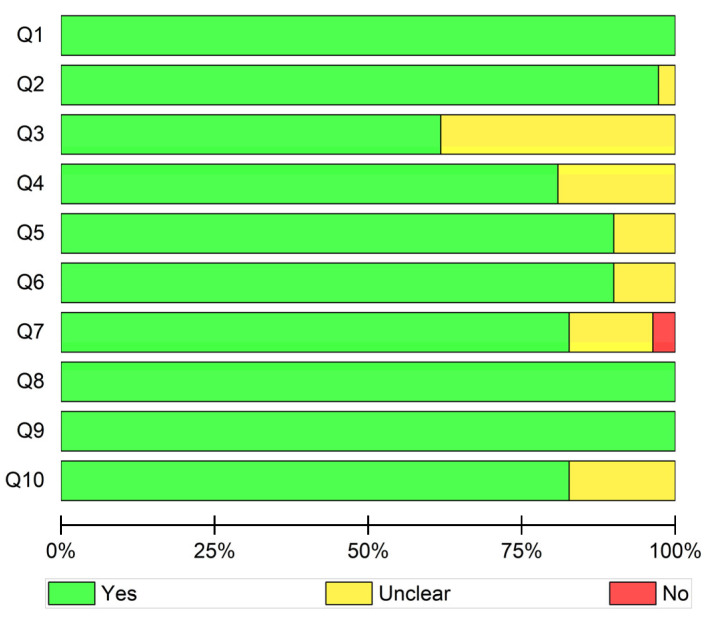
Methodological quality assessment summary of included studies using the adapted Joanna Briggs Institute (JBI) Critical Appraisal Checklist for Quasi-Experimental Studies. Q1, Clear cause-effect relationship; Q2, Participants described; Q3, Exercise protocol clearly described; Q4, Fatigue/exercise endpoint defined; Q5, Pre-post measurements performed; Q6, Follow-up/outcome completeness; Q7, Confounders/pre-analytical factors considered; Q8, Outcome measured consistently; Q9, Biomarker measurement reliable; Q10, Statistical analysis appropriate.

The methodological quality of the included studies was assessed using an adapted Joanna Briggs Institute (JBI) Critical Appraisal Checklist for Quasi-Experimental Studies (Figure 6 and Table S6). Since most included studies adopted single-group pre–post exercise designs, the assessment focused on study design clarity, participant description, exercise protocol reporting, fatigue/exercise endpoint definition, pre–post measurement completeness, outcome measurement reliability, consideration of confounding factors, and statistical analysis appropriateness.

Overall, the included studies showed generally acceptable methodological quality. All studies clearly described the cause–effect relationship between exercise exposure and biomarker changes (Q1: 100%), and most studies adequately reported participant characteristics (Q2: 97.27%) and biomarker measurement procedures (Q9: 100%). Pre–post biomarker measurements and outcome completeness were also generally well reported (Q5 and Q6: 90.00% Yes).

However, some methodological limitations remained. Exercise protocols were insufficiently described in a proportion of studies (Q3: 61.82% Yes, 38.18% Unclear), and fatigue/exercise endpoints were not consistently defined across studies (Q4: 80.91% Yes, 19.09% Unclear). Although most studies considered potential confounding factors or pre-analytical conditions (Q7: 82.73% Yes), residual uncertainties remained due to incomplete reporting of factors such as nutritional status, hydration, sampling conditions, and training status. Statistical analyses were considered appropriate in most studies (Q10: 82.73% Yes), while 17.27% remained unclear due to insufficient reporting of statistical methods.

Taken together, the methodological quality assessment indicated that the included studies generally provided reliable evidence regarding acute exercise-associated biomarker responses; however, variability in fatigue endpoint definition, exercise protocol standardization, confounder control, and statistical reporting may contribute to between-study heterogeneity. Therefore, the meta-analytic findings should be interpreted considering these methodological limitations.

Although conventional exercise-responsive biomarkers, including lactate, CK, LDH, and IL-6, showed relatively consistent post-exercise changes across studies, these markers are well-established indicators of metabolic stress, muscle damage-related responses, and inflammatory processes. Therefore, conventional biomarker meta-analysis mainly captured reproducible physiological responses rather than providing extensive insight into novel metabolic candidates. To further explore potential candidate biomarkers and improve mechanistic interpretation, we next synthesized exercise-related metabolomics studies focusing on broader metabolic alterations associated with acute exercise.

### 3.2. Identification of Candidate Biomarkers from Metabolomic Studies

For the metabolomics synthesis, 935 records were identified. After duplicate removal and eligibility screening, 28 articles [5,20,90,94,126,127,128,129,130,131,132,133,134,135,136,137,138,139,140,141,142,143,144,145,146,147,148,149] were included. Because several articles reported multiple exercise protocols, sampling conditions, or metabolomics comparisons, these 28 articles contributed 35 independent experiments/comparisons to the fold-change directional synthesis (Figure S9 and Table S7).

A total of 619 apparently healthy adults were included in the eligible metabolomics studies. The median age was 24.7 years (IQR 20.5–34.14), and the median BMI was 23.0 kg·m^−2^ (IQR 22.1–24.0). Among participants, 502 were male and 117 were female (Table S7). Across 35 individual experiments, single-modality exercise protocols included 23 EE, 5 RE, and 7 HIIT experiments (Figure S10). EE protocols included cycling, running, rowing, and marathon events. RE protocols mainly involved leg press and resisted cycling, whereas HIIT protocols primarily consisted of interval running and cycling. Regarding biological matrices, metabolomics analyses were performed using blood, sweat, saliva, and urine samples in 23, 2, 2, and 8 experiments, respectively (Figure S10). In the following subsections, we describe exercise-associated changes in these metabolite categories following single bouts of EE, RE, or HIIT.

A total of 542 post-exercise/pre-exercise fold-change records were extracted and categorized into predefined biochemical groups, including carbohydrate metabolites and tricarboxylic acid cycle intermediates, fatty acids and acylcarnitines, amino acids and related metabolites, nucleotides and related metabolites, and vitamins, proteins, cytokines, steroids, hormones, and other molecules. Because individual-level quantitative data and variance estimates were not consistently available across metabolomics studies, these findings were synthesized descriptively based on the direction and magnitude of reported changes. The reported metabolite changes were interpreted as acute exercise-responsive metabolic alterations rather than fatigue-specific biomarker effects.

#### 3.2.1. Carbohydrate Metabolites and TCA Cycle

**Figure 7:**
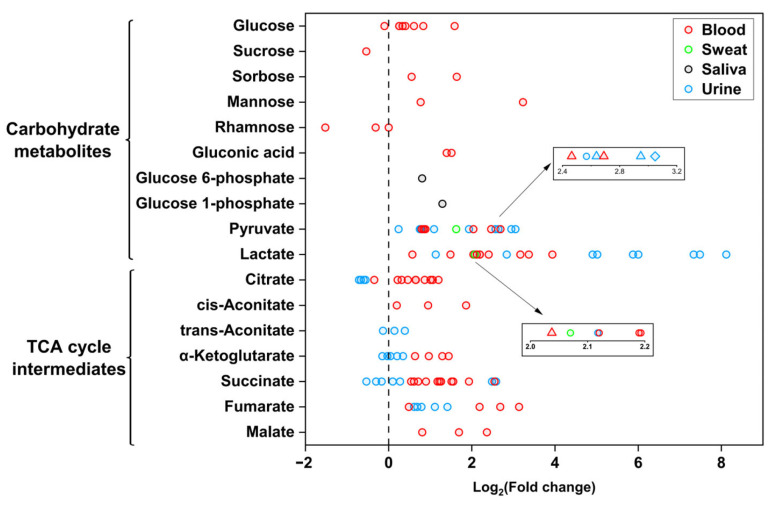
Exercise-associated changes in carbohydrate metabolites and tricarboxylic acid (TCA) cycle intermediates identified from metabolomics studies. Metabolite changes are presented as log_2_(post-exercise/pre-exercise) fold changes. Each point represents a reported metabolite change from an individual comparison. Positive and negative values indicate higher and lower post-exercise metabolite levels, respectively. EE, endurance exercise; RE, resistance exercise; HIIT, high-intensity interval training.

Figure 7 summarizes post-exercise/pre-exercise fold-change patterns for carbohydrate metabolites and tricarboxylic acid (TCA) cycle intermediates. Lactate, pyruvate, and fumarate showed relatively consistent positive log_2_ fold-change patterns across studies, with lactate and pyruvate being repeatedly reported in multiple biological matrices. In contrast, glucose and several TCA cycle intermediates, including citrate, α-ketoglutarate, and succinate, showed more dispersed fold-change distributions, with positive, near-zero, and negative changes coexisting across studies.

When stratified by biological matrix, positive lactate and pyruvate responses were most frequently observed in blood-related samples, whereas urine showed larger dispersion in fold-change magnitude, likely reflecting differences in metabolite accumulation, excretion, and sampling time. Limited sweat data also showed positive signals for lactate and pyruvate, suggesting potential relevance for non-invasive monitoring, although the small number of sweat-based comparisons precludes firm conclusions. Across exercise modalities, lactate and pyruvate tended to increase after EE, RE, and HIIT, with larger changes generally observed after higher-intensity protocols.

#### 3.2.2. Fatty Acids and Acylcarnitine

**Figure 8:**
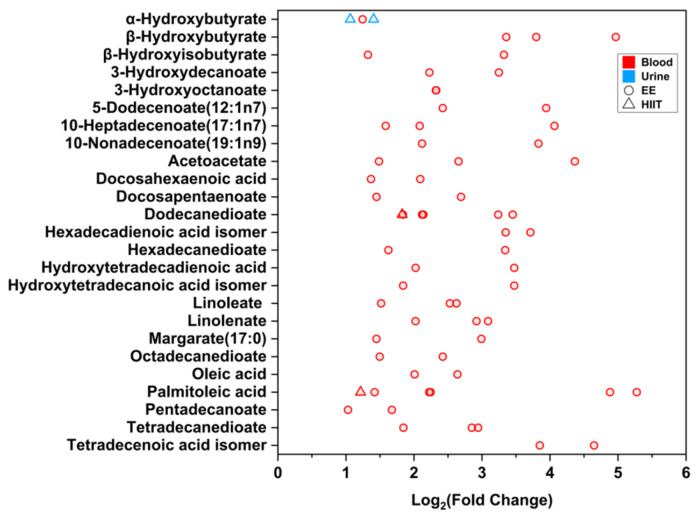
Exercise-associated changes in fatty acids identified from metabolomics studies. Metabolite changes are presented as log_2_(post-exercise/pre-exercise) fold changes. Each point represents a reported metabolite change from an individual comparison. Positive and negative values indicate higher and lower post-exercise metabolite levels, respectively. EE, endurance exercise; RE, resistance exercise; HIIT, high-intensity interval training.

Figure 8 summarizes the post-exercise/pre-exercise fold-change patterns of fatty acids and related lipid metabolites. Fatty acids with log_2_ fold changes greater than 1 reported in at least two independent studies were included for directional synthesis. Most reported fatty acids showed positive fold changes after exercise, particularly in endurance exercise protocols, although the magnitude of responses varied across studies and biological matrices.

**Figure 9:**
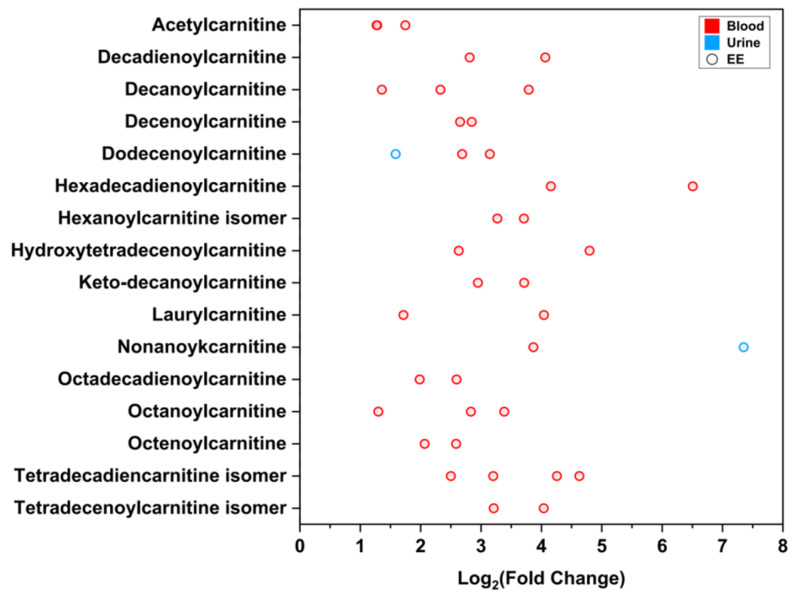
Exercise-associated changes in acylcarnitines identified from metabolomics studies. Metabolite changes are presented as log_2_(post-exercise/pre-exercise) fold changes. Each point represents a reported metabolite change from an individual comparison. Positive and negative values indicate higher and lower post-exercise metabolite levels, respectively. EE, endurance exercise; RE, resistance exercise; HIIT, high-intensity interval training.

Acylcarnitines showed a similar exercise-responsive pattern, with increased levels mainly reported in blood and urine samples following endurance-based protocols (Figure 9). These findings suggest alterations in lipid mobilization and mitochondrial substrate utilization during acute exercise. However, because the available metabolomics evidence was based on reported fold changes and heterogeneous exercise conditions, these lipid metabolites should be interpreted as candidate exercise-responsive metabolic alterations rather than fatigue-specific biomarkers.

#### 3.2.3. Amino Acids and Related Metabolites

**Figure 10:**
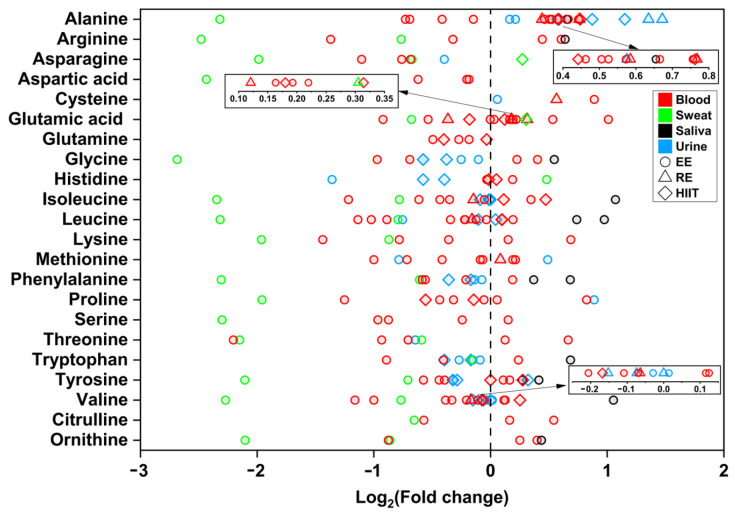
Exercise-associated changes in amino acids identified from metabolomics studies. Metabolite changes are presented as log_2_(post-exercise/pre-exercise) fold changes. Each point represents a reported metabolite change from an individual comparison. Positive and negative values indicate higher and lower post-exercise metabolite levels, respectively. EE, endurance exercise; RE, resistance exercise; HIIT, high-intensity interval training.

**Figure 11:**
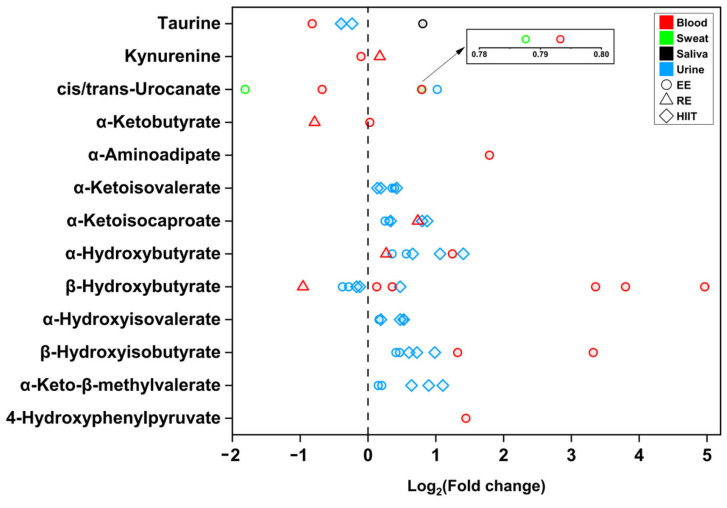
Exercise-associated changes in amino acids related metabolites identified from metabolomics studies. Metabolite changes are presented as log_2_(post-exercise/pre-exercise) fold changes. Each point represents a reported metabolite change from an individual comparison. Positive and negative values indicate higher and lower post-exercise metabolite levels, respectively. EE, endurance exercise; RE, resistance exercise; HIIT, high-intensity interval training.

Figure 10 summarizes exercise-associated changes in amino acids and related metabolites. Compared with lactate and pyruvate, amino acid responses showed greater variability across studies, biological matrices, and exercise modalities, with both positive and negative log_2_ fold changes observed. Some salivary amino acids showed positive responses, whereas sweat-based measurements generally showed smaller or negative changes; however, limited sample availability restricted matrix-specific interpretation. Figure 11 shows changes in amino acid-derived metabolites, including α-ketoisovalerate, α-ketoisocaproate, and hydroxybutyrate derivatives. Although several metabolites showed positive responses after exercise, most were reported in a limited number of studies and should be considered candidate exercise-responsive metabolites requiring further validation.

#### 3.2.4. Nucleotides and Related Metabolites

**Figure 12:**
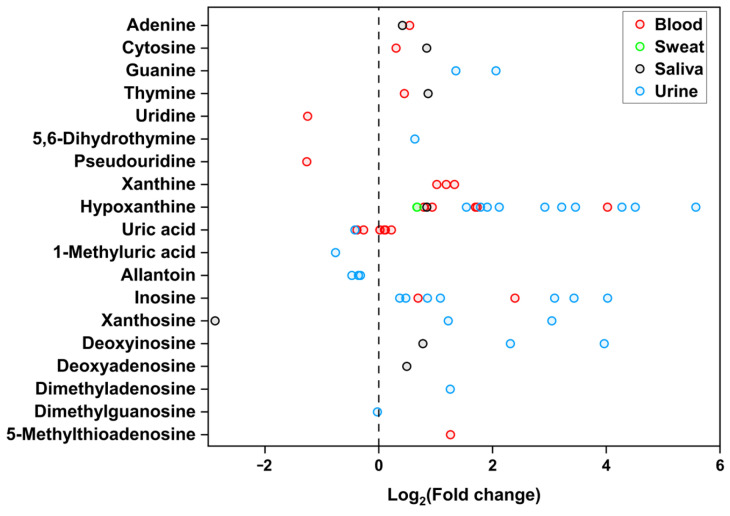
Exercise-associated changes in nucleotides and related metabolites identified from metabolomics studies. Metabolite changes are presented as log_2_(post-exercise/pre-exercise) fold changes. Each point represents a reported metabolite change from an individual comparison. Positive and negative values indicate higher and lower post-exercise metabolite levels, respectively. EE, endurance exercise; RE, resistance exercise; HIIT, high-intensity interval training.

Figure 12 summarizes post-exercise/pre-exercise fold-change patterns of nucleotides and related metabolites. Compared with other metabolite categories, purine-related metabolites showed relatively consistent positive responses across multiple studies and biological matrices. In particular, hypoxanthine, xanthine, inosine, xanthosine, and related purine metabolites were frequently reported with positive log_2_ fold changes, especially in blood and urine samples.

Hypoxanthine showed the most consistent increase among the evaluated nucleotide-related metabolites, with positive responses observed across different exercise modalities and sample types. Urinary hypoxanthine demonstrated relatively pronounced increases, particularly in HIIT and resistance exercise protocols; however, the number of available comparisons was limited, and these findings should be interpreted as candidate exercise-responsive metabolic alterations rather than fatigue-specific biomarkers.

#### 3.2.5. Vitamin, Protein, Cytokine, Steroid, Hormone and Other Molecules

**Figure 13:**
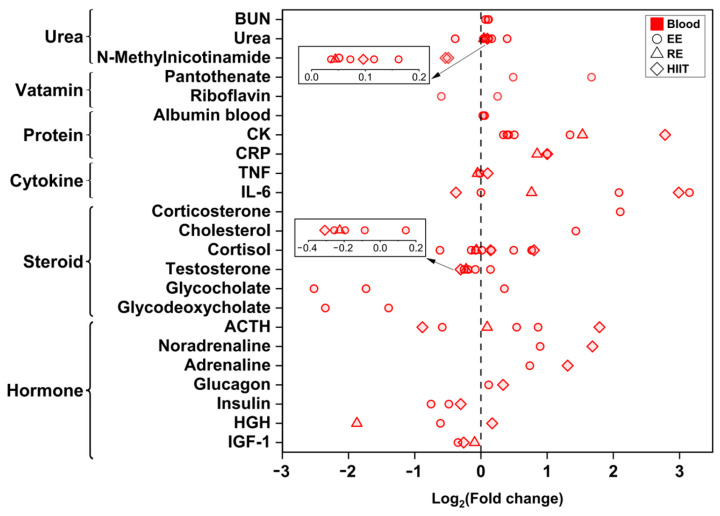
Exercise-associated changes in fatty acids identified from metabolomics studies. Metabolite changes are presented as log_2_(post-exercise/pre-exercise) fold changes. Each point represents a reported metabolite change from an individual comparison. Positive and negative values indicate higher and lower post-exercise metabolite levels, respectively. EE, endurance exercise; RE, resistance exercise; HIIT, high-intensity interval training. BUN, blood urea nitrogen; CK, creatine kinase; CRP, C-reactive protein; TNF, tumor necrosis factor; IL-6, interleukin-6; ACTH, adrenocorticotropic hormone; HGH, human growth hormone; IGF-1, insulin-like growth factor 1.

Figure 13 summarizes post-exercise/pre-exercise fold-change patterns of vitamins, proteins, cytokines, steroids, hormones, and other molecules. Compared with metabolites involved in glycolysis, purine metabolism, and amino acid metabolism, these molecular categories showed greater heterogeneity across studies. Several molecules, including pantothenate, CK, CRP, corticosterone, cholesterol, noradrenaline, adrenaline, and glucagon, showed positive fold changes in some studies, whereas glycocholate and glycodeoxycholate showed negative changes in selected comparisons.

However, the magnitude and direction of responses varied substantially across exercise protocols, biological matrices, and analytical conditions. Because many of these molecules were reported in limited comparisons and exhibited inconsistent patterns, they were interpreted as exercise-responsive molecular alterations rather than robust candidate biomarkers. Further validation using standardized exercise protocols and targeted quantitative approaches is required.

### 3.3. Exploratory LC-MS Assessment of Candidate Exercise-Responsive Metabolites in Paired Plasma and Saliva Samples

**Figure 14:**
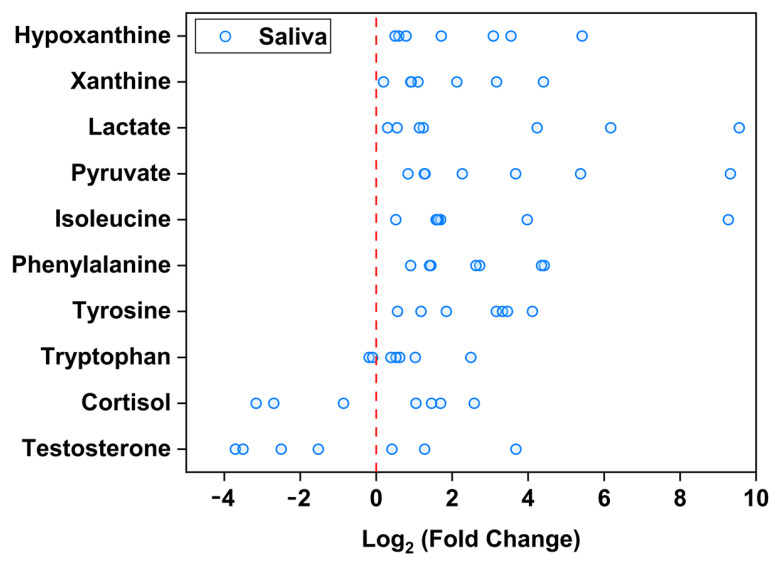
Exploratory LC-MS analysis of candidate acute exercise-responsive biomarkers in saliva. Data are presented as log_2_(post-exercise/pre-exercise) fold changes from seven apparently healthy young male volunteers. Individual biomarker concentrations at pre-exercise and post-exercise time points (mean ± SD) are provided in Table S12.

**Figure 15:**
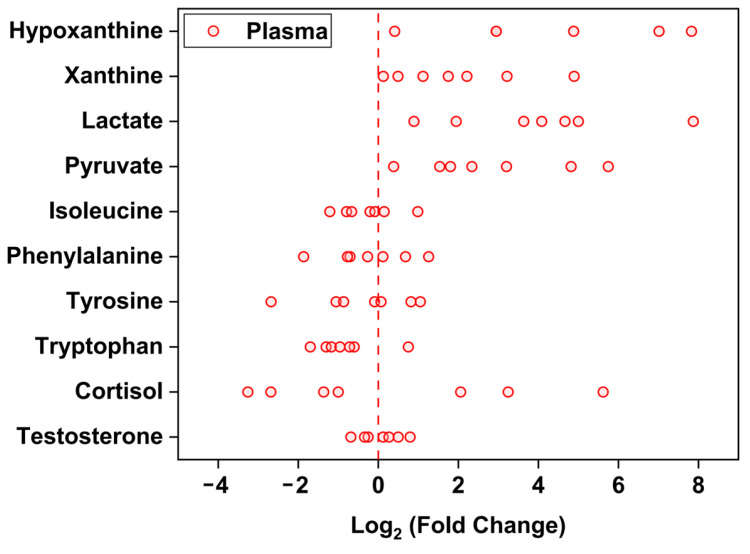
Exploratory LC-MS analysis of candidate acute exercise-responsive biomarkers in plasma. Data are presented as log_2_(post-exercise/pre-exercise) fold changes from seven apparently healthy young male volunteers. Individual biomarker concentrations at pre-exercise and post-exercise time points (mean ± SD) are provided in Table S12.

Based on findings from the meta-analysis and metabolomics synthesis, ten candidate exercise-responsive metabolites and biomarkers, including hypoxanthine, xanthine, lactate, pyruvate, isoleucine, phenylalanine, tyrosine, tryptophan, cortisol, and testosterone, were quantitatively assessed using targeted LC-MS in paired plasma and saliva samples collected from seven apparently healthy young male volunteers. The post-exercise/pre-exercise ratios were calculated and log_2_-transformed to characterize the direction and magnitude of individual metabolite changes (Figure 14 and Figure 15 and Table S12).

In saliva, hypoxanthine, xanthine, lactate, pyruvate, isoleucine, phenylalanine, and tyrosine generally showed positive post-exercise changes, whereas tryptophan, cortisol, and testosterone exhibited variable responses among participants (Figure 14). Similarly, in plasma, hypoxanthine, xanthine, lactate, and pyruvate showed predominantly positive responses, while amino acids and hormone-related markers demonstrated greater inter-individual variability (Figure 15).

To further evaluate the relationship between plasma and salivary responses, Spearman correlation analyses were performed between plasma and saliva log_2_(post-exercise/pre-exercise) fold changes (Table S12). Several metabolites, including lactate and pyruvate, showed positive correlation trends between plasma and saliva responses; however, most associations did not reach statistical significance, likely due to the limited sample size (n = 7) (Table S13). These findings provide preliminary pilot support for several exercise-responsive metabolites but should be interpreted as exploratory evidence requiring validation in larger, matrix-specific cohorts.

## 4. Discussion

In the present study, we integrated systematic review and random-effects meta-analysis, exercise-related metabolomics synthesis, and exploratory targeted LC-MS analysis to identify candidate acute exercise-responsive metabolic biomarkers with potential relevance to physical fatigue assessment. Conventional biomarker analyses identified reproducible post-exercise responses in lactate, CK, LDH, and IL-6, whereas metabolomics synthesis highlighted additional candidate metabolites, particularly hypoxanthine, xanthine, lactate, and pyruvate, associated with glycolysis and purine metabolism. Exploratory LC-MS analysis in paired plasma and saliva samples further provided preliminary human-level support for these metabolic responses. However, because post-exercise biochemical alterations are not necessarily specific to fatigue states, the identified metabolites should be interpreted as candidate acute exercise-responsive biomarkers associated with physical fatigue rather than definitive fatigue-specific biomarkers.

The conventional meta-analysis demonstrated that several established exercise-responsive biomarkers, including lactate, CK, LDH, and IL-6, showed consistent directional increases after acute exercise protocols (Figure 2, Figure 3, Figure 4 and Figure 5). These findings are consistent with classical physiological responses involving enhanced glycolytic activity, muscle membrane perturbation or damage-related processes, and activation of inflammatory signaling pathways [27]. However, substantial heterogeneity was observed across several outcomes (Figure 2, Figure 3, Figure 4 and Figure 5), indicating that the magnitude of these responses may vary depending on exercise characteristics, participant populations, sampling conditions, and analytical approaches. Therefore, these biomarkers should be interpreted as indicators of acute exercise-associated physiological stress rather than universal markers specifically reflecting fatigue severity. In contrast, cortisol, testosterone, urea, glucose, and CRP showed weaker, more heterogeneous, or context-dependent responses in the additional meta-analyses. Although these markers may provide useful auxiliary information regarding endocrine stress, protein metabolism, substrate availability, or systemic inflammation, their responses are strongly influenced by exercise modality, intensity, sampling timing, circadian rhythm, nutritional status, sex, and training background. Therefore, these biomarkers were not prioritized as core candidate biomarkers in the present framework but were instead interpreted as auxiliary exercise-responsive markers with context-dependent utility.

The integration of metabolomics evidence extended the conventional biomarker analysis by providing a broader view of metabolic adaptations associated with acute exercise. Among the identified metabolites, hypoxanthine and xanthine showed relatively consistent increases across multiple studies and biological matrices (Figure 12), suggesting altered purine turnover and ATP metabolism during strenuous exercise. Together with lactate and pyruvate responses (Figure 7), these findings support a metabolic framework involving glycolytic activity and purine degradation as important components of exercise-responsive biochemical adaptation. The observed elevation of purine-related metabolites is consistent with previous evidence linking ATP turnover and purine metabolism to exercise-induced metabolic stress [150]. Nevertheless, because the metabolomics synthesis was based on reported fold-change information and individual-level quantitative data were not consistently available, these findings should be considered hypothesis-generating rather than definitive biomarker discovery.

The interpretation of exercise-responsive metabolites is also influenced by biological matrix differences. Blood, plasma, serum, saliva, sweat, urine, and interstitial fluid represent distinct biological compartments with different metabolite concentrations, transport mechanisms, and temporal kinetics. Although biofluid type was extracted and summarized in the present study, matrix-specific meta-analysis was not feasible for all biomarkers because of uneven data availability across biomarker–matrix combinations. Therefore, the pooled estimates should be interpreted as overall acute exercise-response patterns rather than universally applicable effects across all biological matrices. In particular, saliva provides a convenient and non-invasive sampling approach, but salivary metabolite concentrations may be affected by salivary flow rate, oral metabolism, hydration status, and other pre-analytical factors. Previous studies have highlighted both the potential utility and the limitations of saliva as a biological matrix for metabolic assessment [151,152]. In the present study, plasma and saliva showed similar directional responses for several metabolites, and exploratory within-participant association analyses were performed; however, the limited sample size and lack of saliva normalization based on flow rate, osmolality, or total protein prevent definitive conclusions regarding plasma–saliva concordance. Future studies should incorporate standardized saliva collection procedures and matrix-specific validation strategies.

Although sweat and urine represent attractive non-invasive biological matrices, their interpretation requires additional consideration. Sweat metabolite composition is strongly influenced by sweating rate, environmental conditions, and hydration status, whereas urine metabolites reflect both systemic production and renal clearance processes, which may not directly correspond to acute metabolic changes at the time of exercise [153]. Therefore, future investigations should carefully consider matrix-specific sampling strategies when evaluating exercise-responsive biomarkers.

Several limitations should be considered in this study. First, the exploratory LC-MS pilot analysis was limited by the small sample size (n = 7) and the inclusion of only apparently healthy young male participants. Therefore, these findings cannot establish definitive biomarker validity or be generalized to females, older individuals, athletes, or clinical populations. Second, ten biomarkers were evaluated across two biological matrices without formal multiple-testing correction; therefore, the LC-MS findings should be interpreted as exploratory and hypothesis-generating rather than confirmatory evidence. Third, caffeine was used as a practical single internal standard during LC-MS analysis. Although matrix-matched calibration curves and quality-control samples were applied, a single internal standard may not fully correct compound-specific differences in extraction efficiency, matrix effects, and ionization variability among chemically diverse analytes, including purine derivatives, organic acids, amino acids, and steroid hormones. Future studies should apply stable isotope-labeled or analyte-class-specific internal standards to improve quantitative accuracy. In addition, substantial heterogeneity was observed among several meta-analytic outcomes. Although prediction intervals were calculated to characterize the expected variability of future studies, the pooled estimates should be interpreted cautiously. Differences in exercise duration and intensity, exercise modality, sampling timing, biological matrices, participant characteristics, training status, sex distribution, nutritional and hydration conditions, analytical platforms, and methodological quality may contribute to the observed heterogeneity. These factors should be systematically controlled or considered in future biomarker validation studies. Furthermore, several pre-analytical and physiological factors were not fully controlled in the LC-MS pilot experiment. Although caffeine restriction, recent exercise avoidance, and mouth rinsing were implemented, additional factors including diet, hydration status, oral hygiene, circadian variation, salivary flow rate, exact sampling timing, and plasma-volume changes were not comprehensively standardized. Exercise-induced hemoconcentration may influence plasma metabolite concentrations, whereas reduced salivary flow or local oral metabolism may affect apparent salivary concentrations. Therefore, future studies should incorporate plasma-volume correction, saliva flow-rate or dilution normalization, standardized sampling procedures, and independent fatigue criteria to determine whether these metabolic signatures can distinguish fatigue-related changes from general exercise responses.

## 5. Conclusions

This study establishes a metabolism-centered framework for identifying candidate acute exercise-responsive biomarkers associated with physical fatigue by integrating conventional biomarker meta-analysis, exercise-related metabolomics synthesis, and exploratory paired plasma–saliva targeted LC-MS analysis. Lactate, pyruvate, hypoxanthine, and xanthine emerged as candidate metabolites linking glycolytic activity and purine metabolism, two pathways closely associated with acute exercise-related energy turnover and physiological stress. Exploratory LC-MS analysis showed similar directional post-exercise responses of these metabolites in plasma and saliva, supporting saliva as a potential non-invasive matrix for future biomarker studies. However, these metabolites should be interpreted as candidate exercise-responsive biomarkers rather than definitive fatigue-specific biomarkers, as current evidence cannot distinguish fatigue from exercise exposure itself. Further validation in larger and diverse cohorts using standardized protocols and independent fatigue criteria is required before practical application.

## Data Availability

All data generated or analyzed during this study are included in this published article and its Supplementary Materials Files.

## Supplementary Materials

The following supporting information can be downloaded at: https://www.mdpi.com/article/10.3390/metabo16070509/s1, Figure S1. Schematic representation of the physical fatigue induction protocol. Figure S2. Distribution of exercise types and sample types across all 129 experiments. ISF, interstitial fluid; EE, endurance exercise; RE, resistance exercise; HIIT, high intensity interval tranning; MIX, exercise mixing two or three of EE, RE, and HIIT. Figure S3. Funnel plot from a random-effects model for changes in biomarkers before and after exercise-induced fatigue. (A) lactate; (B) CK; (C) LDH; (D) IL-6; (E) cortisol; (F) testosterone; (G) urea; (H) glucose; (I) CRP. RR, Response ratio; EE, endurance exercise; RE, resistance exercise; HIIT, high intensity interval training; MIX, exercise mixing two or three of EE, RE, and HIIT. Figure S4. Forest plot of post-exercise/pre-exercise response ratios for cortisol concentration. 95%CI, 95% confidence interval; Chi^2^, Chi-squared test; df, Degrees of freedom; I^2^, heterogeneity test; EE, endurance exercise; RE, resistance exercise; HIIT, high intensity interval training; MIX, exercise mixing two or three of EE, RE, and HIIT. Figure S5. Forest plot of post-exercise/pre-exercise response ratios for testosterone concentration. 95%CI, 95% confidence interval; Chi^2^, Chi-squared test; df, Degrees of freedom; I^2^, heterogeneity test; EE, endurance exercise; RE, resistance exercise; HIIT, high intensity interval training; MIX, exercise mixing two or three of EE, RE, and HIIT. Figure S6. Forest plot of post-exercise/pre-exercise response ratios for urea concentration. 95%CI, 95% confidence interval; Chi^2^, Chi-squared test; df, Degrees of freedom; I^2^, heterogeneity test; EE, endurance exercise; RE, resistance exercise; HIIT, high intensity interval training. Figure S7. Forest plot of post-exercise/pre-exercise response ratios for glucose concentration. 95%CI, 95% confidence interval; Chi^2^, Chi-squared test; df, Degrees of freedom; I^2^, heterogeneity test; EE, endurance exercise; RE, resistance exercise; HIIT, high intensity interval training. Figure S8. Forest plot of post-exercise/pre-exercise response ratios for CRP concentration. 95%CI, 95% confidence interval; Chi^2^, Chi-squared test; df, Degrees of freedom; I^2^, heterogeneity test; EE, endurance exercise; RE, resistance exercise; HIIT, high intensity interval training. Figure S9. Flowchart of metabolomics-related literature search and screening. Figure S10. Distribution of exercise modalities and biological matrices across included metabolomics studies. The stacked bars represent the number of independent metabolomics experiments/comparisons according to exercise modality. EE, endurance exercise; RE, resistance exercise, HIIT, high-intensity interval training. Figure S11. MS spectra of biomarkers. (A) caffeine (IS); (B) hypoxanthine (+); (C) hypoxanthine (−); (D) xanthine; (E) lactate; (F) pyruvate; (G) isoleucine; (H) phenylalanine; (I) tyrosine; (J) tryptophan; (K) cortisol; (L) testosterone. Figure S12. LC-MS chromatograms of biomarkers using by ChromeCore HILIC-Amide column (100 × 2.1 mm, 3 μm). (A) caffeine (IS); (B) hypoxanthine (+); (C) hypoxanthine (−); (D) xanthine; (E) lactate; (F) pyruvate. Figure S13. LC-MS chromatograms of biomarkers using by Agilent ZORBAX SB-C18 RRHT threaded column (50 × 4.6 mm, 1.8 μm). (A) caffeine (IS); (B) isoleucine; (C) phenylalanine; (D) tyrosine; (E) tryptophan; (F) cortisol; (G) testosterone. Table S1. Participant characteristics in this study. Perceived exertion was assessed using the Borg CR10 rating of perceived exertion (RPE) scale, ranging from 0 to 10, where 0 indicates “no exertion at all” and 10 indicates “maximal exertion”. RPE was recorded immediately after completion of the treadmill-induced fatigue protocol. Table S2. Descriptive summary of participant characteristics, intervention methods, and sample types across 127 single experiments. EE, endurance exercise; RE, resistance exercise; HIIT, high intensity interval training. Values are presented as mean ± SD. Table S3. Biomarkers reported at least 10 times across 129 single experiments/comparisons. “Times” represents biomarker-reporting occurrences. “Frequency” was calculated using all biomarker-reporting occurrences as the denominator (n = 522), not the number of articles or experiments. CK, creatine kinase; CRP, C-reactive protein; LDH, lactate dehydrogenase; IL-6, interleukin-6. Table S4. Prediction intervals for pooled biomarker response ratios. Table S5. Funnel-plot asymmetry assessment using Egger’s regression test and Begg’s rank correlation test. Table S6. Methodological quality assessment of included studies using an adapted JBI Critical Appraisal Checklist for Quasi-Experimental Studies. Table S7. Descriptive summary of participant characteristics, intervention methods, and sample types reported in 35 single experiments from metabolomics-related studies. (e), (f) and (g) were reported as means ± standard deviation. (h) Exercise types were divided into resistance exercise (RE), high-intensity interval training (HIIT), and endurance exercise (EE). (i) Duration of exercise intervention was reported as the actual time of exercise without break times. If a protocol consisted of different exercise intensities, the entire duration of the exercise protocol is given. “/” no information was provided on this parameter. (m) Intensity of exercise was used to determine whether physical fatigue was achieved. For RE and HIIT, the achievement of physical fatigue was directly determined based on their high intensity and metabolic characteristics. In contrast, for EE, additional screening was required based on factors such as exercise intensity and duration. Specifically, intensity thresholds were defined using maximal oxygen uptake (VO_2max_), encompassing supramax (>100% of VO_2max_), maximum exercise test (max), individual anaerobic threshold (IAT) (65–80% VO_2max_), self-paced (no information on intensity given) and aerobic (<65% VO_2max_). For self-paced and aerobic EE, only sessions lasting longer than 30 min were considered to ensure the induction of physical fatigue. The label fatigue indicated that the topic of the literature was to study physical fatigue caused by exercise. Table S8. SRM transitions and retention times of biomarkers. Table S9. Calibration range and coefficient. Table S10. Accuracy and precision of LLOQ, LQC, MQC and HQC samples in saliva. Table S11. Accuracy and precision of LLOQ, LQC, MQC and HQC samples in plasma. Table S12. LC-MS-measured concentrations of candidate biomarkers in saliva and plasma at pre-exercise and post-exercise time points in seven healthy young male volunteers are presented as mean ± SD. Table S13. Spearman correlation analysis of plasma and salivary biomarker responses in seven healthy young male volunteers. Correlation coefficients were calculated using individual plasma and saliva log_2_(post-exercise/pre-exercise) fold changes for each candidate biomarker.

## Abbreviations

ACTH, adrenocorticotropic hormone; BMI, Body Mass Index; BUN, blood urea nitrogen; Chi^2^, Chi-squared test; CI, confidence interval; CK, creatine kinase; CRP, C-reactive protein; df, degrees of freedom; EE, endurance exercise; HGH, human growth hormone; HIIT, high-intensity interval training; I^2^, heterogeneity test; IGF-1, insulin-like growth factor 1; IL-6, interleukin-6; IQR, interquartile range; IS, internal standard; ISF, interstitial fluid; IV, inverse variance; LC-MS, liquid chromatography-mass spectrometry; LDH, lactate dehydrogenase; MIX, exercise mixing two or three of EE, RE, and HIIT; RE, resistance exercise; RPE, rating of perceived exertion; TCA, tricarboxylic acid; TNF, tumor necrosis factor; VO_2max_, maximal oxygen uptake.
