# Supplementary material for: Candidate Metabolic Biomarkers for Physical Fatigue Assessment: An Integrative Meta-Analysis and Exploratory Liquid Chromatography–Mass Spectrometry Pilot Study of Acute Exercise Responses
Source: Metabolites. 2026 Jul 21;16(7):509. doi: 10.3390/metabo16070509 (PMC13414141; doi:10.3390/metabo16070509)
Supplement: Supplementary file 1 [file metabolites-16-00509-s001.zip › metabolites-4400487-supplementary.pdf]

Supplementary Materials

Candidate Metabolic Biomarkers for Physical Fatigue Assessment: An Integrative Meta-Analysis and Exploratory Liquid Chromatography–Mass Spectrometry Pilot Study of Acute Exercise Responses

1. Materials and Methods

1.1 Chemicals and reagents

Hypoxanthine, xanthine, isoleucine, phenylalanine, tyrosine, tryptophan, cortisol, testosterone standards were purchased from China National Institutes for Drug Control (Beijing, China). Lactate, pyruvate and 20 μg·mL^{-1} caffeine stock solution (internal standard, IS) standards were obtained from J&K Scientific (Beijing, China), Solarbio (Beijing, China) and Thermo Fisher Scientific (Waltham, MA, USA), respectively. LC-MS grade methanol and acetonitrile were provided from Sigma Aldrich (Louis, MO, USA). LC-MS grade ammonium acetate was obtained from Fisher Chemical (Loughborough, UK).

1.2 Preparation of calibration standards

The initial stock solutions of caffeine, hypoxanthine, lactate, pyruvate, isoleucine, phenylalanine, tyrosine, and tryptophan were prepared by dissolving each compound in LC-MS grade water at a concentration of 1 mg·mL^{-1}. For the dissolution of hypoxanthine, 10 μL of 1 mol·L^{-1} NaOH was added to facilitate solubilization in water. Cortisol and testosterone were dissolved in methanol and acetonitrile, respectively. These stock solutions were subsequently diluted with methanol to a final concentration of 1 μg·mL^{-1} for the development of the mass spectrometry method. Calibration standards were prepared by diluting the above stock solutions with blank plasma or saliva. Due to the high endogenous levels of lactate and pyruvate in plasma and saliva, stock solutions of lactate and pyruvate were specially prepared at a concentration of 120 mg·mL^{-1} to establish the calibration curves.

1.3 Instrument settings

LC-MS analysis was performed on a liquid chromatography system consisting of binary pumps, an autosampler and a column oven, which is coupled with a triple quadrupole mass spectrometer (Thermo Fisher Scientific, Waltham, MA, USA) consisting of a positive and negtive electrospray ionization (ESI) source. The ion spray voltage was set at 3500 V for positive ion while 2800 V was set for negtive ion. The ion transfer tube temperature was 320 °C, and the vaporizer temperature was 350 °C. The sheath gas, auxiliary gas, and ion sweep gas pressures were set at 40, 10, and 0 Arb, respectively. The cycle time was set at 0.6 s for all compounds. For isoleucine, phenylalanine, tyrosine, tryptophan, cortisol, and testosterone, chromatographic separation was carried out using an Agilent ZORBAX SB-C18 RRHT threaded column (50 × 4.6 mm, 1.8 μm), with mobile phase A (0.1% formic acid aqueous solution) and phase B (methanol), and with a gradient elution procedure as follows: 0 - 1 min, 95% phase B; 1 - 8 min, 95% - 50% phase B; 8 - 10 min, 50% phase B; 10 - 10.1 min, 50% - 95% phase B; 10.1 - 15 min, 95% phase B. For hypoxanthine, xanthine, lactate, and pyruvate,

chromatographic separation was achieved on a ChromeCore HILIC-Amide column (100 × 2.1 mm, 3 μm), with mobile phase A (20 mM ammonium acetate aqueous solution) and phase B (acetonitrile), and with a gradient procedure as follows: 0 - 1 min, 5% phase B; 1 - 8 min, 5% - 95% phase B; 8 - 12 min, 95% phase B; 12 - 12.1 min, 95% - 5%; 12.1 - 15 min, 5% phase B. Both columns were maintained at a temperature of 25°C and a flow rate of 0.3 mL·min-1. Total run time per injection was 15 min.

1.4 Calibration curves

Linearity ranges were established on the basis of concentrations of at least 6 calibration samples. The concentration of IS along the calibration curve was maintained at a constant level of 50 ng·mL^{-1}. The least-square linear regression method that takes the peak area ratio as a function of the individual plasma concentration was adopted to acquire regression equation, correlation coefficient (r^2), the slope and y-intercept. The calibration curves were deemed acceptable when the deviations were ≤ 15 %. The lower limit of quantification (LLOQ) refers to the lowest quantitatively detectable concentrations of samples. As required by the US Food and Drug Administration (FDA) and the European Medicines Agency (EMA) guidelines, it was considered to be agreeable as the calculation values of precision and accuracy ≤ 20 %.

1.5 Accuracy and precision

Accuracy and precision were evaluated by analyzing quality control (QC) samples at low (LQC), medium (MQC), and high (HQC) concentration levels (Table S8, Table S9) across multiple runs. Accuracy is expressed as the percentage of the measured value relative to the nominal value. Precision is expressed as the relative standard deviation (RSD). The accuracy should be within ± 15% of the nominal value, and the precision (RSD) should not exceed 15% for all concentration levels.

1.6 Sample preparation

Frozen plasma and saliva samples were completely thawed at room temperature before sample preparation. Then, 100 μL of IS working solution (caffeine, 100 ng·mL^{-1}) was added to 100 μL of saliva or plasma. Subsequently, 1 mL of acetonitrile and ethyl acetate was added to the saliva and plasma samples, respectively. After vortex mixing, the samples were incubated at room temperature with shaking for 10 minutes, followed by centrifugation at 13,000 ×g for 10 minutes at 4 °C The supernatant was collected and evaporated to dryness using a centrifugal concentrator (Thermo scientific). The residue was reconstituted in 200 μL of the initial mobile phase. For the analysis of isoleucine, phenylalanine, tyrosine, tryptophan, cortisol, and testosterone, samples were reconstituted in 5% methanol in water. For the analysis of hypoxanthine, xanthine, lactate, and pyruvate, samples were reconstituted in 95% acetonitrile in water. After reconstitution, the samples were centrifuged again at 13,000 ×g for

10 minutes at 4 °C, and the supernatants were collected for LC-MS analysis.

2. Secondary conventional biomarkers: cortisol, testosterone, urea, glucose, and CRP

Figures S4–S8 show additional random-effects meta-analyses for secondary conventional biomarkers that were reported in at least 10 independent comparisons but were not prioritized as core candidate biomarkers because of weaker, more heterogeneous, or context-dependent responses.

3. Establishment and verification of LC-MS methodology for exercise-induced fatigue biomarkers

To establish a robust LC-MS-based quantitative detection method for fatigue-related biomarkers, electrospray ionization (ESI) was performed in both positive and negative modes to assess the ionization profiles of the selected biomarkers. As shown in Figure S10, all target biomarkers produced distinct fragment ion peaks, confirming their reliable ionization and detection in mass spectrometry. The specific selected reaction monitoring (SRM) transitions used for quantification are summarized in Table S6. Under optimized liquid chromatography conditions, the retention times and corresponding SRM transitions for each biomarker were determined and are presented in Table S6, Figure S11 and Figure S12. These results established the analytical parameters necessary for accurate detection. For quantitative analysis of fatigue biomarkers in saliva and plasma, standard calibration curves were generated for each biomarker. As shown in Table S7, all biomarkers demonstrated good linearity in both saliva and plasma matrices, with correlation coefficients (r^2) ≥ 0.99.

Table S8 and Table S9 illustrated the accuracy and precision of LLOQ, LQC, MQC and HQC samples in saliva and plasma,respectively. The accuracy and precision of all the QC samples were ≤ 15%, while all the LLOQ were ≤ 20%. Concentrations of each biomarker in saliva and plasma samples collected before and after exercise-induced fatigue were subsequently calculated based on their respective standard curves.

4. Content of figures

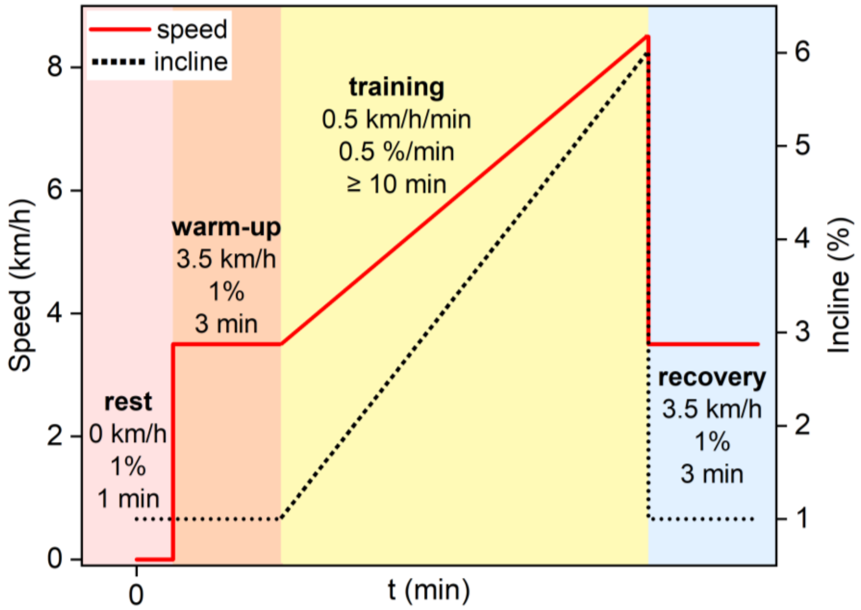

Figure S1. Schematic representation of the physical fatigue induction protocol.

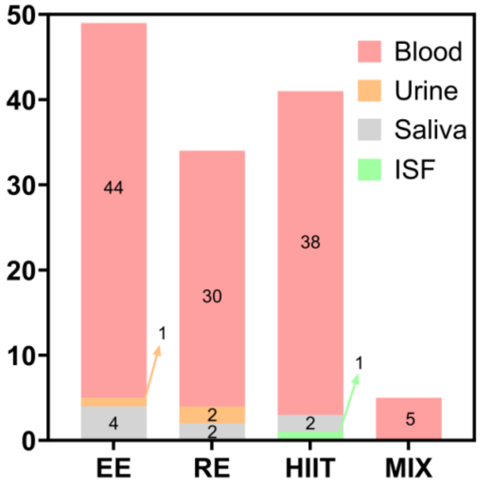

Figure S2. Distribution of exercise types and sample types across all 129 experiments. ISF, interstitial fluid; EE, endurance exercise; RE, resistance exercise; HIIT, high intensity interval tranning; MIX, exercise mixing two or three of EE, RE, and HIIT.

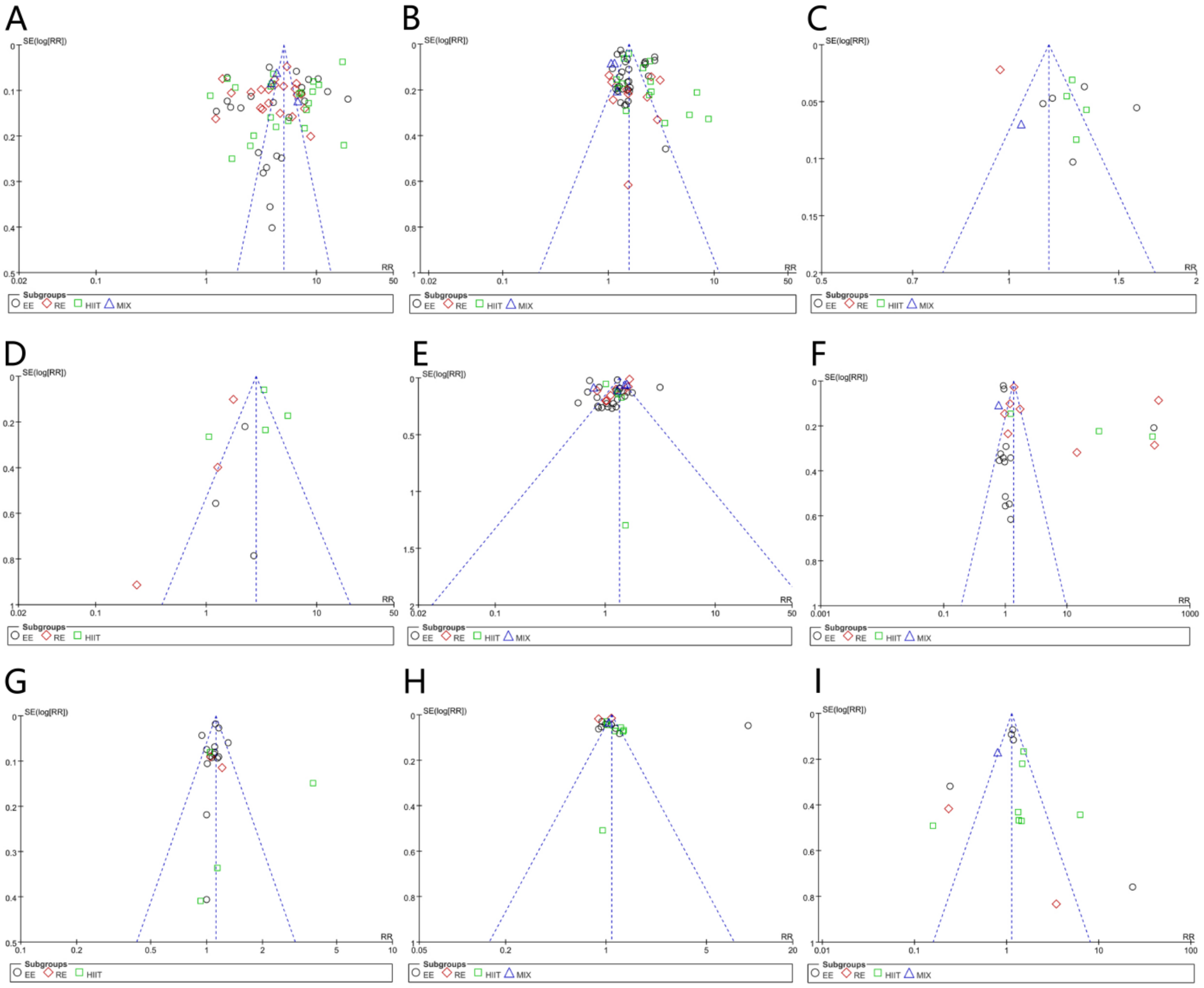

Figure S3. Funnel plot from a random-effects model for changes in biomarkers before and after exercise-induced fatigue. (A) lactate; (B) CK; (C) LDH; (D) IL-6; (E) cortisol; (F) testosterone; (G) urea; (H) glucose; (I) CRP. RR, Response ratio; EE, endurance exercise; RE, resistance exercise; HIIT, high intensity interval training; MIX, exercise mixing two or three of EE, RE, and HIIT.

Study or Subgroup	log[Response Ratio]	SE	Weight	Response Ratio IV, Random, 95% CI
**EE**				
Akimoto 2003	1.1399	0.0888	2.9%	3.13 [2.63, 3.72]
Andrzejewski 2022	0.2926	0.0922	2.9%	1.34 [1.12, 1.61]
Balsalobre-Fernández 2014	0.2688	0.129	2.7%	1.31 [1.02, 1.68]
Chang 2016	-0.3655	0.1285	2.7%	0.69 [0.54, 0.89]
Fornaziero 2023	-0.1714	0.1738	2.4%	0.84 [0.60, 1.18]
Hecksteden 2016	-0.125	0.0844	2.9%	0.88 [0.75, 1.04]
Julian 2017(1)	0.1421	0.2717	1.8%	1.15 [0.68, 1.96]
Julian 2017(2)	-0.1542	0.2536	1.9%	0.86 [0.52, 1.41]
Julian 2017(3)	0.175	0.2287	2.1%	1.19 [0.76, 1.86]
Julian 2017(4)	-0.139	0.2632	1.9%	0.87 [0.52, 1.46]
Julian 2017(5)	0.0556	0.2607	1.9%	1.06 [0.63, 1.76]
Julian 2017(6)	-0.0685	0.2669	1.9%	0.93 [0.55, 1.58]
Julian 2017(7)	0.0267	0.2276	2.1%	1.03 [0.66, 1.60]
Julian 2017(8)	-0.561	0.2255	2.1%	0.57 [0.37, 0.89]
Julian 2017(9)	0.2616	0.1916	2.3%	1.30 [0.89, 1.89]
Jówko 2018	-0.3203	0.0288	3.1%	0.73 [0.69, 0.77]
Marcos-Serrano 2018	0.2311	0.1184	2.7%	1.26 [1.00, 1.59]
Ruan 2022(1)	0.2209	0.2608	1.9%	1.25 [0.75, 2.08]
Ruan 2022(2)	0.5589	0.1329	2.7%	1.75 [1.35, 2.27]
Ruan 2022(3)	0.4156	0.161	2.5%	1.52 [1.11, 2.08]
Utter 2007	0.472	0.125	2.7%	1.60 [1.25, 2.05]
Venhorst 2018	0.3083	0.098	2.8%	1.36 [1.12, 1.65]
Wu 2022	0.2563	0.0197	3.1%	1.29 [1.24, 1.34]
**Subtotal (95% CI)**			**56.1%**	**1.15 [0.97, 1.37]**
Heterogeneity: Tau² = 0.15; Chi² = 483.22, df = 22 (P < 0.00001); I² = 95%				
Test for overall effect: Z = 1.61 (P = 0.11)				
**RE**				
Arazi 2022	-0.1699	0.116	2.8%	0.84 [0.67, 1.06]
Goto 2011	0.3621	0.1235	2.7%	1.44 [1.13, 1.83]
Hecksteden 2016	0.1196	0.1549	2.5%	1.13 [0.83, 1.53]
Hough 2011	0.5068	0.0148	3.1%	1.66 [1.61, 1.71]
Räntilä 2023	0.2201	0.1015	2.8%	1.25 [1.02, 1.52]
Shigeta 2023	0.0104	0.205	2.2%	1.01 [0.68, 1.51]
Tsuda 2019	0.4805	0.0821	2.9%	1.62 [1.38, 1.90]
West 2014	0.0335	0.2077	2.2%	1.03 [0.69, 1.55]
**Subtotal (95% CI)**			**21.3%**	**1.25 [1.04, 1.51]**
Heterogeneity: Tau² = 0.06; Chi² = 57.41, df = 7 (P < 0.00001); I² = 88%				
Test for overall effect: Z = 2.33 (P = 0.02)				
**HIIT**				
Bekris 2022	0.3353	0.1723	2.4%	1.40 [1.00, 1.96]
Hecksteden 2016	0.2627	0.1459	2.6%	1.30 [0.98, 1.73]
Reichel 2020	0.0197	0.0586	3.0%	1.02 [0.91, 1.14]
Thomas 2009	0.4308	1.2955	0.2%	1.54 [0.12, 19.49]
Thompson 2001	0.2493	0.128	2.7%	1.28 [1.00, 1.65]
**Subtotal (95% CI)**			**10.9%**	**1.18 [1.01, 1.38]**
Heterogeneity: Tau² = 0.01; Chi² = 6.41, df = 4 (P = 0.17); I² = 38%				
Test for overall effect: Z = 2.13 (P = 0.03)				
**MIX**				
Fernández-Lázaro 2021	0.2588	0.1104	2.8%	1.30 [1.04, 1.61]
Lieberman 2008	-0.2384	0.084	2.9%	0.79 [0.67, 0.93]
Zhong 2020(1)	0.4048	0.0605	3.0%	1.50 [1.33, 1.69]
Zhong 2020(2)	0.4523	0.0592	3.0%	1.57 [1.40, 1.77]
**Subtotal (95% CI)**			**11.7%**	**1.25 [0.93, 1.69]**
Heterogeneity: Tau² = 0.09; Chi² = 50.77, df = 3 (P < 0.00001); I² = 94%				
Test for overall effect: Z = 1.46 (P = 0.15)				
**Total (95% CI)**			**100.0%**	**1.19 [1.06, 1.34]**
Heterogeneity: Tau² = 0.11; Chi² = 945.79, df = 39 (P < 0.00001); I² = 96%				
Test for overall effect: Z = 3.05 (P = 0.002)				
Test for subgroup differences: Chi² = 0.50, df = 3 (P = 0.92), I² = 0%				

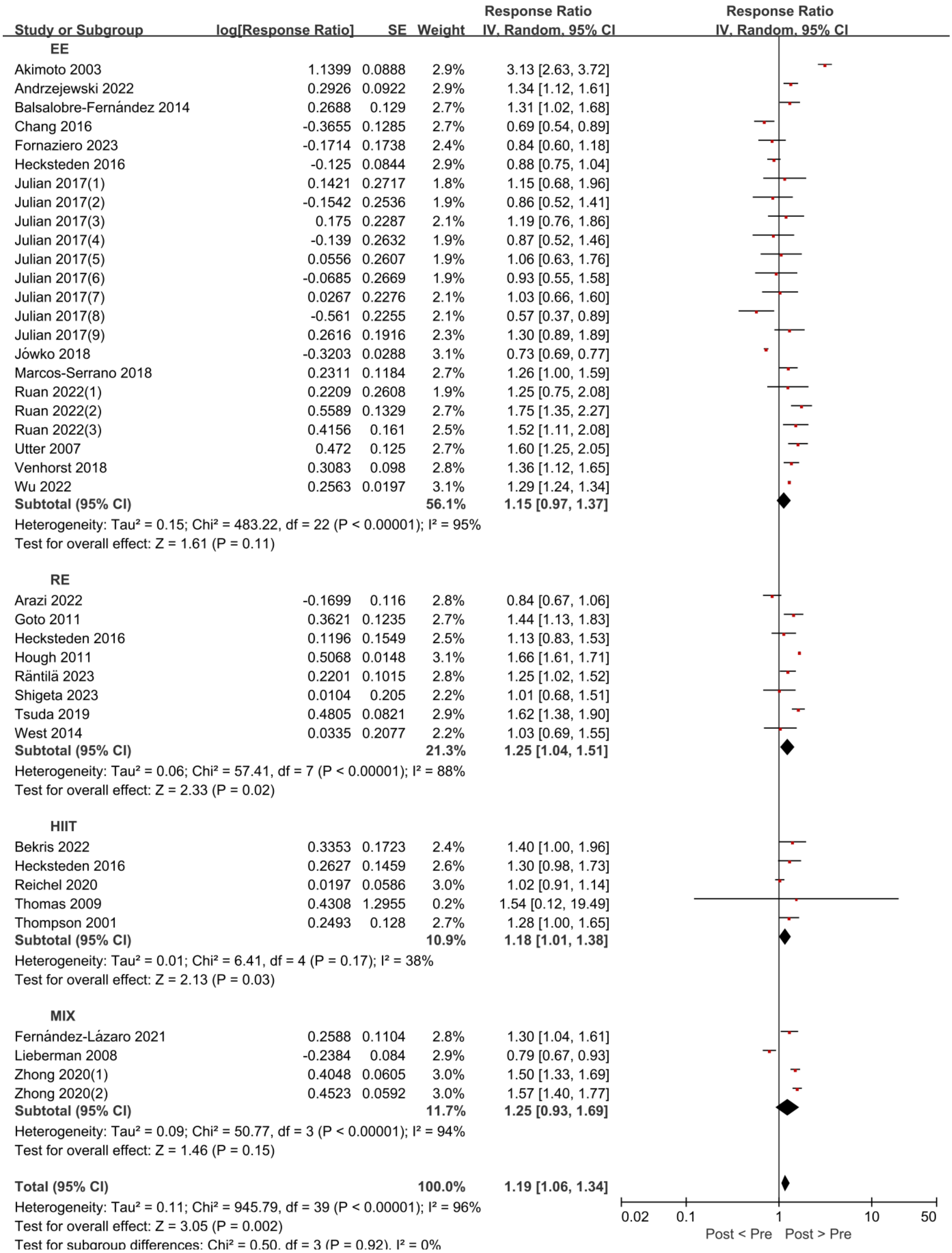

Figure S4. Forest plot of post-exercise/pre-exercise response ratios for cortisol concentration. 95% CI, 95% confidence interval; Chi², Chi-squared test; df, Degrees of freedom; I², heterogeneity test; EE, endurance exercise; RE, resistance exercise; HIIT, high intensity interval training; MIX, exercise mixing two or three of EE, RE, and HIIT. [1-26]

Study or Subgroup	log[Response Ratio]	SE	Weight	Response Ratio IV, Random, 95% CI
**EE**				
Hecksteden 2016	5.5511	0.2082	4.1%	257.52 [171.23, 387.29]
Julian 2017(1)	0.1951	0.3433	3.9%	1.22 [0.62, 2.38]
Julian 2017(2)	-0.2286	0.3547	3.9%	0.80 [0.40, 1.59]
Julian 2017(3)	-0.067	0.3426	3.9%	0.94 [0.48, 1.83]
Julian 2017(4)	-0.1806	0.326	4.0%	0.83 [0.44, 1.58]
Julian 2017(5)	-0.019	0.3599	3.9%	0.98 [0.48, 1.99]
Julian 2017(6)	0.1603	0.546	3.6%	1.17 [0.40, 3.42]
Julian 2017(7)	0	0.5572	3.6%	1.00 [0.34, 2.98]
Julian 2017(8)	-0.0107	0.5156	3.7%	0.99 [0.36, 2.72]
Julian 2017(9)	0.2095	0.6164	3.5%	1.23 [0.37, 4.13]
Jówko 2018	-0.0541	0.0368	4.2%	0.95 [0.88, 1.02]
Marcos-Serrano 2018	0.0188	0.2924	4.0%	1.02 [0.57, 1.81]
Wu 2022	-0.0765	0.0224	4.2%	0.93 [0.89, 0.97]
**Subtotal (95% CI)**			**50.6%**	**1.61 [1.02, 2.55]**
Heterogeneity: Tau² = 0.58; Chi² = 724.47, df = 12 (P < 0.00001); I² = 98%				
Test for overall effect: Z = 2.02 (P = 0.04)				
**RE**				
Arazi 2022	0.5596	0.1261	4.2%	1.75 [1.37, 2.24]
Goto 2011	5.7202	0.0861	4.2%	304.97 [257.61, 361.03]
Hecksteden 2016	5.567	0.2877	4.0%	261.65 [148.88, 459.84]
Hough 2011	0.3145	0.0288	4.2%	1.37 [1.29, 1.45]
Leal Junior 2009a	0.0882	0.2371	4.1%	1.09 [0.69, 1.74]
Räntilä 2023	0.1775	0.1017	4.2%	1.19 [0.98, 1.46]
Timon 2008	-0.0369	0.1466	4.2%	0.96 [0.72, 1.28]
West 2014	2.6619	0.3202	4.0%	14.32 [7.65, 26.83]
**Subtotal (95% CI)**			**32.9%**	**6.53 [1.18, 36.19]**
Heterogeneity: Tau² = 6.07; Chi² = 3940.34, df = 7 (P < 0.00001); I² = 100%				
Test for overall effect: Z = 2.15 (P = 0.03)				
**HIIT**				
Bekris 2022	0.21	0.1462	4.2%	1.23 [0.93, 1.64]
Hecksteden 2016	5.4884	0.2485	4.1%	241.87 [148.61, 393.65]
Thomas 2009	3.5074	0.2255	4.1%	33.36 [21.44, 51.90]
**Subtotal (95% CI)**			**12.3%**	**21.41 [0.85, 537.25]**
Heterogeneity: Tau² = 8.06; Chi² = 391.58, df = 2 (P < 0.00001); I² = 99%				
Test for overall effect: Z = 1.86 (P = 0.06)				
**MIX**				
Fernández-Lázaro 2021	-0.2505	0.1074	4.2%	0.78 [0.63, 0.96]
**Subtotal (95% CI)**			**4.2%**	**0.78 [0.63, 0.96]**
Heterogeneity: Not applicable				
Test for overall effect: Z = 2.33 (P = 0.02)				
**Total (95% CI)**			**100.0%**	**3.33 [1.93, 5.75]**
Heterogeneity: Tau² = 1.85; Chi² = 6048.78, df = 24 (P < 0.00001); I² = 100%				
Test for overall effect: Z = 4.32 (P < 0.0001)				
Test for subgroup differences: Chi² = 16.78, df = 3 (P = 0.0008), I² = 82.1%				

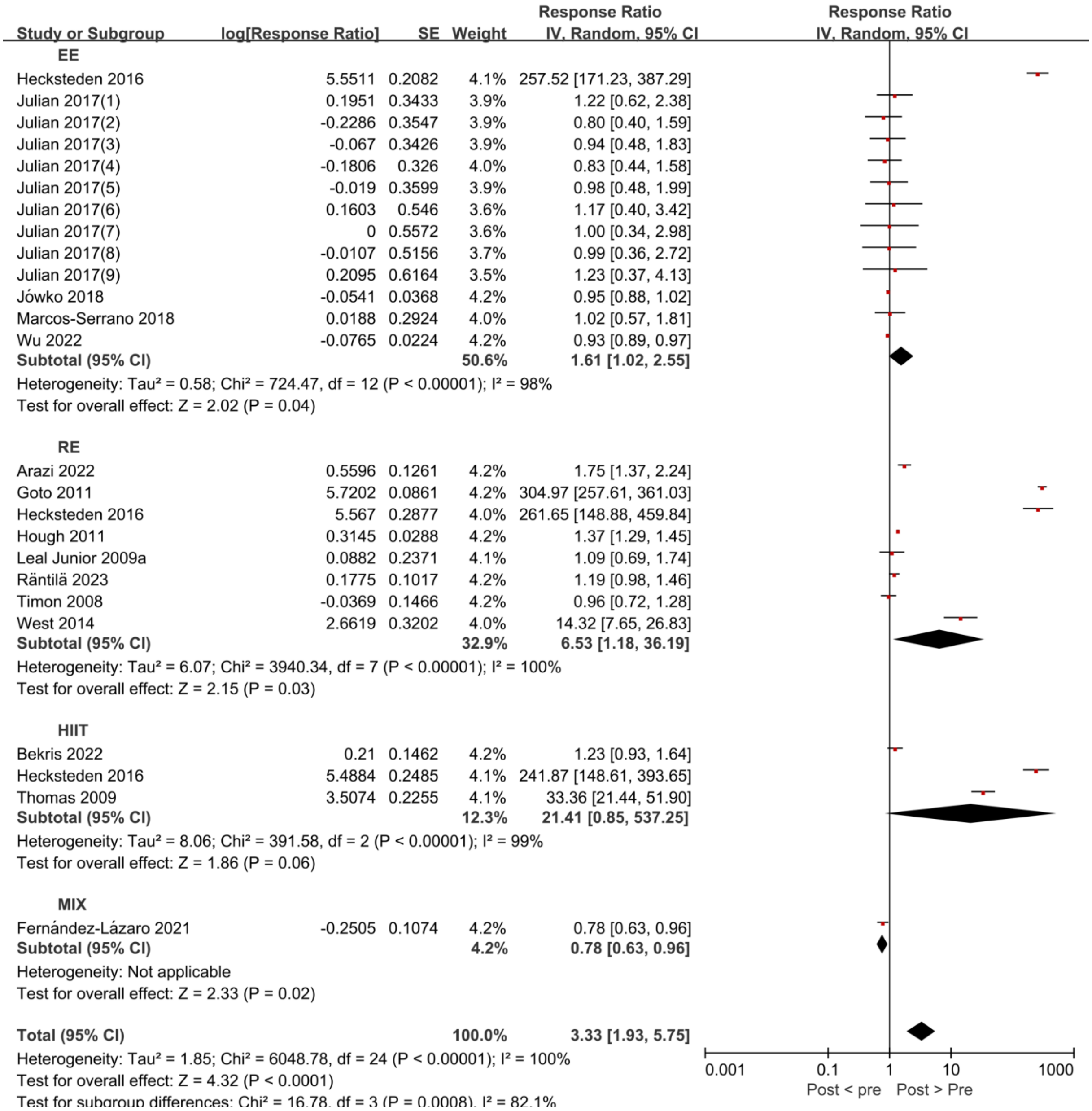

Figure S5. Forest plot of post-exercise/pre-exercise response ratios for testosterone concentration. 95% CI, 95% confidence interval; Chi², Chi-squared test; df, Degrees of freedom; I², heterogeneity test; EE, endurance exercise; RE, resistance exercise; HIIT, high intensity interval training; MIX, exercise mixing two or three of EE, RE, and HIIT. [6-9,12-16,19,20,22,27,28].

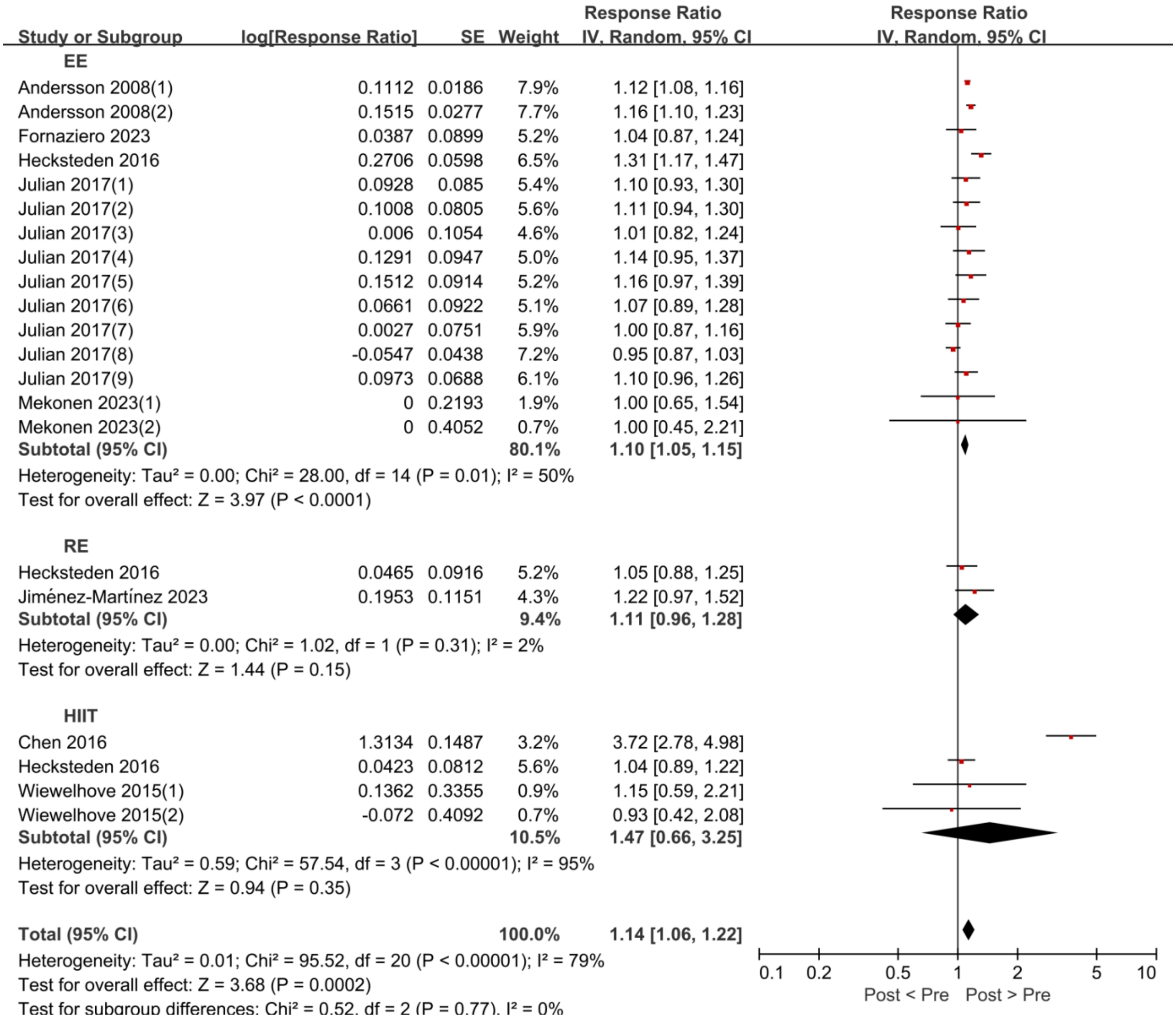

Figure S6. Forest plot of post-exercise/pre-exercise response ratios for urea concentration. 95% CI, 95% confidence interval; Chi², Chi-squared test; df, Degrees of freedom; I², heterogeneity test; EE, endurance exercise; RE, resistance exercise; HIIT, high intensity interval training. [5-7,29-33].

Study or Subgroup	log[Response Ratio]	SE	Weight	Response Ratio IV, Random, 95% CI
**EE**				
Aoi 2015	2.2783	0.0493	5.8%	9.76 [8.86, 10.75]
Chang 2016	-0.0743	0.0548	5.7%	0.93 [0.83, 1.03]
Doherty 2002	0.1515	0.0605	5.7%	1.16 [1.03, 1.31]
Fornaziero 2023	0.2168	0.0842	5.6%	1.24 [1.05, 1.46]
Krustrup 2006	0.0852	0.0465	5.8%	1.09 [0.99, 1.19]
Mekonen 2023(1)	-0.0628	0.0301	5.8%	0.94 [0.89, 1.00]
Mekonen 2023(2)	-0.01	0.0424	5.8%	0.99 [0.91, 1.08]
Utter 2007	-0.1236	0.0633	5.7%	0.88 [0.78, 1.00]
**Subtotal (95% CI)**			**45.8%**	**1.36 [0.77, 2.39]**
Heterogeneity: Tau² = 0.66; Chi² = 1923.12, df = 7 (P < 0.00001); I² = 100%				
Test for overall effect: Z = 1.07 (P = 0.28)				
**RE**				
Lee 2021	0.088	0.0156	5.8%	1.09 [1.06, 1.13]
Tsuda 2019	-0.1157	0.0183	5.8%	0.89 [0.86, 0.92]
**Subtotal (95% CI)**			**11.6%**	**0.99 [0.81, 1.20]**
Heterogeneity: Tau² = 0.02; Chi² = 71.76, df = 1 (P < 0.00001); I² = 99%				
Test for overall effect: Z = 0.13 (P = 0.89)				
**HIIT**				
Chen 2016	0.0057	0.0322	5.8%	1.01 [0.94, 1.07]
Dos 2021	-0.0558	0.508	2.5%	0.95 [0.35, 2.56]
Fiorenza 2019(1)	0.1402	0.0736	5.7%	1.15 [1.00, 1.33]
Fiorenza 2019(2)	0.281	0.0683	5.7%	1.32 [1.16, 1.51]
Mashiko 2004	0.0223	0.0462	5.8%	1.02 [0.93, 1.12]
Póvoas 2014	0.24	0.0577	5.7%	1.27 [1.14, 1.42]
Stepto 2011	0.2666	0.075	5.7%	1.31 [1.13, 1.51]
**Subtotal (95% CI)**			**36.7%**	**1.16 [1.04, 1.29]**
Heterogeneity: Tau² = 0.01; Chi² = 29.60, df = 6 (P < 0.0001); I² = 80%				
Test for overall effect: Z = 2.78 (P = 0.005)				
**MIX**				
Lieberman 2008	0.043	0.0355	5.8%	1.04 [0.97, 1.12]
**Subtotal (95% CI)**			**5.8%**	**1.04 [0.97, 1.12]**
Heterogeneity: Not applicable				
Test for overall effect: Z = 1.21 (P = 0.23)				
**Total (95% CI)**			**100.0%**	**1.22 [0.99, 1.49]**
Heterogeneity: Tau² = 0.19; Chi² = 2182.90, df = 17 (P < 0.00001); I² = 99%				
Test for overall effect: Z = 1.87 (P = 0.06)				
Test for subgroup differences: Chi² = 4.09, df = 3 (P = 0.25), I² = 26.6%				

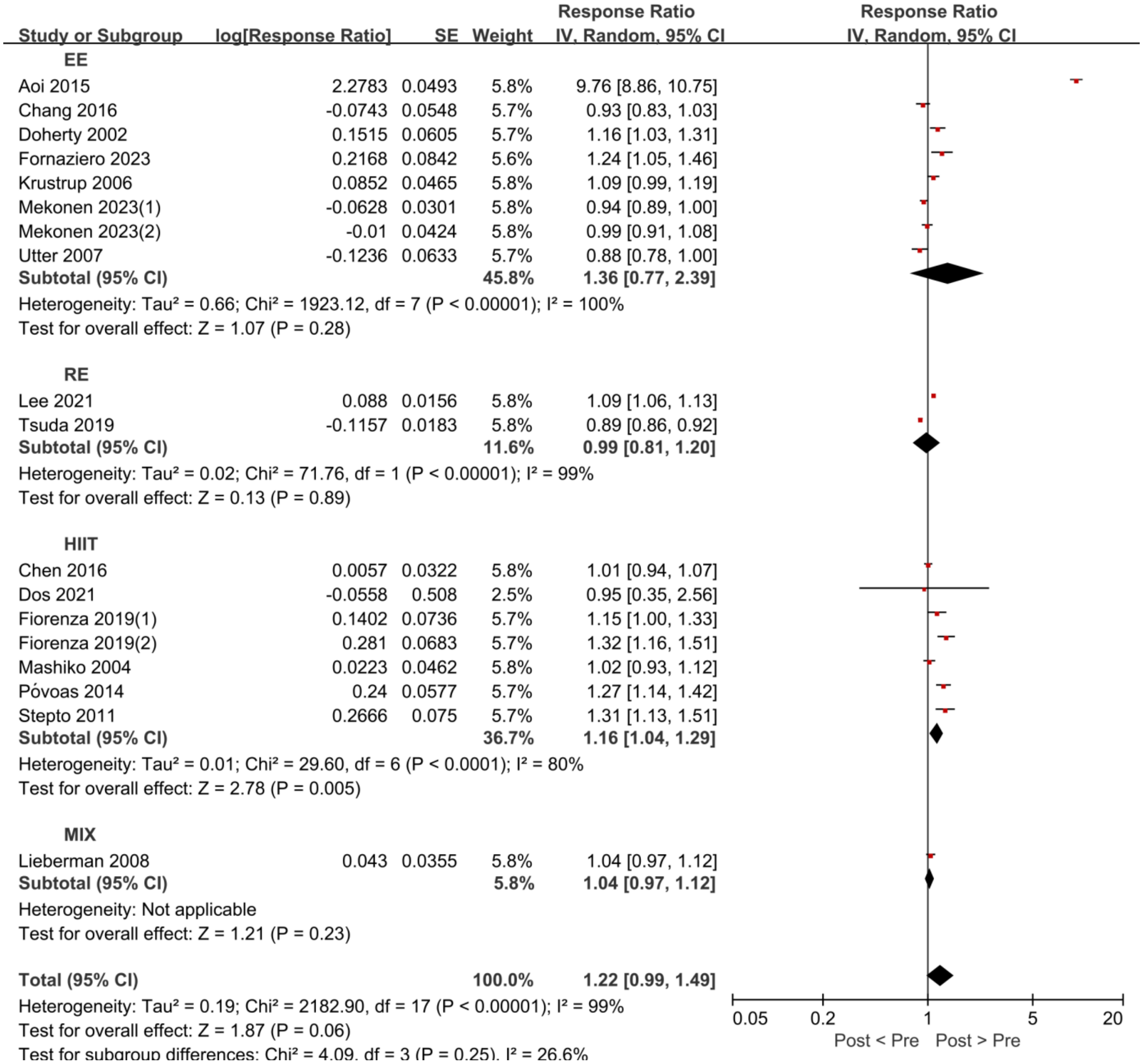

Figure S7. Forest plot of post-exercise/pre-exercise response ratios for glucose concentration. 95% CI, 95% confidence interval; Chi², Chi-squared test; df, Degrees of freedom; I², heterogeneity test; EE, endurance exercise; RE, resistance exercise; HIIT, high intensity interval training. [4,5,11,18,25,30,32,34-42].

Study or Subgroup	Response Ratio	SE	Weight	Response Ratio IV, Random, 95% CI
**EE**				
Hecksteden 2016	-1.4061	0.3189	7.0%	-1.41 [-2.03, -0.78]
Ruan 2022(1)	0.1777	0.118	9.8%	0.18 [-0.05, 0.41]
Ruan 2022(2)	0.1537	0.0714	10.2%	0.15 [0.01, 0.29]
Ruan 2022(3)	0.111	0.0942	10.0%	0.11 [-0.07, 0.30]
Wilson 2018	3.1442	0.7584	2.7%	3.14 [1.66, 4.63]
**Subtotal (95% CI)**			**39.6%**	**0.06 [-0.33, 0.45]**
Heterogeneity: Tau² = 0.14; Chi² = 39.27, df = 4 (P < 0.00001); I² = 90%				
Test for overall effect: Z = 0.30 (P = 0.76)				
**RE**				
Hecksteden 2016	-1.4513	0.4157	5.7%	-1.45 [-2.27, -0.64]
Leal Junior 2010	1.2371	0.8321	2.4%	1.24 [-0.39, 2.87]
**Subtotal (95% CI)**			**8.0%**	**-0.20 [-2.83, 2.42]**
Heterogeneity: Tau² = 3.18; Chi² = 8.35, df = 1 (P = 0.004); I² = 88%				
Test for overall effect: Z = 0.15 (P = 0.88)				
**HIIT**				
Boukhris 2020	0.2877	0.4325	5.4%	0.29 [-0.56, 1.14]
Fransson 2018	1.8369	0.4444	5.3%	1.84 [0.97, 2.71]
Hecksteden 2016	-1.8376	0.4905	4.8%	-1.84 [-2.80, -0.88]
Kotsis 2018(1)	0.4177	0.1682	9.2%	0.42 [0.09, 0.75]
Kotsis 2018(2)	0.3887	0.2199	8.4%	0.39 [-0.04, 0.82]
Wiewelhove 2015(1)	0.3001	0.4662	5.1%	0.30 [-0.61, 1.21]
Wiewelhove 2015(2)	0.3772	0.4692	5.0%	0.38 [-0.54, 1.30]
**Subtotal (95% CI)**			**43.2%**	**0.28 [-0.29, 0.85]**
Heterogeneity: Tau² = 0.44; Chi² = 31.30, df = 6 (P < 0.0001); I² = 81%				
Test for overall effect: Z = 0.97 (P = 0.33)				
**MIX**				
Radzimiński 2020	-0.2162	0.1719	9.1%	-0.22 [-0.55, 0.12]
**Subtotal (95% CI)**			**9.1%**	**-0.22 [-0.55, 0.12]**
Heterogeneity: Not applicable				
Test for overall effect: Z = 1.26 (P = 0.21)				
**Total (95% CI)**			**100.0%**	**0.09 [-0.20, 0.38]**
Heterogeneity: Tau² = 0.20; Chi² = 95.27, df = 14 (P < 0.00001); I² = 85%				
Test for overall effect: Z = 0.62 (P = 0.54)				
Test for subgroup differences: Chi² = 2.55, df = 3 (P = 0.47), I² = 0%				

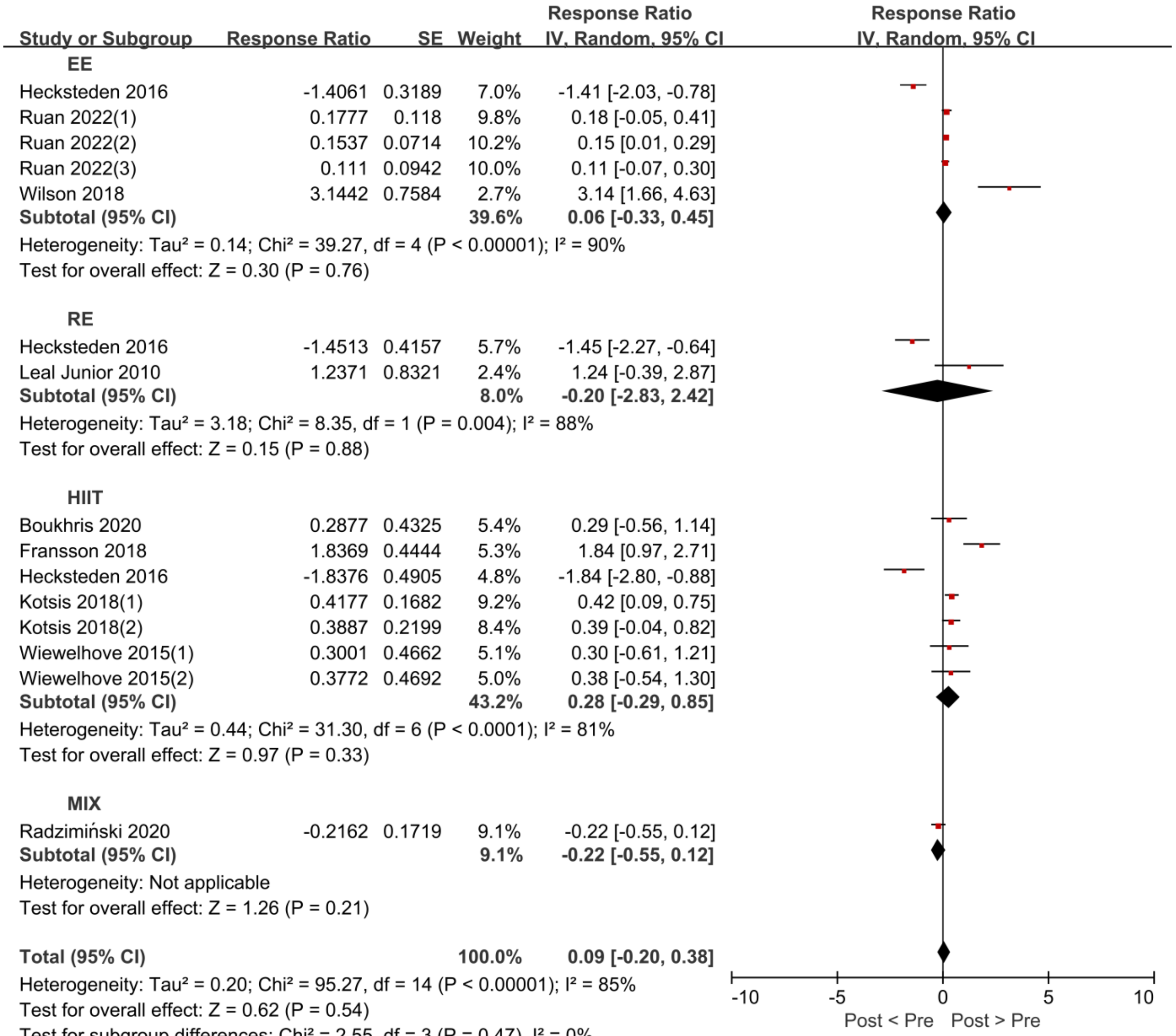

Figure S8. Forest plot of post-exercise/pre-exercise response ratios for CRP concentration. 95% CI, 95% confidence interval; Chi², Chi-squared test; df, Degrees of freedom; I², heterogeneity test; EE, endurance exercise; RE, resistance exercise; HIIT, high intensity interval training. [6,10,33,43-48].

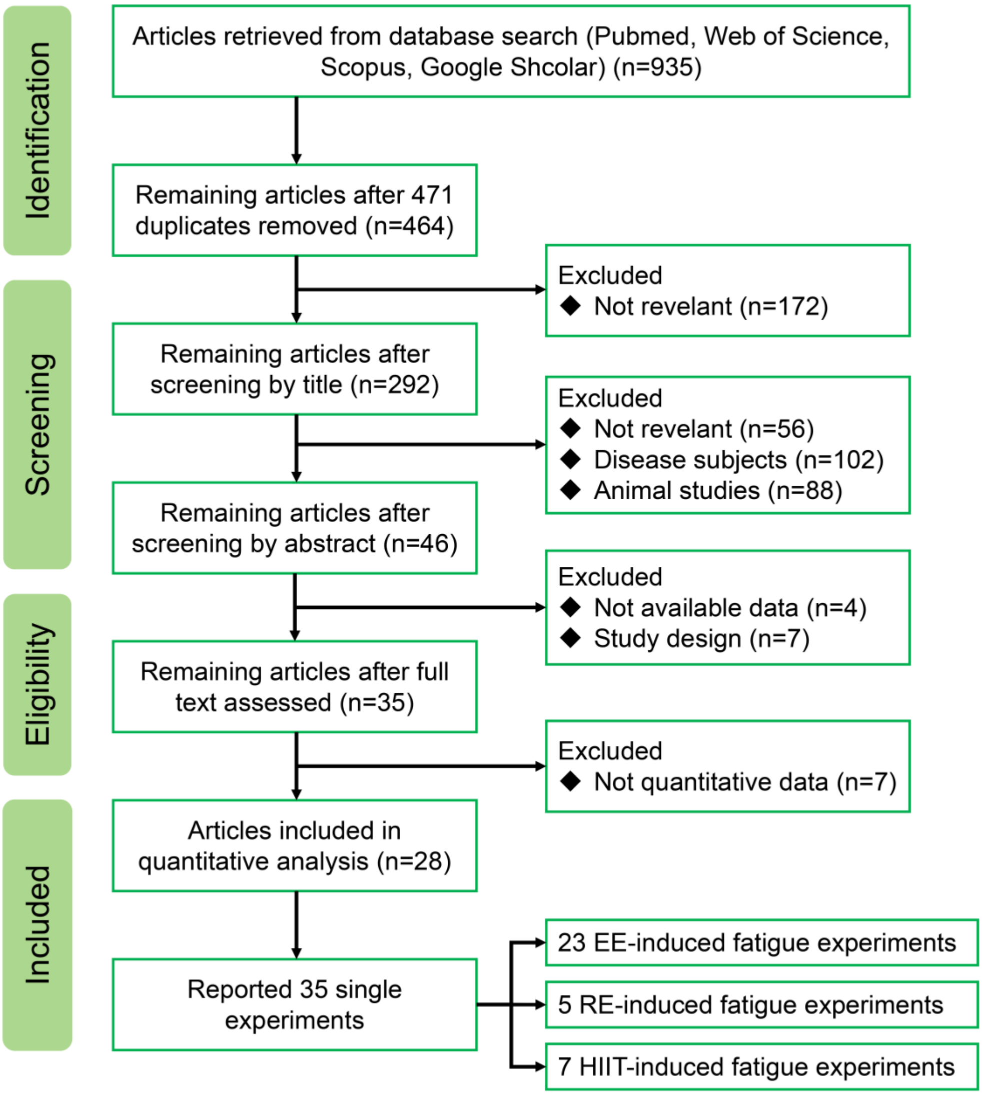

Figure S9. Flowchart of metabolomics-related literature search and screening.

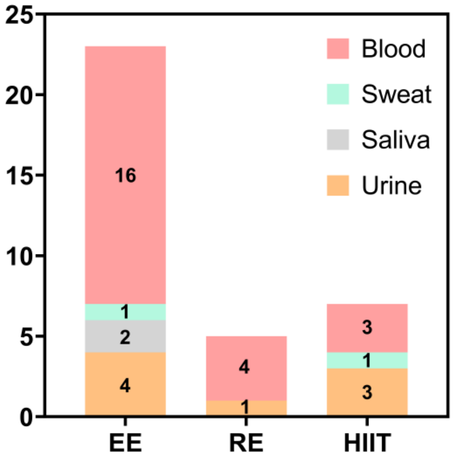

Figure S10. Distribution of exercise modalities and biological matrices across included metabolomics studies. The stacked bars represent the number of independent metabolomics experiments/comparisons according to exercise modality. EE, endurance exercise; RE, resistance exercise, HIIT, high-intensity interval training.

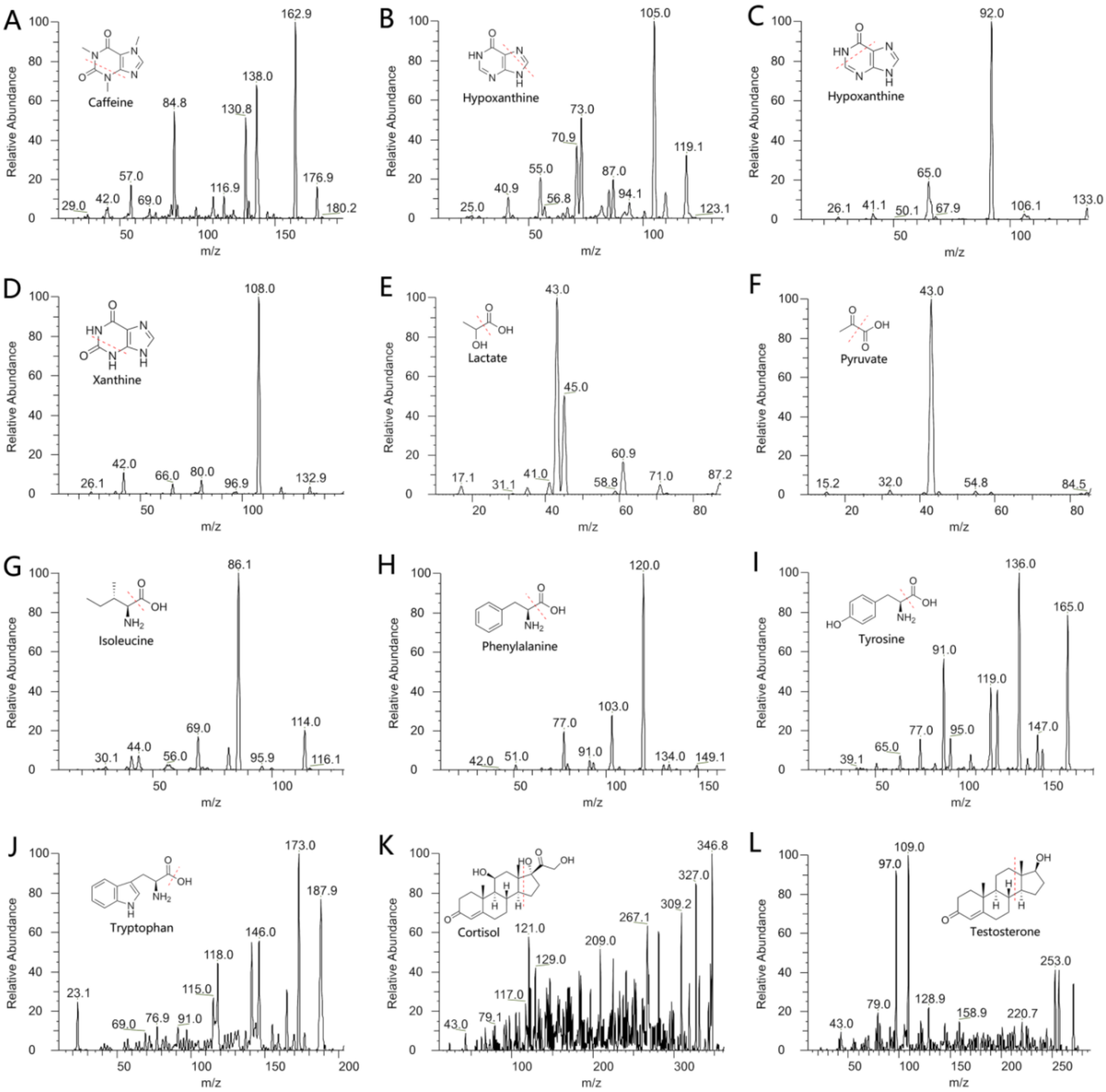

Figure S11. MS spectra of biomarkers. (A) caffeine (IS); (B) hypoxanthine (+); (C) hypoxanthine (-); (D) xanthine; (E) lactate; (F) pyruvate; (G) isoleucine; (H) phenylalanine; (I) tyrosine; (J) tryptophan; (K) cortisol; (L) testosterone.

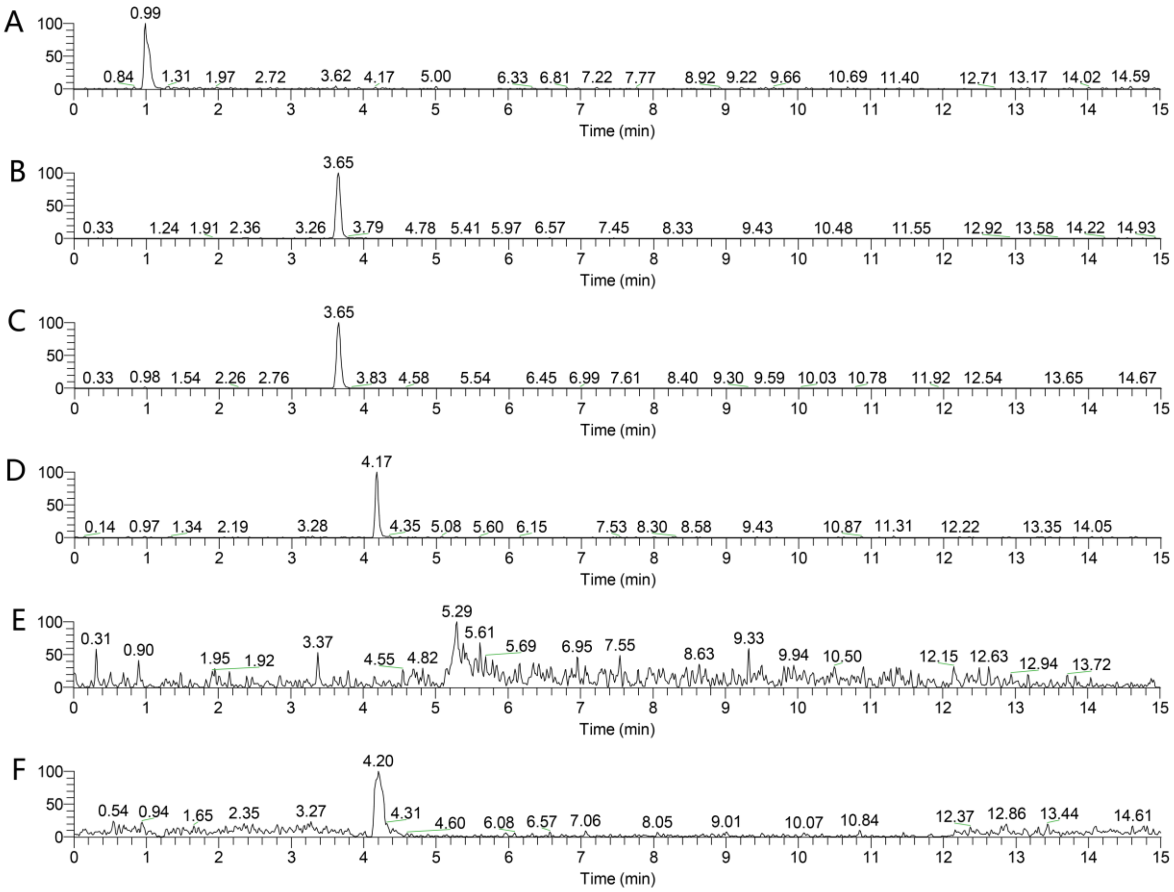

Figure S12. LC-MS chromatograms of biomarkers using by ChromeCore HILIC-Amide column (100 × 2.1 mm, 3 μm). (A) caffeine (IS); (B) hypoxanthine (+); (C) hypoxanthine (-); (D) xanthine; (E) lactate; (F) pyruvate.

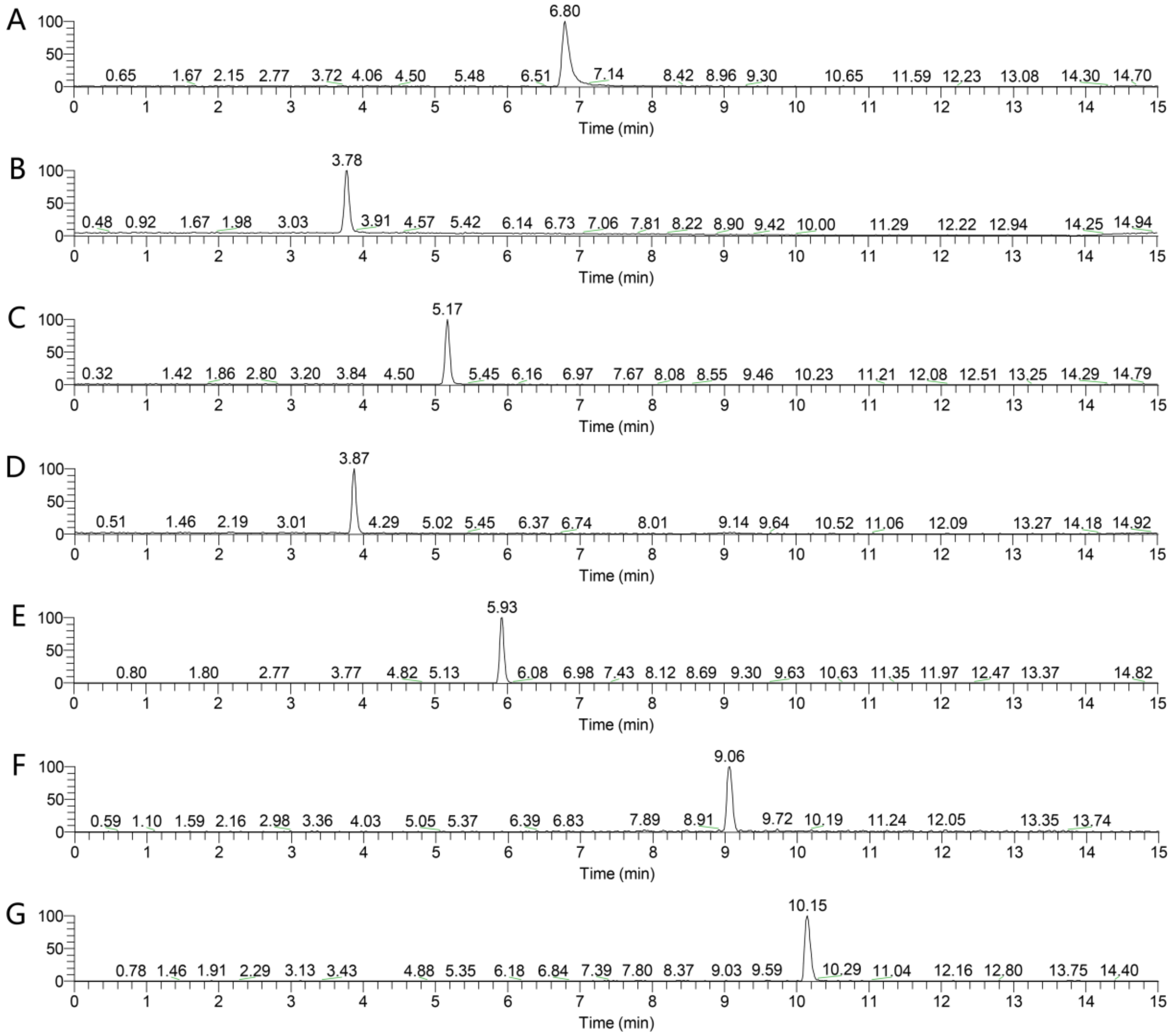

Figure S13. LC-MS chromatograms of biomarkers using by Agilent ZORBAX SB-C18 RRHT threaded column (50 × 4.6 mm, 1.8 μm). (A) caffeine (IS); (B) isoleucine; (C) phenylalanine; (D) tyrosine; (E) tryptophan; (F) cortisol; (G) testosterone.

5. Content of tables

Table S1. Participant characteristics in this study. Perceived exertion was assessed using the Borg CR10 rating of perceived exertion (RPE) scale, ranging from 0 to 10, where 0 indicates “no exertion at all” and 10 indicates “maximal exertion”. RPE was recorded immediately after completion of the treadmill-induced fatigue protocol.

Participant characteristics	Mean ± Standard Deviation
Number of participants	7 (males)
Age (years)	24.0 ± 3.1
Height (cm)	171.3 ± 5.6
Body mass (kg)	72.0 ± 7.5
Body mass index (BMI, $kg \cdot m^{-2}$)	24.7 ± 3.7
Body fat (%)	25.2 ± 5.2
RPE score (pre-exercise)	0.8 ± 0.7
RPE score (post-exercise)	8 ± 0.8

Table S2. Descriptive summary of participant characteristics, intervention methods, and sample types across 127 single experiments. EE, endurance exercise; RE, resistance exercise; HIIT, high intensity interval training. Values are presented as mean ± SD.

No.	Literature	Subjects	Male	Female	Age	Height (cm)	Weight (kg)	BMI (kg·m^{-2})	Body fat (%)	Exercise type	Duration of exercise intervention	Sample type
1	Akimoto 2003[1]	Trained	0	12	17.7 ± 2.8	158.1 ± 1.9	52.4 ± 2.9	20.2 ± 1.1	20.9 ± 1.8	EE	70min	saliva
2	Alaphilippe 2012[49]	Trained	12	0	20.5±0.9	181.5±5.8	100.1±11.4	/	/	HIIT	/	capillary blood
3	Andersson 2008[29]	Trained	0	17	active: 22.6±4.2 passive: 21.6±2.6	active: 167.1±5.7 passive: 167.2±4.7	active: 63.3±7.1 passive: 65.0±4.6	active: 22.8±1.1 passive: 23.3±1.1	/	EE	90min	serum
4	Andrzejewski 2022[2]	Trained	18	0	17.8 ± 0.9	181.6 ± 6.9	/	/	/	EE	400min	serum
5	Aoi 2015[34]	Untrained	8	0	35.9±2.0	172.6±1.9	70.6±3.2	23.8±1.2	/	EE	60min	blood
6	Arazi 2022[13]	Trained	10	0	16±1	182±7.3	78.8±11.4	23±3.3	/	RE	/	plasma
7	Areces 2015[50]	Trained	15	2	42.7 ± 7.8	173±9	69.9 ± 7.7	23.3 ± 2.9	/	EE	~210min	serum
8	Armentano 2007[51]	Trained	20	15	Male: 27.38±4.74 Female: 25.94±2.53	Male: 173.32±7.76 Female: 173.05±11.26	Male: 72.80±9.39 Female: 74.81±13.94	Male: 24.2±3.1 Female: 25.1±3.5	Male: 17.47±4.14 Female: 17.57±5.18	RE	2min	serum
9	Axelson 2002[52]	Untrained	7	4	25-73	/	/	/	/	HIIT	30min	interstitial fluid
10	Balsalobre-Fernández 2014[3]	Trained	12	3	26.3±5.1	176.2±6.1	61.1±6.5	/	/	EE	/	blood
11	Bekris 2022[20]	Trained	12	0	22.42±3.96	182.41±5.52	82.68±7.54	/	15.31±5.01	HIIT	45min	blood

12	Birat 2018(1)[53]	Untrained	12	0	19-27	/	/	/	/	EE	4min	blood
13	Birat 2018(2)[53]	Untrained	12	0	19-27	/	/	/	/	HIIT	5s	blood
14	Birat 2018(3)[53]	Untrained	12	0	19-27	/	/	/	/	RE	/	blood
15	Boukhris 2020[45]	Untrained	15	0	20±3	173±7	67±7	/	/	HIIT	3min	serum, plasma
16	Broome 2021[54]	Trained	19	0	44±4	178.2 ± 4.8	78.5±10.2	24.7 ± 2.4	/	EE	45min	plasma
17	Buckley 2010[55]	Untrained	11	0	18-30	/	/	/	/	RE	20~30min	serum, plasma
18	Chang 2016[4]	Untrained	6	6	22.6±1.1	171.3±10.5	63.6±11.2	21.7±3.2	/	EE	/	serum, plasma
19	Chen 2016[32]	Trained	12	0	20.0 ± 0.8	1.77 ± 0.04	66.9± 5.0	21.29 ± 0.93	/	HIIT	18min	serum
20	Chen 2020[56]	Trained	15	0	18-23	184.3±7.1	77.7±10.1	/	/	HIIT	10min	blood
21	Cheng 2023[57]	Untrained	15	0	20-40	/	/	<27	/	EE	30min	serum
22	Cimadevilla 2024[58]	/	13	0	28.70±6.44	174.21±8.70	75.18±10.97	24.62±1.86	/	HIIT	/	blood
23	Deb 2018[59]	Trained	11	0	28 ± 6	179.9 ± 7.2	81.7 ± 11.8	25.2 ± 2.6	/	HIIT	/	blood
24	Demura 2011[60]	Trained	10	0	23.8 ± 3.9	172.3 ± 5.5	67.7 ± 6.1	22.8 ± 1.7	/	HIIT	150s	serum, plasma
25	Detanico 2015[61]	Trained	20	0	20.7±4.6	174.0±8.7	72.8±12.6	/	13.9±3.1	EE	15min	serum
26	Doherty 2002[35]	Trained	14	0	22.7 ± 3.5	176 ± 6	70.6 ± 8.8	22.8 ± 3.3	/	EE	2-4min	plasma

27	Dong 2024[62]	Trained	24	0	21±3	177±5	71±7	22.45±1.95	/	RE	/	blood
28	Dos 2021[38]	Trained	8	2	26.9 ± 4.01	/	73.44 ± 9.57	25.30 ± 1.36	15.94 ± 4.32	HIIT	/	blood
29	Elloumi 2005[63]	Trained	11	0	26,6±1.4	180.9±1.6	91.1±3.5	27.56±1.0	19.5±1.0	HIIT	1.5h	blood
30	Farney 2019[64]	Trained	6	6	24.1 ± 3.9	/	/	/	/	RE+HIIT	24min	blood
31	Fernández-Lázaro 2021[24]	Trained	15	0	32.9 ± 6.3	174.5 ± 3.3	81.2 ± 11.5	26.7 ± 3.9	13.2 ± 5.7	HIIT+RE	1080min	serum
32	Fiorenza 2019(1)[39]	Trained	11	0	31.6±2.6	177±2.0	74.7±2.8	23.85±1.04	/	HIIT	SS, 1.5min	plasma
33	Fiorenza 2019(2)[39]	Trained	11	0	31.6±2.6	177±2.0	74.7±2.8	23.85±1.04	/	HIIT	LS, 2min	plasma
34	Fornaziero 2023[5]	Trained	6	0	17.91 ± 0.53	182.58 ± 8.40	80.85 ± 5.73	24.3 ± 2.5	11.9 ± 1.5	EE	90min	blood
35	Fransson 2018[46]	Trained	12	0	23±1	180±2	75±2	/	12.6±1.5	HIIT	90min	blood
36	Fye 2021[65]	Trained	6	5	20 ± 2	Male: 177.4 ± 15.0 Female: 163.6 ± 7.3	Male: 67.5±7.6 Female: 58.7±9.0	Male: 21.5±2.4 Female: 21.9±3.6	/	EE	38min	blood
37	Gholami 2022[66]	Trained	0	11	21.7 ± 1.2	167.0 ± 5.6	63.2 ± 4.1	23.6 ± 2.1	19.3 ± 2.4	EE	4 weeks	plasma
38	Gomez-Cabrera 2006[67]	Trained	14	0	37±8	/	/	/	/	EE	240-300min	plasma
39	Gorostiaga 2014[68]	Untrained	13	0	34.4 ± 5.4	177.4 ± 6.0	74.1 ± 6.3	/	/	RE	/	blood
40	Goto 2011[14]	Untrained	8	0	25±1	174±1	68±2	22.5±0.8	/	RE	20-25min	plasma
41	Hecksteden 2016(1)[6]	Trained	23	5	28.9±7.3	178.8±6.5	71.5±8.8	22.4±2.7	/	EE	/	serum

42	Hecksteden 2016(2)[6]	Trained	11	11	22.9±2.6	176.6±7.5	69.5±7.3	22.3±2.5	/	HIIT	/	serum
43	Hecksteden 2016(3)[6]	Trained	14	9	23.9±2.1	174.7±8.4	71.8±11.3	23.5±3.1	/	RE	/	serum
44	Hooper 2021[69]	Trained	13	0	26.2±5.3	184.3±8.2	92.9±15.6	/	/	RE		blood
45	Hough 2011[15]	Trained	10	0	24±3	/	73.2±4.8	/	/	RE	/	blood
46	Howe 2013[70]	Trained	8	0	25±5	179±7	71.1±7.5	/	/	HIIT	/	blood
47	Hsu 2005[71]	Untrained	13	0	23.0±1.6	172.5±5.2	70.2±6.3	/	/	EE	/	plasma
48	Jiménez-Martínez 2023[31]	Trained	25	0	21.00±2.15	176.4±7.5	76.5±9.5	/	/	RE	/	blood
49	Jówko 2018[8]	Trained	26	0	20.9±0.2	184.7±2.1	81.1±3.0	/	/	EE	/	blood
50	Julian 2017(1)[7]	Trained	8	4	18±3	/	/	/	/	EE	/	serum
51	Julian 2017(2)[7]	Trained	8	4	18±3	/	/	/	/	EE	/	serum
52	Kamandulis 2012[72]	Trained	9	0	19.1 ± 1.0	184.9 ± 3.4	74.1 ± 6.5	21.7 ± 1.6	/	RE	4h	plasma
53	Kamitani 2023[73]	Trained	0	20	23.15 ± 3.03	160.31 ± 4.57	53.6 ± 4.53	20.87 ± 2.33	/	HIIT	80s	blood
54	Kon 2010[74]	Trained	6	2	26.0±3.2	169.6±4.6	67.7±5.3	/	/	EE	/	saliva
55	Konidari 2025[75]	Trained	12	0	29.2 ± 3.8	175 ± 5	80.1 ± 7.6	26.3 ± 2.0	14.4 ± 4.9	HIIT		blood
56	Kotsis 2018[47]	Trained	22	0	21.8±1.4	179±5	78.2±8.1	23.0±2.1	/	HIIT	90min	blood

57	Kritikos 2021[76]	Trained	10	0	21 ± 1.5	180.0 ± 0.1	79.3 ± 6.8	24.6 ± 1.2	19.0 ± 6.7	HIIT	60min	serum
58	Krustrup 2006[36]	Trained	31	0	28±1.32	179±4	75.7±6	23.4±0.8	/	EE	/	blood, serum
59	Kunz 2019(1)[77]	Trained	11	0	16±1	178±7	67±7	/	/	HIIT	80min	blood
60	Kunz 2019(2)[77]	Trained	11	0	16±1	178±7	67±7	/	/	HIIT	80min	blood
61	Lai 2023[78]	Trained	13	0	19.15±1.07	180.31±5.04	76.15±9.72	/	/	EE	/	serum, plasma
62	Leal Junior 2009a[27]	Trained	10	0	22.3±6.09	193.9±7.14	87.78±9.27	24.42±2.58	/	RE	/	blood
63	Leal Junior 2009b[79]	Trained	10	0	23.6±5.6	194.5±6.6	87.5±9.1	/	/	RE	/	blood
64	Leal Junior 2010[44]	Trained	9	0	18.6±1.0	193.3±8.8	83.6±5.6	22.4±1.5	/	RE	5-6min	blood
65	Lee 2021[37]	Trained	15	15	21.0 ± 0.4	167.5 ± 1.1	62.7 ± 1.3	22.3 ± 0.4	20.6 ± 1.3	RE	30~40min	serum, plasma, urine
66	Lieberman 2008[25]	Trained	0	51	19.7±2.1	165.0±4.8	63.9 T 0.8	/	/	EE+HIIT +RE	/	blood
67	Lobo 2022[80]	Untrained	18	15	Male 30.5±4.3 Female 34.2±3.7	/	Male 71.6±5.9 Female 57.8±6.2	/	Male 18.1±4.5 Female 27.3±4.8	EE	/	blood
68	Marcos- Serrano 2018[9]	Trained	10	0	36.00 ± 1.27	179.29 ± 10.77	74.50 ± 1.04	23.2 ± 1.4	9.02 ± 1.04	EE	548-700min	urine
69	Mashiko 2004[40]	Trained	37	0	20.3±1.5	173.4±5.3	87.2±8.2	/	/	HIIT	/	serum
70	Matsubara 2010[81]	Untrained	12	0	23.6±0.3	171.4±1.6	66.0±2.4	22.5±0.5	16.2±1.1	EE	60min	saliva, plasma
71	McKenna 2006[82]	Trained	8	0	27.1 ± 5.6	180.3 ± 5.4	76.7 ± 10.9	23.6 ± 3.1	/	EE	50min	plasma

72	Medved 2004[83]	Trained	8	0	21.3 ± 2.3	179.4 ± 4.4	77.8 ± 10.5	24.2 ± 3.1	/	EE	1h	plasma, blood
73	Mekonen 2023(1)[30]	Trained	6	0	29 ± 5	181 ± 3	76 ± 4	23 ± 0	/	EE	CE100: 15min	plasma
74	Mekonen 2023(2)[30]	Trained	6	0	29 ± 5	181 ± 3	76 ± 4	23 ± 0	/	EE	CE75: 30min	plasma
75	Mikulski 2015[84]	Trained	11	0	32.6 ± 1.9	180 ± 2.0	73.6 ± 1.6	22.3 ± 0.3	/	RE	/	blood
76	Mroczek 2011[85]	Trained	14	0	18±1	196±7.39	84.07±7.77	/	/	HIIT	/	blood
77	Okada 2018[86]	Untrained	13	0	35.5±12.1	171.4±6.7	67.3±8.7	22.9±1.9	/	EE	30min	serum
78	Póvoas 2014[41]	Trained	40	0	26±3	187.4±8	87.5±7.9	/	19.6±2.8	HIIT	/	blood
79	Qu 2024[87]	Trained	12	0	20.41 ± 1.31	176.75 ± 3.49	70.58 ± 6.27	22.6 ± 2.3	/	EE	90min	serum
80	Radzimiński 2020[48]	Trained	15	0	24.3±5.25	182.6±6.75	76.4±6.72	/	/	EE+HIIT +RE	/	venous blood
81	Räntilä 2023[16]	Untrained	26	0	24.6 ± 3.8	180 ± 7.3	77.0 ± 10.0	23.8 ± 3.1	/	RE	60min	serum
82	Reichel 2020(1)[21]	Trained	31	31	25.3 ± 4.6	175.8 ± 10.1	72.9 ± 13.8	23.4 ± 2.8	/	EE	/	blood
83	Reichel 2020(2)[21]	Trained	31	31	25.3 ± 4.6	175.8 ± 10.1	72.9 ± 13.8	23.4 ± 2.8	/	HIIT	/	blood
84	Reichel 2020(3)[21]	Trained	31	31	25.3 ± 4.6	175.8 ± 10.1	72.9 ± 13.8	23.4 ± 2.8	/	RE	/	blood
85	Ruan 2022[10]	Untrained	53	0	20.28±1.45	174.93±4.45	68.56±9.85	22.39±2.99	/	EE	12 weeks	serum
86	Sánchez- Medina 2011[88]	/	BP: 8 SQ: 10	0	25±3.4	176.6±7.5	75.9±9.1	/	12.2±3.7	RE	/	blood

87	Santana 2018[89]	Trained	16	0	30.3±4.5	173±0.1	79.5±11.2	25.6±1.5	/	EE	/	blood
88	Shigeta 2023(1)[17]	Trained	36	0	22.3 ± 0.3	175±4	60.9±9.7	/	12.1±5.2	RE	/	saliva, blood
89	Shigeta 2023(2)[17]	Trained	36	0	22.3 ± 0.3	175±4	60.9±9.7	/	12.1±5.2	RE	/	saliva, blood
90	Shiu 2024[90]	Trained	19	0	20.9 ± 1.0	175.6 ± 4.9	66.5 ± 5.6	21.6 ± 1.9	/	EE	20min	saliva
91	Silva 2020[91]	Trained	14	0	28.9±3.5	170±50	86.1±11.1	/	/	HIIT	80min	blood
92	Souza 2024[92]	Trained	6	0	40.33±9.15	176.0±6.0	78.48±9.75	25.3±2.4	18.8±4.5	EE	255min	serum
93	Spanidis 2016[93]	Trained	14	0	26.8±1.2	199±2	101.6±2.63	/	/	HIIT	/	plasma
94	Staiano 2019[94]	Trained	13	0	16.4±0.8	172.5±7.2	60.7±7.0	/	/	EE	8.7 min	capillary blood
95	Staniszewski 2024(1)[95]	/	15	0	22±2	183±4	78±10	/	/	RE	G0: 3min	blood
96	Staniszewski 2024(2)[95]	/	15	0	20±1	183±6	78±7	/	/	RE	G45: 6.5min	blood
97	Staniszewski 2024(3)[95]	/	15	0	20±1	180±4	76±8	/	/	RE	G90: 9min	blood
98	Stepto 2011[42]	Trained	13	0	22±3	179±8	83.1±17.3	/	/	HIIT	30min	plasma
99	Stoter 2016[96]	Trained	9	0	21±3	182±6	75±6	/	/	EE	/	blood
100	Thomas 2009[22]	Untrained	17	0	15.5 ± 0.4	164.7 ± 8.1	59.1 ± 12.6	21.8 ± 2.8	22.8 ± 7.9	HIIT	48s	saliva
101	Thompson 2001[23]	Trained	9	0	28.4±1.3	175±2	79.8±4	25.6±1.3	24.04±4.14	HIIT	90min	blood, plasma, serum

102	Timon 2008[28]	Untrained	20	0	22.23±1.53	178.8±8.34	75.28±9.58	/	/	RE	/	urine
103	Tsoukos 2024(1)[97]	Trained	10	0	23.2 ± 5.1	181 ± 7	81.7 ± 10.1	24.9 ± 2.7	10.3 ± 3.9	RE	20min	blood
104	Tsoukos 2024(2)[97]	Trained	10	0	23.2 ± 5.1	181 ± 7	81.7 ± 10.1	24.9 ± 2.7	10.3 ± 3.9	RE	20min	blood
105	Tsoukos 2024(3)[97]	Trained	10	0	23.2 ± 5.1	181 ± 7	81.7 ± 10.1	24.9 ± 2.7	10.3 ± 3.9	RE	20min	blood
106	Tsuda 2019[18]	Trained	39	0	50.2±6.2	169.55±6.15	67.3±7.8	/	/	RE	/	blood
107	Utter 2007[11]	Trained	12	0	21.0±1.0	178±1	71.6±1.9	/	11.9±1.0	EE	2h	plasma
108	Varela-Olalla 2020(1)[98]	Untrained	15	0	23.0±2.4	175.0±6.0	73.1±8.2	/	/	RE	/	blood
109	Varela-Olalla 2020(2)[98]	Untrained	15	0	23.0±2.4	175.0±6.0	73.1±8.2	/	/	RE	/	blood
110	Varley 2017[99]	Trained	10	0	27.4±3.1	180±5	82.4±6.8	/	/	HIIT	>60min	serum
111	Venhorst 2018[100]	Trained	11	11	29±7<br>28±10	173±7<br>163±5	64.7±8.9<br>54.2±5.2	/	5.9±1.7<br>14.8±2.1	EE	/	serum, plasma
112	Vermeulen 2024(1)[101]	Trained	20	0	23.4 ± 4.0	186.9 ± 8.0	80.1 ± 10.2	22.9 ± 2.1	/	HIIT	5-28min	blood
113	Vermeulen 2024(2)[101]	Trained	20	0	23.4 ± 4.0	186.9 ± 8.0	80.1 ± 10.2	22.9 ± 2.1	/	HIIT	5-28min	blood
114	Volodchenko 2019[102]	Trained	8	0	17.29± 0.31	/	66.82± 3.46	/	/	HIIT	/	saliva
115	Warber 2000[103]	Trained	14	0	26±4	178.8±1.5	82.7±3.3	25.8±1.2	11.1±1	EE	4h	plasma
116	West 2014[19]	Trained	11	0	25.1±3.5	181.2±10.2	88.8±8.0	/	/	RE	/	saliva, whole blood

117	White 2015[104]	Trained	7	7	18.0±1.1<br>18.5±1.5	184.3±4.8<br>166.4±3.6	83.1±7.1<br>63.8±4.2	/	/	HIIT	/	blood
118	Whyte 2000(1)[105]	Trained	14	0	32 ± 5	180 ± 8	75 ± 9	23.1 ± 3.3	/	EE	Half-ironman: 329 ± 20	serum
119	Whyte 2000(2)[105]	Trained	14	0	32 ± 5	180 ± 8	75 ± 9	23.1 ± 3.3	/	EE	Ironman: 640 ± 74	serum
120	Wiewelhove 2015(1)[33]	Trained	11	11	Male<br>22.9 ± 1.9<br>Female<br>23.0 ± 3.4	Male<br>181.6 ± 5.3<br>Female<br>171.6 ± 6.0	Male:<br>73.8 ± 6.4<br>Female<br>65.2 ± 5.5	Male<br>22.4<br>Female<br>22.1	Male<br>14.6 ± 3.7<br>Female<br>21.1 ± 5.9	HIIT	385min	serum
121	Wiewelhove 2015(2)[33]	Trained	11	11	Male<br>22.9 ± 1.9<br>Female<br>23.0 ± 3.4	Male<br>181.6 ± 5.3<br>Female<br>171.6 ± 6.0	Male<br>73.8 ± 6.4<br>Female<br>65.2 ± 5.5	Male:<br>22.4<br>Female:<br>22.1	Male<br>14.6 ± 3.7<br>Female<br>21.1 ± 5.9	HIIT	385min	serum
122	Wiewelhove 2018[106]	Untrained	12	0	31.3±12.3	180.8±4.1	74.8±7.6	22.9±2.6	/	EE	/	serum
123	Wilson 2018[43]	Trained	10	0	40.6±7.2	174.7±8.6	75.9±10.2	/	/	EE	/	plasma
124	Wu 2022[12]	Trained	30	0	20.63 ± 1.46	178.37 ± 6.40	70.58 ± 8.96	22.8±1.9	15.2±2.5	EE	60min	plasma
125	Wu 2010[107]	Trained	9	0	21.8±2.4	173±7	67.90±11.38	/	16.11±5.01	HIIT	50min	blood
126	Yan 2025[108]	Trained	10	0	24.20 ± 1.91	174.10 ± 2.93	76.90 ± 6.19	25.4 ± 2.2	/	RE	10-15min	blood
127	Yokoi 2014[109]	Untrained	12	0	18-21	170.1±6.2	61.7±10.6	21.3±2.6	/	HIIT	/	blood
128	Zhong 2020[26]	Trained	14	0	17-22	/	/	18.5-24	/	RE+EE	50-65min	serum, plasma
129	Zory 2006[110]	Trained	7	0	24.2±3.4	176.2±4.5	78.0±6.4	/	/	EE	8min30s	capillary blood

Table S3. Biomarkers reported at least 10 times across 129 single experiments/comparisons. "Times" represents biomarker-reporting occurrences. "Frequency" was calculated using all biomarker-reporting occurrences as the denominator (n = 522), not the number of articles or experiments. CK, creatine kinase; CRP, C-reactive protein; LDH, lactate dehydrogenase; IL-6, interleukin-6.

Biomarkers	Times	Frequency
Lactate	70	13.4%
CK	59	11.3%
Cortisol	40	7.6%
Testosterone	25	4.8%
Urea	21	4.0%
Glucose	18	3.4%
CRP	15	2.9%
LDH	11	2.1%
IL-6	10	1.9%

Table S4. Prediction intervals for pooled biomarker response ratios.

**Biomarker**	**k**	**Pooled response ratio**	**τ^2**	**95% Prediction Interval**
Lactate	70	4.43	0.51	1.09–18.02
CK	59	1.70	0.09	0.93–3.12
Cortisol	40	1.19	0.11	0.62–2.29
Testosterone	25	3.33	1.85	0.22–50.51
Urea	21	1.14	0.02	0.89–1.46
Glucose	18	1.22	0.19	0.51–2.91
CRP	15	1.09	0.21	0.43–2.78
LDH	11	1.23	0.02	0.89–1.69
IL-6	10	2.15	0.22	0.80–5.76

Table S5. Funnel-plot asymmetry assessment using Egger's regression test and Begg's rank correlation test.

Biomarker	k	Egger intercept	Egger p value	Begg Kendall's tau	Begg p value
Lactate	70	-4.1113	0.0432	-0.0969	0.2355
CK	59	1.3339	0.0499	0.1274	0.1540
Cortisol	40	-1.4505	0.1630	-0.1872	0.0889
Testosterone	25	6.8893	0.0871	-0.0733	0.6273
Urea	21	0.2871	0.7075	-0.1384	0.3810
Glucose	18	5.0941	0.3265	0.2549	0.1519
CRP	15	-0.0386	0.9729	0.2190	0.2816
LDH	11	4.7056	0.0800	0.1273	0.6481
IL-6	10	-1.8059	0.1944	-0.4222	0.1083

Table S6. Methodological quality assessment of included studies using an adapted JBI Critical Appraisal Checklist for Quasi-Experimental Studies.

No.	Study	Q1 Clear cause-effect relationship	Q2 Participants described	Q3 Exercise protocol clearly described	Q4 Fatigue/ exercise endpoint defined	Q5 Pre-post measurements performed	Q6 Follow-up/ outcome completeness	Q7 Confounders/ pre-analytical factors considered	Q8 Outcome measured consistently	Q9 Biomarker measurement reliable	Q10 Statistical analysis appropriate
1	Akimoto 2003	Yes	Yes	Yes	Yes	Yes	Yes	Yes	Yes	Yes	Yes
2	Alaphilippe 2012	Yes	Yes	Yes	Unclear	Yes	Yes	Yes	Yes	Yes	Yes
3	Andersson 2008	Yes	Yes	Yes	Yes	Yes	Yes	Yes	Yes	Yes	Unclear
4	Andrzejewski 2022	Yes	Yes	Unclear	Yes	Unclear	Unclear	Yes	Yes	Yes	Yes
5	Aoi 2015	Yes	Yes	Yes	Yes	Yes	Yes	Yes	Yes	Yes	Yes
6	Arazi 2022	Yes	Yes	Yes	Yes	Yes	Yes	Yes	Yes	Yes	Yes
7	Areces 2015	Yes	Yes	Unclear	Yes	Yes	Yes	Yes	Yes	Yes	Yes
8	Armentano 2007	Yes	Yes	Yes	Unclear	Unclear	Unclear	Unclear	Yes	Yes	Unclear
9	Axelson 2002	Yes	Yes	Yes	Yes	Yes	Yes	Unclear	Yes	Yes	Unclear
10	Balsalobre-Fernández 2014	Yes	Yes	Yes	Yes	Yes	Yes	Unclear	Yes	Yes	Unclear
11	Bekris 2022	Yes	Yes	Yes	Yes	Yes	Yes	Yes	Yes	Yes	Yes
12	Birat 2018	Yes	Yes	Yes	Yes	Yes	Yes	Unclear	Yes	Yes	Yes
13	Boukhris 2020	Yes	Yes	Yes	Yes	Yes	Yes	Yes	Yes	Yes	Yes
14	Broome 2021	Yes	Yes	Yes	Unclear	Yes	Yes	Yes	Yes	Yes	Unclear
15	Buckley 2010	Yes	Yes	Unclear	Yes	Yes	Yes	Yes	Yes	Yes	Yes
16	Chang 2016	Yes	Yes	Yes	Yes	Yes	Yes	Yes	Yes	Yes	Yes

17	Chen 2016	Yes	Yes	Yes	Yes	Yes	Yes	Yes	Yes	Yes	Yes
18	Chen 2020	Yes	Yes	Yes	Yes	Yes	Yes	Yes	Yes	Yes	Yes
19	Cheng 2023	Yes	Yes	Unclear	Yes	Yes	Yes	Yes	Yes	Yes	Yes
20	Cimadevilla 2024	Yes	Unclear	Yes	Yes	Yes	Yes	Yes	Yes	Yes	Unclear
21	Deb 2018	Yes	Yes	Yes	Yes	Yes	Yes	Yes	Yes	Yes	Yes
22	Demura 2011	Yes	Yes	Unclear	Yes	Yes	Yes	Yes	Yes	Yes	Yes
23	Detanico 2015	Yes	Yes	Yes	Yes	Yes	Yes	Yes	Yes	Yes	Yes
24	Doherty 2002	Yes	Yes	Yes	Yes	Yes	Yes	Yes	Yes	Yes	Yes
25	Dong 2024	Yes	Yes	Unclear	Unclear	Unclear	Unclear	Yes	Yes	Yes	Yes
26	Dos 2021	Yes	Yes	Yes	Unclear	Unclear	Unclear	Yes	Yes	Yes	Yes
27	Elloumi 2005	Yes	Yes	Yes	Yes	Yes	Yes	Yes	Yes	Yes	Yes
28	Farney 2019	Yes	Yes	Unclear	Yes	Yes	Yes	No	Yes	Yes	Yes
29	Fernández-Lázaro 2021	Yes	Yes	Unclear	Yes	Yes	Yes	Yes	Yes	Yes	Yes
30	Fiorenza 2019	Yes	Yes	Yes	Yes	Yes	Yes	Yes	Yes	Yes	Yes
31	Fornaziero 2023	Yes	Yes	Unclear	Yes	Yes	Yes	Yes	Yes	Yes	Yes
32	Fransson 2018	Yes	Yes	Yes	Yes	Yes	Yes	Yes	Yes	Yes	Yes
33	Fye 2021	Yes	Yes	Unclear	Yes	Yes	Yes	Yes	Yes	Yes	Yes
34	Gholami 2022	Yes	Yes	Unclear	Yes	Yes	Yes	Yes	Yes	Yes	Yes
35	Gomez-Cabrera 2006	Yes	Yes	Yes	Unclear	Yes	Yes	Unclear	Yes	Yes	Yes

36	Gorostiaga 2014	Yes	Yes	Yes	Yes	Yes	Yes	Yes	Yes	Yes	Yes
37	Goto 2011	Yes	Yes	Unclear	Yes	Yes	Yes	Yes	Yes	Yes	Yes
38	Hecksteden 2016	Yes	Yes	Yes	Yes	Yes	Yes	Yes	Yes	Yes	Yes
39	Hooper 2021	Yes	Yes	Unclear	Unclear	Unclear	Unclear	Unclear	Yes	Yes	Unclear
40	Hough 2011	Yes	Yes	Unclear	Unclear	Unclear	Unclear	Yes	Yes	Yes	Unclear
41	Howe 2013	Yes	Yes	Unclear	Unclear	Yes	Yes	Unclear	Yes	Yes	Unclear
42	Hsu 2005	Yes	Yes	Yes	Yes	Yes	Yes	Yes	Yes	Yes	Yes
43	Jiménez-Martínez 2023	Yes	Yes	Yes	Unclear	Unclear	Unclear	Unclear	Yes	Yes	Yes
44	Jówko 2018	Yes	Yes	Yes	Yes	Yes	Yes	Yes	Yes	Yes	Yes
45	Julian 2017	Yes	Yes	Yes	Unclear	Yes	Yes	Yes	Yes	Yes	Yes
46	Kamandulis 2012	Yes	Yes	Unclear	Yes	Yes	Yes	Yes	Yes	Yes	Yes
47	Kamitani 2023	Yes	Yes	Unclear	Yes	Yes	Yes	Unclear	Yes	Yes	Yes
48	Kon 2010	Yes	Yes	Yes	Unclear	Yes	Yes	No	Yes	Yes	Yes
49	Konidari 2025	Yes	Yes	Yes	Yes	Unclear	Unclear	Yes	Yes	Yes	Yes
50	Kotsis 2018	Yes	Yes	Yes	Unclear	Yes	Yes	Yes	Yes	Yes	Unclear
51	Kritikos 2021	Yes	Yes	Unclear	Yes	Yes	Yes	Yes	Yes	Yes	Yes
52	Krustrup 2006	Yes	Yes	Unclear	Unclear	Yes	Yes	Yes	Yes	Yes	Unclear
53	Kunz 2019	Yes	Yes	Yes	Yes	Yes	Yes	Yes	Yes	Yes	Yes
54	Lai 2023	Yes	Yes	Yes	Yes	Yes	Yes	Yes	Yes	Yes	Yes

55	Leal Junior 2009a	Yes	Yes	Unclear	Yes	Yes	Yes	Unclear	Yes	Yes	Unclear
56	Leal Junior 2009b	Yes	Yes	Unclear	Unclear	Yes	Yes	Unclear	Yes	Yes	Unclear
57	Leal Junior 2010	Yes	Yes	Unclear	Yes	Yes	Yes	Yes	Yes	Yes	Yes
58	Lee 2021	Yes	Yes	Unclear	Yes	Yes	Yes	Yes	Yes	Yes	Yes
59	Lieberman 2008	Yes	Yes	Yes	Yes	Yes	Yes	Yes	Yes	Yes	Yes
60	Lobo 2022	Yes	Yes	Yes	Yes	Yes	Yes	Yes	Yes	Yes	Yes
61	Marcos-Serrano 2018	Yes	Yes	Unclear	Yes	Yes	Yes	Yes	Yes	Yes	Yes
62	Mashiko 2004	Yes	Yes	Yes	Yes	Yes	Yes	Yes	Yes	Yes	Yes
63	Matsubara 2010	Yes	Yes	Unclear	Yes	Yes	Yes	Yes	Yes	Yes	Yes
64	McKenna 2006	Yes	Yes	Unclear	Yes	Yes	Yes	Yes	Yes	Yes	Yes
65	Medved 2004	Yes	Yes	Unclear	Yes	Yes	Yes	Yes	Yes	Yes	Yes
66	Mekonen 2023	Yes	Yes	Unclear	Yes	Yes	Yes	Yes	Yes	Yes	Yes
67	Mikulski 2015	Yes	Yes	Yes	Unclear	Unclear	Unclear	Unclear	Yes	Yes	Unclear
68	Mroczek 2011	Yes	Yes	Yes	Yes	Yes	Yes	Unclear	Yes	Yes	Yes
69	Okada 2018	Yes	Yes	Unclear	Unclear	Yes	Yes	Yes	Yes	Yes	Yes
70	Póvoas 2014	Yes	Yes	Yes	Yes	Yes	Yes	Yes	Yes	Yes	Yes
71	Qu 2024	Yes	Yes	Unclear	Yes	Yes	Yes	Yes	Yes	Yes	Yes
72	Radzimiński 2020	Yes	Yes	Yes	Yes	Yes	Yes	Yes	Yes	Yes	Yes
73	Räntilä 2023	Yes	Yes	Unclear	Yes	Yes	Yes	Yes	Yes	Yes	Yes

74	Reichel 2020	Yes	Yes	Yes	Yes	Yes	Yes	Yes	Yes	Yes	Yes
75	Ruan 2022	Yes	Yes	Unclear	Yes	Yes	Yes	Yes	Yes	Yes	Yes
76	Sánchez-Medina 2011	Yes	Unclear	Yes	Yes	Yes	Yes	Yes	Yes	Yes	Yes
77	Santana 2018	Yes	Yes	Yes	Yes	Yes	Yes	Yes	Yes	Yes	Yes
78	Shigeta 2023	Yes	Yes	Yes	Yes	Yes	Yes	Yes	Yes	Yes	Yes
79	Shiu 2024	Yes	Yes	Unclear	Yes	Yes	Yes	Yes	Yes	Yes	Yes
80	Silva 2020	Yes	Yes	Yes	Yes	Yes	Yes	Unclear	Yes	Yes	Yes
81	Souza 2024	Yes	Yes	Unclear	Yes	Yes	Yes	Yes	Yes	Yes	Yes
82	Spanidis 2016	Yes	Yes	Yes	Unclear	Yes	Yes	Yes	Yes	Yes	Yes
83	Staiano 2019	Yes	Yes	Yes	Yes	Yes	Yes	Yes	Yes	Yes	Yes
84	Staniszewski 2024	Yes	Unclear	Unclear	Yes	Yes	Yes	No	Yes	Yes	Yes
85	Stepto 2011	Yes	Yes	Yes	Yes	Yes	Yes	Yes	Yes	Yes	Yes
86	Stoter 2016	Yes	Yes	Yes	Yes	Yes	Yes	Yes	Yes	Yes	Yes
87	Thomas 2009	Yes	Yes	Unclear	Yes	Yes	Yes	Yes	Yes	Yes	Yes
88	Thompson 2001	Yes	Yes	Yes	Yes	Unclear	Unclear	Yes	Yes	Yes	Unclear
89	Timon 2008	Yes	Yes	Yes	Yes	Yes	Yes	Yes	Yes	Yes	Yes
90	Tsoukos 2024	Yes	Yes	Unclear	Yes	Yes	Yes	Yes	Yes	Yes	Yes
91	Tsuda 2019	Yes	Yes	Unclear	Unclear	Unclear	Unclear	Yes	Yes	Yes	Yes
92	Twist 2005	Yes	Yes	Yes	Yes	Yes	Yes	Yes	Yes	Yes	Yes

93	Utter 2007	Yes	Yes	Yes	Yes	Yes	Yes	Yes	Yes	Yes	Yes
94	Varela-Olalla 2020	Yes	Yes	Yes	Yes	Yes	Yes	Yes	Yes	Yes	Yes
95	Varley 2017	Yes	Yes	Yes	Yes	Yes	Yes	Yes	Yes	Yes	Yes
96	Venhorst 2018	Yes	Yes	Yes	Yes	Yes	Yes	Yes	Yes	Yes	Yes
97	Vermeulen 2024	Yes	Yes	Yes	Yes	Yes	Yes	Yes	Yes	Yes	Yes
98	Volodchenko 2019	Yes	Yes	Yes	Unclear	Yes	Yes	Unclear	Yes	Yes	Unclear
99	Warber 2000	Yes	Yes	Yes	Yes	Yes	Yes	Yes	Yes	Yes	Yes
100	West 2014	Yes	Yes	Yes	Yes	Yes	Yes	Yes	Yes	Yes	Unclear
101	White 2015	Yes	Yes	Yes	Unclear	Yes	Yes	No	Yes	Yes	Unclear
102	Whyte 2000	Yes	Yes	Unclear	Yes	Yes	Yes	Yes	Yes	Yes	Yes
103	Wiewelhove 2015	Yes	Yes	Yes	Yes	Yes	Yes	Yes	Yes	Yes	Yes
104	Wiewelhove 2018	Yes	Yes	Unclear	Yes	Yes	Yes	Yes	Yes	Yes	Unclear
105	Wilson 2018	Yes	Yes	Unclear	Yes	Yes	Yes	Yes	Yes	Yes	Yes
106	Wu 2010	Yes	Yes	Yes	Yes	Yes	Yes	Yes	Yes	Yes	Yes
107	Wu 2022	Yes	Yes	Unclear	Yes	Yes	Yes	Yes	Yes	Yes	Yes
108	Yan 2025	Yes	Yes	Yes	Yes	Yes	Yes	Yes	Yes	Yes	Yes
109	Yokoi 2014	Yes	Yes	Yes	Yes	Yes	Yes	Yes	Yes	Yes	Yes
110	Zhong 2020	Yes	Yes	Yes	Yes	Yes	Yes	Yes	Yes	Yes	Yes

Table S7. Descriptive summary of participant characteristics, intervention methods, and sample types reported in 35 single experiments from metabolomics-related studies. (e), (f) and (g) were reported as means ± standard deviation. (h) Exercise types were divided into resistance exercise (RE), high-intensity interval training (HIIT), and endurance exercise (EE). (i) Duration of exercise intervention was reported as the actual time of exercise without break times. If a protocol consisted of different exercise intensities, the entire duration of the exercise protocol is given. "/" no information was provided on this parameter. (m) Intensity of exercise was used to determine whether physical fatigue was achieved. For RE and HIIT, the achievement of physical fatigue was directly determined based on their high intensity and metabolic characteristics. In contrast, for EE, additional screening was required based on factors such as exercise intensity and duration. Specifically, intensity thresholds were defined using maximal oxygen uptake (VO_{2max}), encompassing supramax (>100% of VO_{2max}), maximum exercise test (max), individual anaerobic threshold (IAT) (65 - 80% VO_{2max}), self-paced (no information on intensity given) and aerobic (<65% VO_{2max}). For self-paced and aerobic EE, only sessions lasting longer than 30 minutes were considered to ensure the induction of physical fatigue. The label fatigue indicated that the topic of the literature was to study physical fatigue caused by exercise.

(a) Year	(b) Author	(c) Male	(d) Female	(e) Age	(f) BMI ($kg \cdot m^{-2}$)	(g) Body fat	(h) Exercise types	(i) Duration of exercise intervention	(j) Standard diet	(k) Sample types	(l) Analysis techniques	(m) Intensity of exercise
2010	Enea[111]	0	untrained: 10	untrained: 22.1±0.6	untrained: 21.0±0.6	untrained: 23.7±0.8	RE	0.5min	yes	urine	HNMR	IAT
2010	Enea[111]	0	trained: 12	trained: 21.8±1.0	trained: 21.5±0.6	trained: 21.5±0.9	RE	0.5min	yes	urine	HNMR	IAT
2010	Pechlivanis[112]	12	0	A: 21±2	A: 22.1±1.9	/	HIIT	0.167min	yes	urine	HNMR	max
2010	Pechlivanis[112]	12	0	B: 20±1	B: 22.0±1.5	/	HIIT	0.167min	yes	urine	HNMR	max
2013	Nieman[113]	7	8	male: 35.3±9.2 female: 35.1±8.9	male: 24.54 female: 22.41	male: 16.3±4.7 female: 22.7±9.1	EE	150min	yes	serum	LC-MS/UHPLC - MS/MS	IAT
2014	Ra[114]	122	0	20.6±0.04	21.7±0.01	/	EE	270min	no	saliva	CE-TOFMS	self-paced
2014	Mukherjee [115]	17	0	control: 54.3±5.0 athletes: 53.4±3.2	control: 25.4±3.2 athletes: 24.1±2.5	/	EE	45min	yes	urine	HNMR	anaerobic

2014	Peake[116]	10	0	33.2±6.7	23.4±2.7	/	EE	60min	no	plasma	GC-MS	IAT
2014	Peake[116]	10	0	33.2±6.7	23.4±2.7	/	HIIT	60min	no	plasma	GC-MS	IAT
2015	Daskalaki [117]	3	0	32-38	/	/	EE	50min	/	urine	LC-MS	self-paced
2015	Breit[118]	27	20	34.19	23.27	/	RE	51min	/	blood	MS-MS	max
2015	Pechlivanis [119]	17	0	19±1	21.6±1.8	/	HIIT	0.167min	/	urine	RP-UPLC-MS & HNMR	max
2015	Nieman[120]	20	0	39.2±1.9	24.55	17.7±1.1	EE	120-180min	/	blood	LC-MS	self-paced
2016	Ali[121]	8	2	28±7	24.56	/	EE	45min	/	urine	LC-MS	aerobic
2016	Hecksteden [6]	cycling: 23	cycling: 5	cycling: 29±7	cycling: 22.72	/	EE	/	/	serum	other	fatigue
2016	Hecksteden [6]	HIIT: 11	HIIT: 11	HIIT: 23±3	HIIT: 22.34	/	HIIT	/	/	serum	other	fatigue
2016	Hecksteden [6]	strength: 14	strength: 9	strength: 24±2	strength: 23.51	/	RE	/	/	serum	other	fatigue
2016	Hall[122]	9	4	32±2	male: 23.2±0.3 female: 21.0±0.8	/	EE	60min	yes	plasma	HNMR	IAT
2016	Coelho[123]	4	0	/	26.2±1.2	11.5±2.0	EE	210min	no	serum	HPLC	self-paced
2016	Danaher[124]	7	0	22.9±5	25.55	/	EE	15min	yes	plasma	GC-MS	supermax
2016	Hooton[125]	3	3	/	/	/	EE	40min	/	sweat	LC-MS	self-paced
2016	Zafeiridis[126]	9	0	20.5±0.7	23.29	/	EE	64.3±4.7min	yes	plasma	HNMR	IAT

2016	Zafeiridis[126]	9	0	20.5±0.7	23.29	/	HIIT	64.3±4.7min	yes	plasma	HNMR	IAT
2017	Berton[127]	10	0	24±2	24.8±2.6	/	RE	/	yes	serum	HNMR	self-paced
2017	Julian[7]	8	4	18±3	/	/	EE	/	/	serum	other	fatigue
2017	Karl[128]	25	0	19	23	/	EE	2880min	yes	plasma	LC-MS	self-paced
2018	Souza[129]	10	10	34.5±11.94	22.64±2.54	/	EE	/	/	sweat	HNMR	aerobic
2018	Howe[130]	9	0	34±7	22.1±1.7	/	EE	557±78min	no	plasma	RPLC-MS	IAT
2018	Wiewelhove [106]	12	0	31.3±12.3	22.9±2.6	/	EE	/	/	serum	other	fatigue
2018	Stander[131]	19	12	41±12	/	/	EE	259±49min	/	serum	GC×GC-TOFMS	IAT
2020	Morville[132]	10	0	24±1	23.7±0.6	/	EE	60min	yes	plasma	UPLC-MS/MS	IAT
2020	Morville[132]	10	0	24±1	23.7±0.6	/	RE	60min	yes	plasma	UPLC-MS/MS	IAT
2020	Alzharani [133]	26	0	20.6±1.4	22.2±0.5	/	EE	50min	/	plasma/urine/saliva	LC-MS	IAT
2022	Alshuwaier [134]	43	0	20.23±0.84	21.5477551	/	EE	110min	/	serum	other	IAT
2023	Su[135]	7	7	male: 16.33±1.93 female: 16.75±1.75	male: 19.74 female: 17.96	/	HIIT	18min	/	sweat	LC-MS	fatigue

Table S8. SRM transitions and retention times of biomarkers.

Columns	Biomarkers	Standards SRM transition (m/z)	Collision energy (V)	Retention time (min)
ChromeCore HILIC-Amide column (100×2.1 mm, 3 μm)	Caffeine (IS)	195.000 → 138.000	19	0.99
	Hypoxanthine	136.950 → 109.917	21	3.65
	Hypoxanthine	134.950 → 92.000	17	3.65
	Xanthine	150.950 → 108.000	18	4.17
	Lactate	88.950 → 43.000	12	5.29
	Pyruvate	87.000 → 43.000	9	4.20
Agilent ZORBAX SB-C18 RRHT threaded column (50×4.6 mm, 1.8 μm)	Caffeine (IS)	195.000 → 138.000	19	6.80
	Isoleucine	132.000 → 86.083	10	3.78
	Phenylalanine	165.912 → 120.000	13	5.17
	Tyrosine	181.962 → 136.000	13	3.87
	Tryptophan	204.962 → 187.917	9	5.93
	Cortisol	363.037 → 121.000	25	9.06
	Testosterone	289.088 → 97.000	22	10.15

Table S9. Calibration range and coefficient.

Biomarkers	Saliva		Plasma	
	Calibration range (ng·mL^{-1})	Coefficient (r^2)	Calibration range (ng·mL^{-1})	Coefficient (r^2)
Hypoxanthine	5 - 1,000	0.9996	8 - 5,000	0.9999
Xanthine	10 - 500	0.9994	8 - 500	0.9997
Lactate	8,000 - 100,000	0.9982	20,000 - 800,000	0.9988
Pyruvate	8,000 - 100,000	0.9949	20,000 - 500,000	0.9981
Isoleucine	20 - 1,000	0.9965	200 - 10,000	0.9995
Phenylalanine	50 - 5,000	0.9993	200 - 10,000	0.9995
Tyrosine	200 - 10,000	0.9993	100 - 10,000	0.9983
Tryptophan	50 - 2,000	0.9966	100 - 10,000	0.9996
Cortisol	4 - 50	0.9970	2 - 200	0.9998
Testosterone	0.04 - 0.5	0.9981	0.5 - 20	0.9992

Table S10. Accuracy and precision of LLOQ, LQC, MQC and HQC samples in saliva.

Biomarkers	LLOQ		LQC		MQC		HQC	
	Accuracy (%)	Precision (RSD, %)	Accuracy (%)	Precision (RSD, %)	Accuracy (%)	Precision (RSD, %)	Accuracy (%)	Precision (RSD, %)
Hypoxanthine	107.13	10.15	89.20	5.43	94.47	9.54	101.47	9.54
Xanthine	87.03	7.57	86.60	9.54	104.46	7.97	103.85	9.54
Lactate	106.36	9.54	89.12	4.31	106.61	5.43	105.49	7.98
Pyruvate	97.74	10.15	102.63	9.54	96.64	9.54	101.88	9.54
Isoleucine	88.73	5.84	99.21	9.89	98.32	9.54	105.37	7.78
Phenylalanine	104.92	10.28	102.34	8.37	94.51	8.81	94.27	9.20
Tyrosine	109.90	7.15	85.37	9.54	95.55	4.76	94.91	4.78
Tryptophan	102.79	17.98	88.78	4.35	105.95	6.12	103.64	9.54
Cortisol	118.88	9.54	93.97	9.54	91.32	9.54	107.37	9.54
Testosterone	83.70	9.54	92.67	9.54	94.76	9.54	102.61	9.54

Table S11. Accuracy and precision of LLOQ, LQC, MQC and HQC samples in plasma.

Biomarkers	LLOQ		LQC		MQC		HQC	
	Accuracy (%)	Precision (RSD, %)	Accuracy (%)	Precision (RSD, %)	Accuracy (%)	Precision (RSD, %)	Accuracy (%)	Precision (RSD, %)
Hypoxanthine	102.76	14.51	90.16	7.71	100.81	4.26	97.74	4.78
Xanthine	95.61	16.03	98.34	14.58	98.48	12.54	98.68	4.67
Lactate	93.51	2.86	93.39	9.66	106.51	8.95	95.79	5.32
Pyruvate	96.20	11.27	109.65	3.08	94.97	8.95	99.00	8.95
Isoleucine	104.82	11.05	96.97	13.49	95.69	8.95	101.02	8.95
Phenylalanine	108.65	11.69	93.81	10.42	92.47	6.62	94.94	9.54
Tyrosine	99.09	9.70	99.57	9.54	102.77	9.54	93.68	8.48
Tryptophan	90.06	8.48	101.20	8.48	94.36	8.48	92.05	7.00
Cortisol	95.11	8.48	97.02	8.48	104.69	8.48	103.34	8.48
Testosterone	106.06	13.66	105.41	8.15	100.09	8.48	112.70	1.79

Table S12. LC-MS-measured concentrations of candidate biomarkers in saliva and plasma at pre-exercise and post-exercise time points in seven healthy young male volunteers are presented as mean ± SD.

Biomarkers	Participants	Saliva				Plasma			
		Pre-exercise concentration (ng/mL)	Post-exercise concentration (ng/mL)	Fold Change (FC)	Log_2(FC)	Pre-exercise concentration (ng/mL)	Post-exercise concentration (ng/mL)	Fold Change (FC)	Log_2(FC)
Hypoxanthine	1	17.35 ± 0.70	147.02 ± 5.92	8.47	3.08	1.61 ± 0.06	3802.56 ± 138.61	2361.23	11.21
	2	78.84 ± 3.17	258.04 ± 10.39	3.27	1.71	173.18 ± 6.31	1335.04 ± 48.66	7.71	2.95
	3	21.73 ± 0.87	930.85 ± 37.47	42.84	5.42	86.23 ± 3.14	2543.73 ± 92.72	29.50	4.88
	4	12.51 ± 0.50	146.36 ± 5.89	11.70	3.55	1.36 ± 0.05	308.94 ± 11.26	226.79	7.83
	5	160.92 ± 6.48	243.01 ± 9.78	1.51	0.59	559.49 ± 20.39	743.11 ± 27.09	1.33	0.41
	6	192.28 ± 7.74	271.25 ± 10.92	1.41	0.50	1.68 ± 0.06	216.87 ± 7.91	129.45	7.02
	7	38.16 ± 1.54	65.84 ± 2.65	1.73	0.79	249.06 ± 9.08	1923.75 ± 70.12	7.72	2.95
Xanthine	1	22.51 ± 0.91	97.94 ± 3.94	4.35	2.12	3.96 ± 0.14	117.87 ± 4.30	29.77	4.90
	2	18.97 ± 0.76	36.04 ± 1.45	1.90	0.93	49.84 ± 1.82	108.12 ± 3.94	2.17	1.12
	3	23.45 ± 0.94	210.74 ± 8.48	8.99	3.17	41.88 ± 1.53	140.83 ± 5.13	3.36	1.75
	4	19.32 ± 0.78	408.73 ± 16.45	21.16	4.40	11.41 ± 0.42	52.99 ± 1.93	4.64	2.22
	5	74.06 ± 2.98	158.87 ± 6.39	2.15	1.10	52.74 ± 1.92	57.52 ± 2.10	1.09	0.13
	6	17.03 ± 0.69	32.01 ± 1.29	1.88	0.91	14.35 ± 0.52	20.21 ± 0.74	1.41	0.49
	7	18.42 ± 0.74	21.01 ± 0.85	1.14	0.19	8.49 ± 0.31	78.88 ± 2.88	9.29	3.22

Lactate	1	10000.55 ± 402.52	21984.77 ± 884.89	2.20	1.14	40477.79 ± 1475.49	686688.12 ± 25031.09	16.96	4.08
	2	11058.72 ± 445.12	24328.00 ± 979.21	2.20	1.14	31888.98 ± 1162.41	396329.08 ± 14446.95	12.43	3.64
	3	8324.06 ± 335.05	6257661.46 ± 251872.20	751.76	9.55	26462.62 ± 964.61	6191343.69 ± 225686.28	233.97	7.87
	4	12147.03 ± 488.92	17785.00 ± 715.85	1.46	0.55	20328.91 ± 741.03	516012.18 ± 18809.63	25.38	4.67
	5	27832.55 ± 1120.27	2005176.31 ± 80708.77	72.04	6.17	62043.38 ± 2261.60	1983925.75 ± 72317.88	31.98	5.00
	6	9858.20 ± 396.79	12147.03 ± 488.92	1.23	0.30	77110.68 ± 2810.83	143180.93 ± 5219.22	1.86	0.89
	7	9060.32 ± 364.68	170416.38 ± 6859.30	18.81	4.23	43742.09 ± 1594.48	168610.33 ± 6146.17	3.85	1.95
Pyruvate	1	6219.59 ± 250.34	79266.41 ± 3190.49	12.74	3.67	12454.21 ± 453.98	351040.79 ± 12796.11	28.19	4.82
	2	5021.88 ± 202.13	24144.52 ± 971.82	4.81	2.27	111185.06 ± 4052.91	145218.32 ± 5293.48	1.31	0.39
	3	3756.90 ± 151.22	2409396.82 ± 96978.73	641.33	9.32	44389.64 ± 1618.09	2383862.38 ± 86896.33	53.70	5.75
	4	38323.51 ± 1542.53	92159.34 ± 3709.43	2.40	1.27	9814.70 ± 357.76	28478.13 ± 1038.08	2.90	1.54
	5	33758.27 ± 1358.78	1403544.56 ± 56492.96	41.58	5.38	150634.52 ± 5490.92	1388670.00 ± 50619.67	9.22	3.20
	6	7932.20 ± 319.27	14184.84 ± 570.94	1.79	0.84	22327.56 ± 813.88	78016.87 ± 2843.86	3.49	1.80
	7	5797.99 ± 233.37	14119.11 ± 568.30	2.44	1.28	61457.22 ± 2240.23	311138.58 ± 11341.59	5.06	2.34
Isoleucine	1	1.04 ± 0.04	642.48 ± 25.86	616.74	9.27	2784.32 ± 101.49	2412.54 ± 87.94	0.87	-0.21
	2	162.91 ± 6.56	485.16 ± 19.53	2.98	1.57	4469.61 ± 162.93	1929.50 ± 70.33	0.43	-1.21
	3	82.13 ± 3.31	266.19 ± 10.71	3.24	1.70	3329.02 ± 121.35	6597.53 ± 240.49	1.98	0.99
	4	249.05 ± 10.02	356.08 ± 14.33	1.43	0.52	691.58 ± 25.21	652.07 ± 23.77	0.94	-0.08
	5	45.84 ± 1.85	142.25 ± 5.73	3.10	1.63	2221.28 ± 80.97	2463.23 ± 89.79	1.11	0.15

	6	47.43 ± 1.91	142.94 ± 5.75	3.01	1.59	1124.28 ± 40.98	648.58 ± 23.64	0.58	-0.79
	7	3.78 ± 0.15	59.50 ± 2.39	15.73	3.98	6248.01 ± 227.75	3940.45 ± 143.64	0.63	-0.67
Phenylalanine	1	145.08 ± 5.84	3113.98 ± 125.34	21.46	4.42	9512.30 ± 346.74	7909.22 ± 288.31	0.83	-0.27
	2	968.99 ± 39.00	2627.99 ± 105.78	2.71	1.44	21146.67 ± 770.84	12946.95 ± 471.94	0.61	-0.71
	3	1116.04 ± 44.92	2088.77 ± 84.07	1.87	0.90	10335.83 ± 376.76	24703.92 ± 900.51	2.39	1.26
	4	353.89 ± 14.24	2337.99 ± 94.10	6.61	2.72	862.54 ± 31.44	1384.28 ± 50.46	1.60	0.68
	5	198.65 ± 8.00	1226.14 ± 49.35	6.17	2.63	5234.57 ± 190.81	5678.56 ± 206.99	1.08	0.12
	6	646.87 ± 26.04	1713.88 ± 68.98	2.65	1.41	4920.90 ± 179.38	2881.94 ± 105.05	0.59	-0.77
	7	67.30 ± 2.71	1374.67 ± 55.33	20.43	4.35	19338.56 ± 704.93	5306.02 ± 193.41	0.27	-1.87
Tyrosine	1	572.28 ± 23.03	5120.09 ± 206.08	8.95	3.16	4340.83 ± 158.23	4080.07 ± 148.73	0.94	-0.09
	2	1665.04 ± 67.02	5987.96 ± 241.02	3.60	1.85	8616.96 ± 314.10	4150.39 ± 151.29	0.48	-1.05
	3	4826.36 ± 194.26	7123.59 ± 286.73	1.48	0.56	4873.27 ± 177.64	8600.73 ± 313.51	1.76	0.82
	4	362.34 ± 14.58	6256.02 ± 251.81	17.27	4.11	60.29 ± 2.20	124.85 ± 4.55	2.07	1.05
	5	619.20 ± 24.92	6201.90 ± 249.63	10.02	3.32	3174.67 ± 115.72	3329.01 ± 121.35	1.05	0.07
	6	1323.79 ± 53.28	2995.33 ± 120.56	2.26	1.18	1657.50 ± 60.42	259.11 ± 9.45	0.16	-2.68
	7	387.31 ± 15.59	4246.29 ± 170.91	10.96	3.45	10478.93 ± 381.98	5747.19 ± 209.50	0.55	-0.87
Tryptophan	1	56.64 ± 2.28	318.85 ± 12.83	5.63	2.49	3258.55 ± 118.78	1320.23 ± 48.12	0.41	-1.30
	2	176.80 ± 7.12	271.54 ± 10.93	1.54	0.62	12126.76 ± 442.04	5379.34 ± 196.09	0.44	-1.17
	3	91.96 ± 3.70	187.68 ± 7.55	2.04	1.03	3996.07 ± 145.66	6719.84 ± 244.95	1.68	0.75

	4	150.07 ± 6.04	131.60 ± 5.30	0.88	-0.19	507.05 ± 18.48	260.76 ± 9.51	0.51	-0.96
	5	83.25 ± 3.35	77.86 ± 3.13	0.94	-0.10	3974.67 ± 144.88	2622.22 ± 95.58	0.66	-0.60
	6	105.06 ± 4.23	150.59 ± 6.06	1.43	0.52	1808.35 ± 65.92	1100.62 ± 40.12	0.61	-0.72
	7	59.96 ± 2.41	78.28 ± 3.15	1.31	0.38	7849.01 ± 286.11	2418.88 ± 88.17	0.31	-1.70
Cortisol	1	1.23 ± 0.05	3.97 ± 0.16	3.24	1.69	22.18 ± 0.81	92.38 ± 3.37	4.17	2.06
	2	0.68 ± 0.03	1.40 ± 0.06	2.07	1.05	8.76 ± 0.32	83.01 ± 3.03	9.48	3.24
	3	1.29 ± 0.05	3.53 ± 0.14	2.74	1.46	3.10 ± 0.11	152.42 ± 5.56	49.12	5.62
	4	4.30 ± 0.17	2.37 ± 0.10	0.55	-0.86	36.41 ± 1.33	14.15 ± 0.52	0.39	-1.36
	5	10.12 ± 0.41	1.56 ± 0.06	0.15	-2.70	89.25 ± 3.25	44.67 ± 1.63	0.50	-1.00
	6	0.68 ± 0.03	4.06 ± 0.16	5.99	2.58	24.55 ± 0.89	2.57 ± 0.09	0.10	-3.26
	7	5.88 ± 0.24	0.66 ± 0.03	0.11	-3.16	154.85 ± 5.64	24.11 ± 0.88	0.16	-2.68
Testosterone	1	0.14 ± 0.01	0.02 ± 0.00	0.18	-2.50	2.59 ± 0.09	4.50 ± 0.16	1.74	0.80
	2	0.09 ± 0.00	0.03 ± 0.00	0.35	-1.52	5.46 ± 0.20	4.57 ± 0.17	0.84	-0.25
	3	0.04 ± 0.00	0.09 ± 0.00	2.42	1.28	5.99 ± 0.22	8.46 ± 0.31	1.41	0.50
	4	0.10 ± 0.00	0.01 ± 0.00	0.08	-3.71	1.61 ± 0.06	1.75 ± 0.06	1.09	0.12
	5	0.41 ± 0.02	0.04 ± 0.00	0.09	-3.51	8.33 ± 0.30	10.03 ± 0.37	1.20	0.27
	6	0.01 ± 0.00	0.18 ± 0.01	12.81	3.68	2.56 ± 0.09	1.59 ± 0.06	0.62	-0.69
	7	0.08 ± 0.00	0.11 ± 0.00	1.33	0.41	9.86 ± 0.36	7.78 ± 0.28	0.79	-0.34

Table S13. Spearman correlation analysis of plasma and salivary biomarker responses in seven healthy young male volunteers. Correlation coefficients were calculated using individual plasma and saliva log_2(post-exercise/pre-exercise) fold changes for each candidate biomarker.

Biomarker	Spearman ρ	95% CI	P value
Hypoxanthine	0.393	-0.608 to 0.957	0.383
Xanthine	0.179	-0.750 to 1.000	0.702
Lactate	0.643	-0.222 to 1.000	0.119
Pyruvate	0.714	-0.125 to 1.000	0.071
Isoleucine	0.214	-0.647 to 1.000	0.645
Phenylalanine	-0.286	-0.957 to 0.957	0.535
Tyrosine	0.357	-0.750 to 1.000	0.432
Tryptophan	-0.071	-0.887 to 0.882	0.879
Cortisol	0.107	-0.765 to 1.000	0.819
Testosterone	-0.429	-1.000 to 0.680	0.337

Reference

1. Akimoto, T.; Nakahori, C.; Aizawa, K.; Kimura, F.; Fukubayashi, T.; Kono, I. Acupuncture and responses of immunologic and endocrine markers during competition. *Med Sci Sports Exerc* **2003**, *35*, 1296-1302. https://doi.org/10.1249/01.Mss.0000078934.07213.25.
2. Andrzejewski, M.; Konefał, M.; Podgórski, T.; Pluta, B.; Chmura, P.; Chmura, J.; Marynowicz, J.; Melka, K.; Brazaitis, M.; Kryściak, J. How training loads in the preparation and competitive period affect the biochemical indicators of training stress in youth soccer players? *PeerJ* **2022**, *10*, e13367. https://doi.org/10.7717/peerj.13367.
3. Balsalobre-Fernández, C.; Tejero-González, C.M.; del Campo-Vecino, J. Relationships between training load, salivary cortisol responses and performance during season training in middle and long distance runners. *PLoS One* **2014**, *9*, e106066. https://doi.org/10.1371/journal.pone.0106066.
4. Chang, C.W.; Huang, T.Z.; Chang, W.H.; Tseng, Y.C.; Wu, Y.T.; Hsu, M.C. Acute Garcinia mangostana (mangosteen) supplementation does not alleviate physical fatigue during exercise: a randomized, double-blind, placebo-controlled, crossover trial. *J Int Soc Sports Nutr* **2016**, *13*, 20. https://doi.org/10.1186/s12970-016-0132-0.
5. Fornaziero, A.M.; Novack, L.F.; Nascimento, V.B.; Osiecki, R. Acute Responses of Youth Elite Players to a Football Match in Terms of Blood Markers. *Sports (Basel)* **2023**, *11*. https://doi.org/10.3390/sports11120242.
6. Hecksteden, A.; Skorski, S.; Schwindling, S.; Hammes, D.; Pfeiffer, M.; Kellmann, M.; Ferrauti, A.; Meyer, T. Blood-Borne Markers of Fatigue in Competitive Athletes - Results from Simulated Training Camps. *PLoS One* **2016**, *11*, e0148810. https://doi.org/10.1371/journal.pone.0148810.
7. Julian, R.; Meyer, T.; Fullagar, H.H.; Skorski, S.; Pfeiffer, M.; Kellmann, M.; Ferrauti, A.; Hecksteden, A. Individual Patterns in Blood-Borne Indicators of Fatigue-Trait or Chance. *J Strength Cond Res* **2017**, *31*, 608-619. https://doi.org/10.1519/jsc.0000000000001390.
8. Jowko, E.; Sadowski, J.; Dlugolecka, B.; Gierczuk, D.; Opaszowski, B.; Cieslinski, I. Effects of Rhodiola rosea supplementation on mental performance, physical capacity, and oxidative stress biomarkers in healthy men. *J Sport Health Sci* **2018**, *7*, 473-480. https://doi.org/10.1016/j.jshs.2016.05.005.
9. Marcos-Serrano, M.; Olcina, G.; Crespo, C.; Brooks, D.; Timon, R. Urinary Steroid Profile in Ironman Triathletes. *J Hum Kinet* **2018**, *61*, 109-117. https://doi.org/10.1515/hukin-2017-0130.
10. Ruan, Y.; Xiang, K.F.; Zhang, H.M.; Qin, Z.; Sun, Y.; Wan, J.J.; Gu, W.; Liu, X. Orosomucoid: a promising biomarker for the assessment of exercise-induced fatigue triggered by basic combat training. *BMC Sports Sci Med Rehabil* **2022**, *14*, 100. https://doi.org/10.1186/s13102-022-00490-6.
11. Utter, A.C.; Kang, J.; Nieman, D.C.; Dumke, C.L.; McAnulty, S.R.; McAnulty, L.S. Carbohydrate attenuates perceived exertion during intermittent exercise and recovery. *Med Sci Sports Exerc* **2007**, *39*, 880-885. https://doi.org/10.1249/mss.0b013e31803174a8.

12. Wu, Q.; Fang, G.; Zhao, J.; Liu, J. Effect of Transcranial Pulsed Current Stimulation on Fatigue Delay after Medium-Intensity Training. *Int J Environ Res Public Health* **2022**, *19*. https://doi.org/10.3390/ijerph19127042.
13. Arazi, H.; Aboutalebi, S.; Taati, B.; Cholewa, J.M.; Candow, D.G. Effects of short-term betaine supplementation on muscle endurance and indices of endocrine function following acute high-intensity resistance exercise in young athletes. *J Int Soc Sports Nutr* **2022**, *19*, 1-16. https://doi.org/10.1080/15502783.2022.2041988.
14. Goto, K.; Maemura, H.; Takamatsu, K.; Ishii, N. Hormonal responses to resistance exercise after ingestion of carnosine and anserine. *J Strength Cond Res* **2011**, *25*, 398-405. https://doi.org/10.1519/JSC.0b013e3181bac43c.
15. Hough, J.P.; Papacosta, E.; Wraith, E.; Gleeson, M. Plasma and salivary steroid hormone responses of men to high-intensity cycling and resistance exercise. *J Strength Cond Res* **2011**, *25*, 23-31. https://doi.org/10.1519/JSC.0b013e3181fef8e7.
16. Räntilä, A.; Ahtiainen, J.P.; Häkkinen, K. Effects of Acute Loading Induced Fatigability, Acute Serum Hormone Responses and Training Volume to Individual Hypertrophy and Maximal Strength during 10 Weeks of Strength Training. *J Sports Sci Med* **2023**, *22*, 559-570. https://doi.org/10.52082/jssm.2023.559.
17. Shigeta, M.; Aoi, W.; Morita, C.; Soga, K.; Inoue, R.; Fukushima, Y.; Kobayashi, Y.; Kuwahata, M. Matcha green tea beverage moderates fatigue and supports resistance training-induced adaptation. *Nutr J* **2023**, *22*, 32. https://doi.org/10.1186/s12937-023-00859-4.
18. Tsuda, Y.; Yamaguchi, M.; Noma, T.; Okaya, E.; Itoh, H. Combined Effect of Arginine, Valine, and Serine on Exercise-Induced Fatigue in Healthy Volunteers: A Randomized, Double-Blinded, Placebo-Controlled Crossover Study. *Nutrients* **2019**, *11*. https://doi.org/10.3390/nu11040862.
19. West, D.J.; Cunningham, D.J.; Finn, C.V.; Scott, P.M.; Crewther, B.T.; Cook, C.J.; Kilduff, L.P. The metabolic, hormonal, biochemical, and neuromuscular function responses to a backward sled drag training session. *J Strength Cond Res* **2014**, *28*, 265-272. https://doi.org/10.1519/JSC.0b013e3182948110.
20. Bekris, E.; Bourdas, D.I.; Mylonis, E.; Ispirlidis, I.; Zacharakis, E.D.; Katis, A. Effect of 3 vs. 3 Soccer Small-Sided Game on Various Performance, Inflammatory, Muscle Damage and Hormonal Indicators in Semi-Professional Players. *Sports (Basel)* **2022**, *10*. https://doi.org/10.3390/sports10070102.
21. Reichel, T.; Bosslau, T.K.; Palmowski, J.; Eder, K.; Ringseis, R.; Mooren, F.C.; Walscheid, R.; Bothur, E.; Samel, S.; Frech, T.; et al. Reliability and suitability of physiological exercise response and recovery markers. *Sci Rep* **2020**, *10*, 11924. https://doi.org/10.1038/s41598-020-69280-9.
22. Thomas, N.E.; Leyshon, A.; Hughes, M.G.; Davies, B.; Graham, M.; Baker, J.S. The effect of anaerobic exercise on salivary cortisol, testosterone and immunoglobulin (A) in boys aged 15-16 years. *Eur J Appl Physiol* **2009**, *107*, 455-461. https://doi.org/10.1007/s00421-009-1146-y.
23. Thompson, D.; Williams, C.; Kingsley, M.; Nicholas, C.W.; Lakomy, H.K.; McArdle, F.; Jackson, M.J. Muscle soreness and damage parameters after prolonged intermittent

shuttle-running following acute vitamin C supplementation. *Int J Sports Med* **2001**, *22*, 68-75. https://doi.org/10.1055/s-2001-11358.
24. Fernandez-Lazaro, D.; Mielgo-Ayuso, J.; Del Valle Soto, M.; Adams, D.P.; Gonzalez-Bernal, J.J.; Seco-Calvo, J. The Effects of 6 Weeks of Tribulus terrestris L. Supplementation on Body Composition, Hormonal Response, Perceived Exertion, and CrossFit((R)) Performance: A Randomized, Single-Blind, Placebo-Controlled Study. *Nutrients* **2021**, *13*. https://doi.org/10.3390/nu13113969.
25. Lieberman, H.R.; Kellogg, M.D.; Bathalon, G.P. Female marine recruit training: mood, body composition, and biochemical changes. *Med Sci Sports Exerc* **2008**, *40*, S671-676. https://doi.org/10.1249/MSS.0b013e31818943b3.
26. Zhong, H.; Eungpinichpong, W.; Wang, X.; Chatchawan, U.; Wanpen, S.; Buranruk, O.; Wang, C. Effects of mechanical bed massage on biochemical markers of exercise-induced back muscle fatigue in athletes: A randomized controlled trial. *J Back Musculoskelet Rehabil* **2020**, *33*, 793-800. https://doi.org/10.3233/BMR-181347.
27. Leal Junior, E.C.; Lopes-Martins, R.A.; Vanin, A.A.; Baroni, B.M.; Grosselli, D.; De Marchi, T.; Iversen, V.V.; Bjordal, J.M. Effect of 830 nm low-level laser therapy in exercise-induced skeletal muscle fatigue in humans. *Lasers Med Sci* **2009**, *24*, 425-431. https://doi.org/10.1007/s10103-008-0592-9.
28. Timon, R.; Olcina, G.; Muñoz, D.; Maynar, J.I.; Caballero, M.J.; Maynar, M. Determination of urine steroid profile in untrained men to evaluate recovery after a strength training session. *J Strength Cond Res* **2008**, *22*, 1087-1093. https://doi.org/10.1519/JSC.0b013e31816d4542.
29. Andersson, H.; Raastad, T.; Nilsson, J.; Paulsen, G.; Garthe, I.; Kadi, F. Neuromuscular fatigue and recovery in elite female soccer: effects of active recovery. *Med Sci Sports Exerc* **2008**, *40*, 372-380. https://doi.org/10.1249/mss.0b013e31815b8497.
30. Mekonen, W.; Schwaberger, G.; Lamprecht, M.; Hofmann, P. Whole Body Substrate Metabolism during Different Exercise Intensities with Special Emphasis on Blood Protein Changes in Trained Subjects-A Pilot Study. *J Funct Morphol Kinesiol* **2023**, *8*. https://doi.org/10.3390/jfmk8030102.
31. Jiménez-Martínez, P.; Cornejo-Daza, P.J.; Sánchez-Valdepeñas, J.; Asín-Izquierdo, I.; Cano-Castillo, C.; Alix-Fages, C.; Pareja-Blanco, F.; Colado, J.C. Effects of different phenylcapsaicin doses on resistance training performance, muscle damage, protein breakdown, metabolic response, ratings of perceived exertion, and recovery: a randomized, triple-blinded, placebo-controlled, crossover trial. *J Int Soc Sports Nutr* **2023**, *20*, 2204083. https://doi.org/10.1080/15502783.2023.2204083.
32. Chen, I.F.; Wu, H.J.; Chen, C.Y.; Chou, K.M.; Chang, C.K. Branched-chain amino acids, arginine, citrulline alleviate central fatigue after 3 simulated matches in taekwondo athletes: a randomized controlled trial. *J Int Soc Sports Nutr* **2016**, *13*, 28. https://doi.org/10.1186/s12970-016-0140-0.
33. Wiewelhove, T.; Raeder, C.; Meyer, T.; Kellmann, M.; Pfeiffer, M.; Ferrauti, A. Markers for Routine Assessment of Fatigue and Recovery in Male and Female Team Sport Athletes during High-Intensity Interval Training. *PLoS One* **2015**, *10*, e0139801. https://doi.org/10.1371/journal.pone.0139801.

34. Aoi, W.; Ogaya, Y.; Takami, M.; Konishi, T.; Sauchi, Y.; Park, E.Y.; Wada, S.; Sato, K.; Higashi, A. Glutathione supplementation suppresses muscle fatigue induced by prolonged exercise via improved aerobic metabolism. *J Int Soc Sports Nutr* **2015**, *12*, 7. https://doi.org/10.1186/s12970-015-0067-x.
35. Doherty, M.; Smith, P.M.; Davison, R.C.; Hughes, M.G. Caffeine is ergogenic after supplementation of oral creatine monohydrate. *Med Sci Sports Exerc* **2002**, *34*, 1785-1792. https://doi.org/10.1097/00005768-200211000-00015.
36. Krustrup, P.; Mohr, M.; Steensberg, A.; Bencke, J.; Kjaer, M.; Bangsbo, J. Muscle and blood metabolites during a soccer game: implications for sprint performance. *Med Sci Sports Exerc* **2006**, *38*, 1165-1174. https://doi.org/10.1249/01.mss.0000222845.89262.cd.
37. Lee, M.C.; Hsu, Y.J.; Ho, C.S.; Chang, C.H.; Liu, C.W.; Huang, C.C.; Chiang, W.D. Evaluation of the Efficacy of Supplementation with Planox® Lemon Verbena Extract in Improving Oxidative Stress and Muscle Damage: A Randomized Double-Blind Controlled Trial. *Int J Med Sci* **2021**, *18*, 2641-2652. https://doi.org/10.7150/ijms.60726.
38. Dos Santos Quaresma, M.V.L.; Campos, R.; Tavares-Silva, E.; Marques, C.G.; Thomatieli-Santos, R.V. Effect of acute caffeine supplementation before intermittent high-intensity exercise on cytokine levels and psychobiological parameters: A randomized, cross-over, placebo-controlled trial. *Cytokine* **2021**, *144*, 155583. https://doi.org/10.1016/j.cyto.2021.155583.
39. Fiorenza, M.; Hostrup, M.; Gunnarsson, T.P.; Shirai, Y.; Schena, F.; Iaia, F.M.; Bangsbo, J. Neuromuscular Fatigue and Metabolism during High-Intensity Intermittent Exercise. *Med Sci Sports Exerc* **2019**, *51*, 1642-1652. https://doi.org/10.1249/mss.0000000000001959.
40. Mashiko, T.; Umeda, T.; Nakaji, S.; Sugawara, K. Position related analysis of the appearance of and relationship between post-match physical and mental fatigue in university rugby football players. *Br J Sports Med* **2004**, *38*, 617-621. https://doi.org/10.1136/bjsm.2003.007690.
41. Póvoas, S.C.; Ascensão, A.A.; Magalhães, J.; Seabra, A.F.; Krustrup, P.; Soares, J.M.; Rebelo, A.N. Analysis of fatigue development during elite male handball matches. *J Strength Cond Res* **2014**, *28*, 2640-2648. https://doi.org/10.1519/jsc.0000000000000424.
42. Stepto, N.K.; Shipperd, B.B.; Hyman, G.; McInerney, B.; Pyne, D.B. Effects of high-dose large neutral amino acid supplementation on exercise, motor skill, and mental performance in Australian Rules Football players. *Appl Physiol Nutr Metab* **2011**, *36*, 671-681. https://doi.org/10.1139/h11-073.
43. Wilson, L.J.; Cockburn, E.; Paice, K.; Sinclair, S.; Faki, T.; Hills, F.A.; Gondek, M.B.; Wood, A.; Dimitriou, L. Recovery following a marathon: a comparison of cold water immersion, whole body cryotherapy and a placebo control. *Eur J Appl Physiol* **2018**, *118*, 153-163. https://doi.org/10.1007/s00421-017-3757-z.
44. Leal Junior, E.C.; Lopes-Martins, R.A.; Frigo, L.; De Marchi, T.; Rossi, R.P.; de Godoi, V.; Tomazoni, S.S.; Silva, D.P.; Basso, M.; Filho, P.L.; et al. Effects of low-level laser therapy (LLLT) in the development of exercise-induced skeletal muscle fatigue and changes in biochemical markers related to postexercise recovery. *J Orthop Sports Phys Ther* **2010**, *40*, 524-532. https://doi.org/10.2519/jospt.2010.3294.

45. Boukhris, O.; Trabelsi, K.; Abdessalem, R.; Hsouna, H.; Ammar, A.; Glenn, J.M.; Bott, N.; Irandoust, K.; Taheri, M.; Turki, M.; et al. Effects of the 5-m Shuttle Run Test on Markers of Muscle Damage, Inflammation, and Fatigue in Healthy Male Athletes. *Int J Environ Res Public Health* **2020**, *17*. https://doi.org/10.3390/ijerph17124375.
46. Fransson, D.; Vigh-Larsen, J.F.; Fatouros, I.G.; Krustrup, P.; Mohr, M. Fatigue Responses in Various Muscle Groups in Well-Trained Competitive Male Players after a Simulated Soccer Game. *J Hum Kinet* **2018**, *61*, 85-97. https://doi.org/10.1515/hukin-2017-0129.
47. Kotsis, Y.; Mikellidi, A.; Aresti, C.; Persia, E.; Sotiropoulos, A.; Panagiotakos, D.B.; Antonopoulou, S.; Nomikos, T. A low-dose, 6-week bovine colostrum supplementation maintains performance and attenuates inflammatory indices following a Loughborough Intermittent Shuttle Test in soccer players. *Eur J Nutr* **2018**, *57*, 1181-1195. https://doi.org/10.1007/s00394-017-1401-7.
48. Radziminski, L.; Jastrzebski, Z.; Lopez-Sanchez, G.F.; Szwarc, A.; Duda, H.; Stula, A.; Paszulewicz, J.; Dragos, P. Relationships between Training Loads and Selected Blood Parameters in Professional Soccer Players during a 12-Day Sports Camp. *Int J Environ Res Public Health* **2020**, *17*. https://doi.org/10.3390/ijerph17228580.
49. Alaphilippe, A.; Mandigout, S.; Ratel, S.; Bonis, J.; Courteix, D.; Duclos, M. Longitudinal follow-up of biochemical markers of fatigue throughout a sporting season in young elite rugby players. *J Strength Cond Res* **2012**, *26*, 3376-3384. https://doi.org/10.1519/JSC.0b013e3182474687.
50. Areces, F.; Salinero, J.J.; Abian-Vicen, J.; Gonzalez-Millan, C.; Ruiz-Vicente, D.; Lara, B.; Lledo, M.; Del Coso, J. The use of compression stockings during a marathon competition to reduce exercise-induced muscle damage: are they really useful? *J Orthop Sports Phys Ther* **2015**, *45*, 462-470. https://doi.org/10.2519/jospt.2015.5863.
51. Armentano, M.J.; Brenner, A.K.; Hedman, T.L.; Solomon, Z.T.; Chavez, J.; Kemper, G.B.; Salzberg, D.; Battafarano, D.F.; Christie, D.S. The effect and safety of short-term creatine supplementation on performance of push-ups. *Mil Med* **2007**, *172*, 312-317. https://doi.org/10.7205/milmed.172.3.312.
52. Axelson, H.W.; Melberg, A.; Ronquist, G.; Askmark, H. Microdialysis and electromyography of experimental muscle fatigue in healthy volunteers and patients with mitochondrial myopathy. *Muscle Nerve* **2002**, *26*, 520-526. https://doi.org/10.1002/mus.10249.
53. Birat, A.; Bourdier, P.; Piponnier, E.; Blazevich, A.J.; Maciejewski, H.; Duche, P.; Ratel, S. Metabolic and Fatigue Profiles Are Comparable Between Prepubertal Children and Well-Trained Adult Endurance Athletes. *Front Physiol* **2018**, *9*, 387. https://doi.org/10.3389/fphys.2018.00387.
54. Broome, S.C.; Braakhuis, A.J.; Mitchell, C.J.; Merry, T.L. Mitochondria-targeted antioxidant supplementation improves 8 km time trial performance in middle-aged trained male cyclists. *J Int Soc Sports Nutr* **2021**, *18*, 58. https://doi.org/10.1186/s12970-021-00454-0.
55. Buckley, J.D.; Thomson, R.L.; Coates, A.M.; Howe, P.R.; DeNichilo, M.O.; Rowney, M.K. Supplementation with a whey protein hydrolysate enhances recovery of muscle force-generating capacity following eccentric exercise. *J Sci Med Sport* **2010**, *13*, 178-181. https://doi.org/10.1016/j.jsams.2008.06.007.

56. Chen, W.H.; Yang, W.W.; Lee, Y.H.; Wu, H.J.; Huang, C.F.; Liu, C. Acute Effects of Battle Rope Exercise on Performance, Blood Lactate Levels, Perceived Exertion, and Muscle Soreness in Collegiate Basketball Players. *J Strength Cond Res* **2020**, *34*, 2857-2866. https://doi.org/10.1519/jsc.0000000000002661.
57. Cheng, Y.C.; Lee, C.C.; Lee, M.C.; Hsu, H.Y.; Lin, J.S.; Huang, C.C.; Watanabe, K. Effects of heat-killed Lactiplantibacillus plantarum TWK10 on exercise performance, fatigue, and muscle growth in healthy male adults. *Physiol Rep* **2023**, *11*, e15835. https://doi.org/10.14814/phy2.15835.
58. Cimadevilla-Fernández-Pola, E.; Martínez-Roldán, C.; Maté-Muñoz, J.L.; Guodemar-Pérez, J.; Sánchez-Calabuig, M.A.; García-Fernández, P.; Hervás-Pérez, J.P.; Hernández-Lougedo, J. Effects of β-Alanine Supplementation on Subjects Performing High-Intensity Functional Training. *Nutrients* **2024**, *16*. https://doi.org/10.3390/nu16142340.
59. Deb, S.K.; Gough, L.A.; Sparks, S.A.; McNaughton, L.R. Sodium bicarbonate supplementation improves severe-intensity intermittent exercise under moderate acute hypoxic conditions. *Eur J Appl Physiol* **2018**, *118*, 607-615. https://doi.org/10.1007/s00421-018-3801-7.
60. Demura, S.; Morishita, K.; Yamada, T.; Yamaji, S.; Komatsu, M. Effect of L-ornithine hydrochloride ingestion on intermittent maximal anaerobic cycle ergometer performance and fatigue recovery after exercise. *Eur J Appl Physiol* **2011**, *111*, 2837-2843. https://doi.org/10.1007/s00421-011-1896-1.
61. Detanico, D.; Dal Pupo, J.; Franchini, E.; Dos Santos, S.G. Effects of successive judo matches on fatigue and muscle damage markers. *J Strength Cond Res* **2015**, *29*, 1010-1016. https://doi.org/10.1519/jsc.0000000000000746.
62. Dong, G.; Wu, J.; Hong, Y.; Li, Q.; Liu, M.; Jiang, G.; Bao, D.; Manor, B.; Zhou, J. Inhalation of Hydrogen-rich Gas before Acute Exercise Alleviates Exercise Fatigue: A Randomized Crossover Study. *Int J Sports Med* **2024**, *45*, 1014-1022. https://doi.org/10.1055/a-2318-1880.
63. Elloumi, M.; El Elj, N.; Zaouali, M.; Maso, F.; Filaire, E.; Tabka, Z.; Lac, G. IGFBP-3, a sensitive marker of physical training and overtraining. *Br J Sports Med* **2005**, *39*, 604-610. https://doi.org/10.1136/bjsm.2004.014183.
64. Farney, T.M.; Bliss, M.V.; Hearon, C.M.; Salazar, D.A. The Effect of Citrulline Malate Supplementation on Muscle Fatigue Among Healthy Participants. *J Strength Cond Res* **2019**, *33*, 2464-2470. https://doi.org/10.1519/jsc.0000000000002356.
65. Fye, H.; Pass, C.; Dickman, K.; Bredahl, E.; Eckerson, J.; Siedlik, J. The Effect of a Multi-Ingredient Pre-Workout Supplement on Time to Fatigue in NCAA Division I Cross-Country Athletes. *Nutrients* **2021**, *13*. https://doi.org/10.3390/nu13061823.
66. Gholami, F.; Ali, A.; Hasani, A.; Zarei, A. Effect of Beta-Alanine Supplementation on Exercise-Induced Cell Damage and Lactate Accumulation in Female Basketball Players: A Randomized, Double-Blind Study. *J Hum Kinet* **2022**, *83*, 99-107. https://doi.org/10.2478/hukin-2022-0034.
67. Gomez-Cabrera, M.C.; Martinez, A.; Santangelo, G.; Pallardo, F.V.; Sastre, J.; Vina, J. Oxidative stress in marathon runners: interest of antioxidant supplementation. *Br J Nutr* **2006**, *96 Suppl 1*, S31-33. https://doi.org/10.1079/bjn20061696.

68. Gorostiaga, E.M.; Navarro-Amézqueta, I.; Calbet, J.A.; Sánchez-Medina, L.; Cusso, R.; Guerrero, M.; Granados, C.; González-Izal, M.; Ibáñez, J.; Izquierdo, M. Blood ammonia and lactate as markers of muscle metabolites during leg press exercise. *J Strength Cond Res* **2014**, *28*, 2775-2785. https://doi.org/10.1519/jsc.0000000000000496.
69. Hooper, D.R.; Orange, T.; Gruber, M.T.; Darakjian, A.A.; Conway, K.L.; Hausenblas, H.A. Broad Spectrum Polyphenol Supplementation from Tart Cherry Extract on Markers of Recovery from Intense Resistance Exercise. *J Int Soc Sports Nutr* **2021**, *18*, 47. https://doi.org/10.1186/s12970-021-00449-x.
70. Howe, S.T.; Bellinger, P.M.; Driller, M.W.; Shing, C.M.; Fell, J.W. The effect of beta-alanine supplementation on isokinetic force and cycling performance in highly trained cyclists. *Int J Sport Nutr Exerc Metab* **2013**, *23*, 562-570. https://doi.org/10.1123/ijsnem.23.6.562.
71. Hsu, C.C.; Ho, M.C.; Lin, L.C.; Su, B.; Hsu, M.C. American ginseng supplementation attenuates creatine kinase level induced by submaximal exercise in human beings. *World J Gastroenterol* **2005**, *11*, 5327-5331. https://doi.org/10.3748/wjg.v11.i34.5327.
72. Kamandulis, S.; Snieckus, A.; Venckunas, T.; Aagaard, P.; Masiulis, N.; Skurvydas, A. Rapid increase in training load affects markers of skeletal muscle damage and mechanical performance. *J Strength Cond Res* **2012**, *26*, 2953-2961. https://doi.org/10.1519/JSC.0b013e318243ff21.
73. Kamitani, A.; Hara, K.; Fujii, Y.; Yoshida, S. Landing Posture in Elite Female Athletes During a Drop Vertical Jump Before and After a High-Intensity Ergometer Fatigue Protocol: A Study of 20 Japanese Women's Soccer League Players. *Orthop J Sports Med* **2023**, *11*, 23259671231171859. https://doi.org/10.1177/23259671231171859.
74. Kon, M.; Iizuka, T.; Maegawa, T.; Hashimoto, E.; Yuda, J.; Aoyanagi, T.; Akimoto, T.; Takahashi, H. Salivary secretory immunoglobulin a response of elite speed skaters during a competition period. *J Strength Cond Res* **2010**, *24*, 2249-2254. https://doi.org/10.1519/JSC.0b013e3181aff28b.
75. Konidari, Z.; Smilios, I.; Mougios, V.; Bogdanis, G.C. Effects of an Acute High Dose of Caffeine on Physiological Responses and Performance During a Strength-Focused CrossFit(®) Workout: A Randomized, Double-Blind, Crossover Study. *Nutrients* **2025**, *17*. https://doi.org/10.3390/nu17091419.
76. Kritikos, S.; Papanikolaou, K.; Draganidis, D.; Poulios, A.; Georgakouli, K.; Tsimeas, P.; Tzatzakis, T.; Batsilas, D.; Batrakoulis, A.; Deli, C.K.; et al. Effect of whey vs. soy protein supplementation on recovery kinetics following speed endurance training in competitive male soccer players: a randomized controlled trial. *J Int Soc Sports Nutr* **2021**, *18*, 23. https://doi.org/10.1186/s12970-021-00420-w.
77. Kunz, P.; Zinner, C.; Holmberg, H.C.; Sperlich, B. Intra- and Post-match Time-Course of Indicators Related to Perceived and Performance Fatigability and Recovery in Elite Youth Soccer Players. *Front Physiol* **2019**, *10*, 1383. https://doi.org/10.3389/fphys.2019.01383.
78. Lai, Z.; Lin, W.; Yan, X.; Chen, X.; Xu, G. Fatiguing freestyle swimming modifies miRNA profiles of circulating extracellular vesicles in athletes. *Eur J Appl Physiol* **2023**, *123*, 2041-2051. https://doi.org/10.1007/s00421-023-05167-7.

79. Leal Junior, E.C.; Lopes-Martins, R.A.; Rossi, R.P.; De Marchi, T.; Baroni, B.M.; de Godoi, V.; Marcos, R.L.; Ramos, L.; Bjordal, J.M. Effect of cluster multi-diode light emitting diode therapy (LEDT) on exercise-induced skeletal muscle fatigue and skeletal muscle recovery in humans. *Lasers Surg Med* **2009**, *41*, 572-577. https://doi.org/10.1002/lsm.20810.
80. Lobo, L.F.; de Morais, M.G.; Marcucci-Barbosa, L.S.; Martins-Junior, F.A.D.; Avelar, L.M.; Vieira, E.L.M.; Aidar, F.J.; Wanner, S.P.; Silva, L.S.; Noman, M.C.; et al. A Single Bout of Fatiguing Aerobic Exercise Induces Similar Pronounced Immunological Responses in Both Sexes. *Front Physiol* **2022**, *13*, 833580. https://doi.org/10.3389/fphys.2022.833580.
81. Matsubara, Y.; Shimizu, K.; Tanimura, Y.; Miyamoto, T.; Akimoto, T.; Kono, I. Effect of acupuncture on salivary immunoglobulin A after a bout of intense exercise. *Acupunct Med* **2010**, *28*, 28-32. https://doi.org/10.1136/aim.2009.001677.
82. McKenna, M.J.; Medved, I.; Goodman, C.A.; Brown, M.J.; Bjorksten, A.R.; Murphy, K.T.; Petersen, A.C.; Sostaric, S.; Gong, X. N-acetylcysteine attenuates the decline in muscle Na+,K+-pump activity and delays fatigue during prolonged exercise in humans. *J Physiol* **2006**, *576*, 279-288. https://doi.org/10.1113/jphysiol.2006.115352.
83. Medved, I.; Brown, M.J.; Bjorksten, A.R.; McKenna, M.J. Effects of intravenous N-acetylcysteine infusion on time to fatigue and potassium regulation during prolonged cycling exercise. *J Appl Physiol (1985)* **2004**, *96*, 211-217. https://doi.org/10.1152/japplphysiol.00458.2003.
84. Mikulski, T.; Dabrowski, J.; Hilgier, W.; Ziemba, A.; Krzeminski, K. Effects of supplementation with branched chain amino acids and ornithine aspartate on plasma ammonia and central fatigue during exercise in healthy men. *Folia Neuropathol* **2015**, *53*, 377-386. https://doi.org/10.5114/fn.2015.56552.
85. Mroczek, D.; Kawczynski, A.; Chmura, J. Changes of reaction time and blood lactate concentration of elite volleyball players during a game. *J Hum Kinet* **2011**, *28*, 73-78. https://doi.org/10.2478/v10078-011-0024-y.
86. Okada, H.; Yoshida, N.; Kakuma, T.; Toyomasu, K. Effect of Chlorella Ingestion on Oxidative Stress and Fatigue Symptoms in Healthy Men. *Kurume Med J* **2018**, *64*, 83-90. https://doi.org/10.2739/kurumemedj.MS644001.
87. Qu, C.; Xu, M.; Lorenzo, S.; Huang, P.; Rao, Z.; Geng, X.; Zhao, J. Effects of mild hyperbaric oxygen therapy on timing sequence recovery of muscle fatigue in chinese university male athletes. *J Exerc Sci Fit* **2024**, *22*, 305-315. https://doi.org/10.1016/j.jesf.2024.04.005.
88. Sanchez-Medina, L.; Gonzalez-Badillo, J.J. Velocity loss as an indicator of neuromuscular fatigue during resistance training. *Med Sci Sports Exerc* **2011**, *43*, 1725-1734. https://doi.org/10.1249/MSS.0b013e318213f880.
89. Santana, J.O.; de Freitas, M.C.; Dos Santos, D.M.; Rossi, F.E.; Lira, F.S.; Rosa-Neto, J.C.; Caperuto, E.C. Beta-Alanine Supplementation Improved 10-km Running Time Trial in Physically Active Adults. *Front Physiol* **2018**, *9*, 1105. https://doi.org/10.3389/fphys.2018.01105.
90. Shiu, Y.J.; Chen, C.H.; Tao, W.S.; Nai, H.F.; Yu, C.Y.; Chiu, C.H. Acute ingestion of caffeinated chewing gum reduces fatigue index and improves 400-meter performance in

trained sprinters: a double-blind crossover trial. *J Int Soc Sports Nutr* **2024**, *21*, 2414871. https://doi.org/10.1080/15502783.2024.2414871.

91. da Silva, B.V.C.; Simim, M.A.M.; da Silva, R.B.; Mendes, E.L.; Ide, B.N.; Marocolo, M.; Martin, J.S.; Mota, G.R. Time Course of Recovery for Performance Attributes and Circulating Markers of Muscle Damage Following a Rugby Union Match in Amateur Athletes. *Sports (Basel)* **2020**, *8*. https://doi.org/10.3390/sports8050064.
92. de Souza, R.F.; de Matos, D.G.; Lopes Dos Santos, J.; Andrade Lima, C.; Reis Pires Ferreira, A.; Moreno, G.; Santos Oliveira, A.; Dutra Pereira, D.; Knechtle, B.; Aidar, F.J. Effects of ibuprofen during 42-km trail running on oxidative stress, muscle fatigue, muscle damage and performance: a randomized controlled trial. *Res Sports Med* **2024**, *32*, 400-410. https://doi.org/10.1080/15438627.2022.2122826.
93. Spanidis, Y.; Goutzourelas, N.; Stagos, D.; Mpesios, A.; Priftis, A.; Bar-Or, D.; Spandidos, D.A.; Tsatsakis, A.M.; Leon, G.; Kouretas, D. Variations in oxidative stress markers in elite basketball players at the beginning and end of a season. *Exp Ther Med* **2016**, *11*, 147-153. https://doi.org/10.3892/etm.2015.2843.
94. Staiano, W.; Bosio, A.; Piazza, G.; Romagnoli, M.; Invernizzi, P.L. Kayaking performance is altered in mentally fatigued young elite athletes. *J Sports Med Phys Fitness* **2019**, *59*, 1253-1262. https://doi.org/10.23736/s0022-4707.18.09051-5.
95. Staniszewski, M.; Tkaczyk, J.; Kęska, A.; Zybko, P.; Mróz, A. Effect of rest duration between sets on fatigue and recovery after short intense plyometric exercise. *Sci Rep* **2024**, *14*, 15080. https://doi.org/10.1038/s41598-024-66146-2.
96. Stoter, I.K.; MacIntosh, B.R.; Fletcher, J.R.; Pootz, S.; Zijdewind, I.; Hettinga, F.J. Pacing Strategy, Muscle Fatigue, and Technique in 1500-m Speed-Skating and Cycling Time Trials. *Int J Sports Physiol Perform* **2016**, *11*, 337-343. https://doi.org/10.1123/ijspp.2014-0603.
97. Tsoukos, A.; Krzysztofik, M.; Wilk, M.; Zajac, A.; Panagiotopoulos, M.G.; Psarras, II; Petraki, D.P.; Terzis, G.; Bogdanis, G.C. Fatigue and Metabolic Responses during Repeated Sets of Bench Press Exercise to Exhaustion at Different Ranges of Motion. *J Hum Kinet* **2024**, *91*, 61-76. https://doi.org/10.5114/jhk/185524.
98. Varela-Olalla, D.; Romero-Caballero, A.; Del Campo-Vecino, J.; Balsalobre-Fernandez, C. A Cluster Set Protocol in the Half Squat Exercise Reduces Mechanical Fatigue and Lactate Concentrations in Comparison with A Traditional Set Configuration. *Sports (Basel)* **2020**, *8*. https://doi.org/10.3390/sports8040045.
99. Varley, I.; Lewin, R.; Needham, R.; Thorpe, R.T.; Burbeary, R. Association between Match Activity Variables, Measures of Fatigue and Neuromuscular Performance Capacity Following Elite Competitive Soccer Matches. *J Hum Kinet* **2017**, *60*, 93-99. https://doi.org/10.1515/hukin-2017-0093.
100. Venhorst, A.; Micklewright, D.P.; Noakes, T.D. The Psychophysiological Regulation of Pacing Behaviour and Performance Fatigability During Long-Distance Running with Locomotor Muscle Fatigue and Exercise-Induced Muscle Damage in Highly Trained Runners. *Sports Med Open* **2018**, *4*, 29. https://doi.org/10.1186/s40798-018-0143-2.
101. Vermeulen, S.; Bleecker, C.; Spanhove, V.; Boone, J.; Willems, T.; Vanrenterghem, J.; Roosen, P.; Ridder, R. The Utility of High-Intensity, Intermittent Exercise Protocols to

Induce Fatigue for Screening Purposes in Jump-Landing Sports. *J Hum Kinet* **2024**, *93*, 69-80. https://doi.org/10.5114/jhk/183537.
102. Volodchenko, O.A.; Podrigalo, L.V.; Iermakov, S.S.; Zychowska, M.T.; Jagiello, W. The Usefulness of Performing Biochemical Tests in the Saliva of Kickboxing Athletes in the Dynamic of Training. *Biomed Res Int* **2019**, *2019*, 2014347. https://doi.org/10.1155/2019/2014347.
103. Warber, J.P.; Patton, J.F.; Tharion, W.J.; Zeisel, S.H.; Mello, R.P.; Kemnitz, C.P.; Lieberman, H.R. The effects of choline supplementation on physical performance. *Int J Sport Nutr Exerc Metab* **2000**, *10*, 170-181. https://doi.org/10.1123/ijsnem.10.2.170.
104. White, G.E.; Wells, G.D. The effect of on-hill active recovery performed between runs on blood lactate concentration and fatigue in alpine ski racers. *J Strength Cond Res* **2015**, *29*, 800-806. https://doi.org/10.1519/jsc.0000000000000677.
105. Whyte, G.P.; George, K.; Sharma, S.; Lumley, S.; Gates, P.; Prasad, K.; McKenna, W.J. Cardiac fatigue following prolonged endurance exercise of differing distances. *Med Sci Sports Exerc* **2000**, *32*, 1067-1072. https://doi.org/10.1097/00005768-200006000-00005.
106. Wiewelhove, T.; Schneider, C.; Döweling, A.; Hanakam, F.; Rasche, C.; Meyer, T.; Kellmann, M.; Pfeiffer, M.; Ferrauti, A. Effects of different recovery strategies following a half-marathon on fatigue markers in recreational runners. *PLoS One* **2018**, *13*, e0207313. https://doi.org/10.1371/journal.pone.0207313.
107. Wu, C.L.; Shih, M.C.; Yang, C.C.; Huang, M.H.; Chang, C.K. Sodium bicarbonate supplementation prevents skilled tennis performance decline after a simulated match. *J Int Soc Sports Nutr* **2010**, *7*, 33. https://doi.org/10.1186/1550-2783-7-33.
108. Yan, B.; Yao, S.; Zhang, J.; Li, C.; Han, T.; Hu, Q.; Lv, K. Effects of asymmetric load bench press offset training on muscle activation levels and exercise-induced fatigue in collegiate bodybuilders. *Front Physiol* **2025**, *16*, 1592477. https://doi.org/10.3389/fphys.2025.1592477.
109. Yokoi, Y.; Yanagihashi, R.; Morishita, K.; Goto, N.; Fujiwara, T.; Abe, K. Recovery effects of repeated exposures to normobaric hyperoxia on local muscle fatigue. *J Strength Cond Res* **2014**, *28*, 2173-2179. https://doi.org/10.1519/jsc.0000000000000386.
110. Zory, R.; Millet, G.; Schena, F.; Bortolan, L.; Rouard, A. Fatigue induced by a cross-country skiing KO sprint. *Med Sci Sports Exerc* **2006**, *38*, 2144-2150. https://doi.org/10.1249/01.mss.0000235354.86189.7e.
111. Enea, C.; Seguin, F.; Petitpas-Mulliez, J.; Boildieu, N.; Boisseau, N.; Delpech, N.; Diaz, V.; Eugène, M.; Dugué, B. (1)H NMR-based metabolomics approach for exploring urinary metabolome modifications after acute and chronic physical exercise. *Anal Bioanal Chem* **2010**, *396*, 1167-1176. https://doi.org/10.1007/s00216-009-3289-4.
112. Pechlivanis, A.; Kostidis, S.; Saraslanidis, P.; Petridou, A.; Tsalis, G.; Mougios, V.; Gika, H.G.; Mikros, E.; Theodoridis, G.A. (1)H NMR-based metabonomic investigation of the effect of two different exercise sessions on the metabolic fingerprint of human urine. *J Proteome Res* **2010**, *9*, 6405-6416. https://doi.org/10.1021/pr100684t.
113. Nieman, D.C.; Shanely, R.A.; Gillitt, N.D.; Pappan, K.L.; Lila, M.A. Serum metabolic signatures induced by a three-day intensified exercise period persist after 14 h of recovery in runners. *J Proteome Res* **2013**, *12*, 4577-4584. https://doi.org/10.1021/pr400717j.

114. Ra, S.G.; Maeda, S.; Higashino, R.; Imai, T.; Miyakawa, S. Metabolomics of salivary fatigue markers in soccer players after consecutive games. *Appl Physiol Nutr Metab* **2014**, *39*, 1120-1126. https://doi.org/10.1139/apnm-2013-0546.
115. Mukherjee, K.; Edgett, B.A.; Burrows, H.W.; Castro, C.; Griffin, J.L.; Schwertani, A.G.; Gurd, B.J.; Funk, C.D. Whole blood transcriptomics and urinary metabolomics to define adaptive biochemical pathways of high-intensity exercise in 50-60 year old masters athletes. *PLoS One* **2014**, *9*, e92031. https://doi.org/10.1371/journal.pone.0092031.
116. Peake, J.M.; Tan, S.J.; Markworth, J.F.; Broadbent, J.A.; Skinner, T.L.; Cameron-Smith, D. Metabolic and hormonal responses to isoenergetic high-intensity interval exercise and continuous moderate-intensity exercise. *Am J Physiol Endocrinol Metab* **2014**, *307*, E539-552. https://doi.org/10.1152/ajpendo.00276.2014.
117. Daskalaki, E.; Blackburn, G.; Kalna, G.; Zhang, T.; Anthony, N.; Watson, D.G. A study of the effects of exercise on the urinary metabolome using normalisation to individual metabolic output. *Metabolites* **2015**, *5*, 119-139. https://doi.org/10.3390/metabo5010119.
118. Breit, M.; Netzer, M.; Weinberger, K.M.; Baumgartner, C. Modeling and Classification of Kinetic Patterns of Dynamic Metabolic Biomarkers in Physical Activity. *PLoS Comput Biol* **2015**, *11*, e1004454. https://doi.org/10.1371/journal.pcbi.1004454.
119. Pechlivanis, A.; Papaioannou, K.G.; Tsalis, G.; Saraslanidis, P.; Mougios, V.; Theodoridis, G.A. Monitoring the Response of the Human Urinary Metabolome to Brief Maximal Exercise by a Combination of RP-UPLC-MS and (1)H NMR Spectroscopy. *J Proteome Res* **2015**, *14*, 4610-4622. https://doi.org/10.1021/acs.jproteome.5b00470.
120. Nieman, D.C.; Gillitt, N.D.; Sha, W.; Meaney, M.P.; John, C.; Pappan, K.L.; Kinchen, J.M. Metabolomics-Based Analysis of Banana and Pear Ingestion on Exercise Performance and Recovery. *J Proteome Res* **2015**, *14*, 5367-5377. https://doi.org/10.1021/acs.jproteome.5b00909.
121. Muhsen Ali, A.; Burleigh, M.; Daskalaki, E.; Zhang, T.; Easton, C.; Watson, D.G. Metabolomic Profiling of Submaximal Exercise at a Standardised Relative Intensity in Healthy Adults. *Metabolites* **2016**, *6*. https://doi.org/10.3390/metabo6010009.
122. Andersson Hall, U.; Edin, F.; Pedersen, A.; Madsen, K. Whole-body fat oxidation increases more by prior exercise than overnight fasting in elite endurance athletes. *Appl Physiol Nutr Metab* **2016**, *41*, 430-437. https://doi.org/10.1139/apnm-2015-0452.
123. Coelho, W.S.; Viveiros de Castro, L.; Deane, E.; Magno-França, A.; Bassini, A.; Cameron, L.C. Investigating the Cellular and Metabolic Responses of World-Class Canoeists Training: A Sportomics Approach. *Nutrients* **2016**, *8*. https://doi.org/10.3390/nu8110719.
124. Danaher, J.; Gerber, T.; Wellard, R.M.; Stathis, C.G.; Cooke, M.B. The use of metabolomics to monitor simultaneous changes in metabolic variables following supramaximal low volume high intensity exercise. *Metabolomics* **2016**, *12*, 13. https://doi.org/10.1007/s11306-015-0883-7.
125. Hooton, K.; Han, W.; Li, L. Comprehensive and Quantitative Profiling of the Human Sweat Submetabolome Using High-Performance Chemical Isotope Labeling LC-MS. *Anal Chem* **2016**, *88*, 7378-7386. https://doi.org/10.1021/acs.analchem.6b01930.
126. Zafeiridis, A.; Chatziioannou, A.C.; Sarivasiliou, H.; Kyparos, A.; Nikolaidis, M.G.; Vrabas, I.S.; Pechlivanis, A.; Zoumpoulakis, P.; Baskakis, C.; Dipla, K.; et al. Global Metabolic

Stress of Isoeffort Continuous and High Intensity Interval Aerobic Exercise: A Comparative (1)H NMR Metabonomic Study. *J Proteome Res* **2016**, *15*, 4452-4463. https://doi.org/10.1021/acs.jproteome.6b00545.
127. Berton, R.; Conceição, M.S.; Libardi, C.A.; Canevarolo, R.R.; Gáspari, A.F.; Chacon-Mikahil, M.P.; Zeri, A.C.; Cavaglieri, C.R. Metabolic time-course response after resistance exercise: A metabolomics approach. *J Sports Sci* **2017**, *35*, 1211-1218. https://doi.org/10.1080/02640414.2016.1218035.
128. Karl, J.P.; Margolis, L.M.; Murphy, N.E.; Carrigan, C.T.; Castellani, J.W.; Madslien, E.H.; Teien, H.K.; Martini, S.; Montain, S.J.; Pasiakos, S.M. Military training elicits marked increases in plasma metabolomic signatures of energy metabolism, lipolysis, fatty acid oxidation, and ketogenesis. *Physiol Rep* **2017**, *5*. https://doi.org/10.14814/phy2.13407.
129. Souza, S.L.; Graça, G.; Oliva, A. Characterization of sweat induced with pilocarpine, physical exercise, and collected passively by metabolomic analysis. *Skin Res Technol* **2018**, *24*, 187-195. https://doi.org/10.1111/srt.12412.
130. Howe, C.C.F.; Alshehri, A.; Muggeridge, D.; Mullen, A.B.; Boyd, M.; Spendiff, O.; Moir, H.J.; Watson, D.G. Untargeted Metabolomics Profiling of an 80.5 km Simulated Treadmill Ultramarathon. *Metabolites* **2018**, *8*. https://doi.org/10.3390/metabo8010014.
131. Stander, Z.; Luies, L.; Mienie, L.J.; Keane, K.M.; Howatson, G.; Clifford, T.; Stevenson, E.J.; Loots, D.T. The altered human serum metabolome induced by a marathon. *Metabolomics* **2018**, *14*, 150. https://doi.org/10.1007/s11306-018-1447-4.
132. Morville, T.; Sahl, R.E.; Moritz, T.; Helge, J.W.; Clemmensen, C. Plasma Metabolome Profiling of Resistance Exercise and Endurance Exercise in Humans. *Cell Rep* **2020**, *33*, 108554. https://doi.org/10.1016/j.celrep.2020.108554.
133. Alzharani, M.A.; Alshuwaier, G.O.; Aljaloud, K.S.; Al-Tannak, N.F.; Watson, D.G. Metabolomics profiling of plasma, urine and saliva after short term training in young professional football players in Saudi Arabia. *Sci Rep* **2020**, *10*, 19759. https://doi.org/10.1038/s41598-020-75755-6.
134. Alshuwaier, G.O.; Ghazzawi, H.A.; Alaqil, A.I.; Alsharif, Y.R.; Bursais, A.K.; Amawi, A.T. Different training sessions impact on serum protein profile of Saudi professional soccer players. *Niger J Clin Pract* **2022**, *25*, 1287-1294. https://doi.org/10.4103/njcp.njcp_72_22.
135. Meihua, S.; Jiahui, J.; Yujia, L.; Shuang, Z.; Jingjing, Z. Research on sweat metabolomics of athlete's fatigue induced by high intensity interval training. *Front Physiol* **2023**, *14*, 1269885. https://doi.org/10.3389/fphys.2023.1269885.
